# Supplementary material for: Altercrasins A–E, Decalin Derivatives, from a Sea-Urchin-Derived Alternaria sp.: Isolation and Structural Analysis Including Stereochemistry
Source: Mar Drugs. 2019 Apr 11;17(4):218. doi: 10.3390/md17040218 (PMC6521173; doi:10.3390/md17040218)
Supplement: Supplementary file 1 [file marinedrugs-17-00218-s001.zip › marinedrugs-480817 Suppl/S.M-R1/SM-vol1.pdf]

Supplementary Materials

Altercrasins A–E, decalin derivatives, from a sea-urchin-derived *Alternaria* sp.: Isolation and structural analysis including stereochemistry

Takeshi Yamada, Asumi Tanaka, Tatsuo Nehira, Takumi Nishii and Takashi Kikuchi

The Table of Contents

**Table S0**	**Spectral data including 2D NMR data for 1**	**2**
**Table S1**	**Spectral data including 2D NMR data for 2**	**3**
**Table S2**	**Spectral data including 2D NMR data for 3**	**4**
**Table S3**	**Spectral data including 2D NMR data for 4**	**5**
**Table S4**	**Spectral data including 2D NMR data for 5**	**6**
**Figure S1**	**^{1}H and ^{13}C NMR spectrum of 1 in acetone-d6**	**7**
**Figure S2**	**^{1}H-^{1}H COSY of 1**	**13**
**Figure S3**	**NOESY of 1**	**15**
**Figure S4**	**HMQC of 1**	**17**
**Figure S5**	**HMBC of 1**	**19**
**Figure S6**	**FABMS of 1**	**23**
**Figure S7**	**^{1}H and ^{13}C NMR spectrum of 2 in acetone-d6**	**25**
**Figure S8**	**^{1}H-^{1}H COSY of 2**	**32**
**Figure S9**	**NOESY of 2**	**34**
**Figure S10**	**HMQC of 2**	**36**
**Figure S11**	**HMBC of 2**	**38**
**Figure S12**	**FABMS of 2**	**41**
**Figure S13**	**^{1}H and ^{13}C NMR spectrum of 3 in acetone-d6**	**43**
**Figure S14**	**^{1}H-^{1}H COSY of 3**	**50**
**Figure S15**	**NOESY of 3**	**52**
**Figure S16**	**HMQC of 3**	**54**
**Figure S17**	**HMBC of 3**	**56**
**Figure S18**	**FABMS of 3**	**59**
**Figure S19**	**^{1}H and ^{13}C NMR spectrum of 4 in acetone-d6**	**61**
**Figure S20**	**^{1}H-^{1}H COSY of 4**	**68**

Figure S21 **NOESY of 4** **70**

Figure S22 **HMQC of 4** **72**

Figure S23 **HMBC of 4** **74**

Figure S24 **FABMS of 4** **77**

Figure S25 **^{1}H and ^{13}C NMR spectrum of 5 in acetone-d6** **79**

Figure S26 **^{1}H-^{1}H COSY of 5** **86**

Figure S27 **NOESY of 5** **88**

Figure S28 **HMQC of 5** **90**

Figure S29 **HMBC of 5** **92**

Figure S30 **FABMS of 5** **95**

Table S0 NMR spectral data of **1** in acetone-d_6

Position	δ_H[a]		J/Hz	^1H-^1H COSY	NOESY[b]	δ_C		HMBC (C)[c]
1α	1.55	dq	12.8 (1β), 3.8 (2α, 2β, 10)	1β, 2α, 2β, 10		25.9	(t)	2, 10
1β	1.12	qd	12.8 (1α, 2α, 10), 3.8 (2β)	1α, 2α, 10	3, 5, 24			
2α	0.90	m		1α, 1β, 2β, 3	4α, 10, 23	36.0	(t)	
2β	1.74	ddt	12.8 (2α), 5.3 (3), 3.8 (1α, 1β)	1α, 2α	23			
3	1.48	m		2α, 4α, 4β, 23	1β, 5	33.5	(d)	
4α	0.82	q	12.8 (3, 4β, 5)	3, 4β, 5	2α, 10, 23	42.6	(t)	2, 3, 5, 6, 10, 23
4β	1.87	ddd	12.8 (4α), 5.5 (4), 3.2 (5)	3, 4α, 5	6, 23			
5	1.92	br t	12.8 (4α, 10)	4α, 4β, 10	1β, 3, 24	37.4	(d)	10
6	5.55	d	11.3 (7)	7	4β	133.2	(d)	4, 5, 7, 8, 10
7	5.70	ddd	11.3 (6), 4.8 (8), 2.5	6, 8		125.3	(d)	5, 6, 8, 13
8	2.65	ddt	11.8 (13), 4.8 (7), 1.8	7, 13	14, 24	49.9	(d)	6, 7, 9, 10, 13, 14, 24
9						52.9	(s)	
10	1.49	td	12.8 (1β, 5), 3.8 (1α)	1α, 1β, 5	2α, 4α, 13	38.7	(d)	5, 24
11						211.6	(s)	
12						74.0	(s)	
13	3.16	dd	11.8 (8), 9.0 (14)	8, 14	10, 15	52.8	(d)	7, 8, 12, 14, 15, 18, 22
14	5.62	ddd	15.5 (15), 9.0 (13), 1.8	13, 15	8, 16, 17, 19	124.3	(d)	8, 12, 13, 15, 16
15	5.73	dd	15.5 (14), 5.5 (16)	14, 16	13, 17	141.9	(d)	13, 14, 16, 17
16	4.21	m		15, 17, 16-OH	14	68.8	(d)	14
17	1.16	d	6.8 (16)	16	14, 15	24.2	(q)	15, 16
18						207.1	(s)	
19	3.66	d	7.0 (20)	20	14, 21	70.4	(d)	18, 20, 21, 22
20	3.99	m		19, 21, 20-OH	NH	67.8	(d)	18
21	1.25	d	6.2 (20)	20	19, NH	20.4	(q)	19, 20
22						170.7	(s)	
23	0.91	d	6.5 (3)	3	2α, 2β, 4α, 4β	22.6	(q)	2, 3, 4
24	0.95	s			1β, 5, 8	16.0	(q)	8, 9, 10, 11
16-OH	3.79	d	5.0 (16)	16				15, 16, 17
20-OH	4.17	d	5.5 (20)	20				19
NH	8.01	br s			20, 21			

a ^1H chemical shift values (δ ppm from $SiMe_4$) followed by multiplicity and then the coupling constants (J/Hz). Figures in parentheses indicate the proton coupling with that position. b The correlations with geminal and vicinal protones are removed. c Long range ^1H-^{13}C correlations from H to C observed in the HMBC experiment.

Table S1 NMR spectral data of **2** in acetne-d_6

Position	δ_H[a]		J/Hz	^{1}H-^{1}H COSY	NOESY[b]	δ_C		HMBC (C)[c]
1α	1.52	dq	13.2 (1β), 3.6 (2α, 2β, 10)	1β, 10		25.9	(t)	10
1β	1.13	qd	13.2 (1α, 2α, 10), 3.6 (2β)	1α	3, 5, 24			
2α	0.90	m		2β, 3	10, 23	36.0	(t)	
2β	1.75	ddt	12.6 (2α), 5.4 (3), 3.6 (1α, 1β)	2α	23			
3	1.49	m		2α, 4α, 23	1β, 5	33.5	(d)	
4α	0.83	q	12.0 (3, 4β, 5)	3, 4β, 5	10, 23	42.7	(t)	2, 3, 5, 6, 10, 23
4β	1.88	ddd	12.0 (4α), 5.4 (3), 3.5 (5)	4α, 5	6, 23			
5	1.93	m		4α, 4β, 10	1β, 3, 24	37.5	(d)	3, 6, 10
6	5.54	d	10.2 (7)	7	4β	132.9	(d)	4, 5, 7, 8, 10
7	5.69	ddd	10.2 (6), 4.8 (8), 2.4	6, 8		125.7	(d)	5, 8, 9
8	2.74	ddt	12.0 (13), 4.8 (7), 1.8	7, 13	14, 24	49.7	(d)	6, 7, 9, 10, 13, 14, 24
9						52.9	(s)	
10	1.40	td	13.2 (1α, 5), 3.0 (1α)	1β, 5	2α, 4α, 13	39.0	(d)	1, 24
11						211.7	(s)	
12						74.2	(s)	
13	3.01	dd	12.0 (8), 9.0 (14)	8, 14	10, 15	52.5	(d)	7, 8, 12, 14, 15, 18, 22
14	5.82	ddd	15.0 (15), 9.0 (13), 1.8	13, 15	8, 16, 17, 19	125.5	(d)	8, 12, 13, 16
15	5.59	ddd	15.0 (14), 5.4 (16), 0.6	14, 16	13, 17	140.8	(d)	13, 16, 17
16	4.17	m		15, 17, 16-OH	14, 20	68.0	(d)	14, 15, 17
17	1.14	d	6.6 (16)	16	14, 15	23.9	(q)	15, 16
18						207.8	(s)	
19	3.93	d	4.2 (20)	20	21, NH	69.7	(d)	18, 20, 21, 22
20	3.98	m		19, 21	16	67.4	(d)	
21	1.27	d	6.0 (20)	20	19, NH	20.7	(q)	19, 20
22						170.2	(s)	
23	0.92	d	6.6 (3)	3	2α, 2β, 4α, 4β	22.6	(q)	2, 3, 4
24	0.99	s			1β, 5, 8	16.1	(q)	8, 9, 10, 11
16-OH	3.47	br s		16				
20-OH	4.02	br s						
NH	8.02	br s			19, 21			

a ^{1}H chemical shift values (δ ppm from $SiMe_4$) followed by multiplicity and then the coupling constants (J/Hz). Figures in parentheses indicate the proton coupling with that position. b The correlations with geminal and vicinal protones are removed. c Long range ^{1}H-^{13}C correlations from H to C observed in the HMBC experiment.

Table S2 NMR spectral data of **3** in acetne-d_6

Position	δ_H [a]		J/Hz	^1H-^1H COSY	NOESY [b]	δ_C		HMBC (C) [c]
1α	1.80	dq	12.6 (1β), 3.6 (2α, 2β, 10)	1β, 2α, 10		26.0	(t)	
1β	1.20	qd	12.6 (1α, 2α, 10), 3.6 (2β)	1α, 2α, 2β, 10	3, 5, 24			2, 5, 10
2α	0.87	qd	12.6 (1β, 2α, 3), 3.6 (1α)	1α, 1β, 2β, 3	10	36.2	(t)	1, 3, 10, 23
2β	1.74	ddt	12.6 (2α), 5.4 (3), 3.6 (1α, 1β)	1β, 2α				
3	1.49	m		2α, 23	1β, 5	33.7	(d)	23
4α	0.79	q	12.6 (3, 4β, 5)	4β, 5	10, 23	43.0	(t)	2, 3, 5, 6, 10, 23
4β	1.87	ddd	12.6 (4α), 5.4 (3), 3.6 (5)	4α, 5	6, 23			2, 5
5	1.95	br t	12.6 (4α, 10)	4α, 4β, 10	1β, 3, 24	37.5	(d)	4, 6
6	5.57	d	9.6 (7)	7	4β	133.1	(d)	4, 5, 8, 10
7	5.97	ddd	9.6 (6), 4.8 (8), 3.0	6, 8	14	126.1	(d)	5, 8, 9
8	3.34	ddt	12.6 (13), 4.8 (7), 0.9	7, 13	24	45.3	(d)	6, 7, 9, 10, 13, 24
9						53.4	(s)	
10	1.35	td	12.6 (1β, 5), 3.6 (1α)	1α, 1β, 5	2α, 4α, 13	40.4	(d)	5, 24
11						211.3	(s)	
12						74.7	(s)	
13	2.83	ddd	12.6 (8), 5.4 (14), 2.4	8, 14	10, 17	45.5	(d)	
14	6.42	ddd	8.4 (15), 5.4 (13), 2.4	13, 15	7	133.6	(d)	8, 16
15	5.83	ddd	8.4 (14), 5.4 (16), 2.4	14, 16	17	134.3	(d)	13, 16, 17, 18
16	2.48	m		15, 17		43.7	(d)	14, 18
17	1.20	d	7.2 (16)	16	13, 15	15.3	(q)	15, 16, 18
18						81.2	(s)	
19						173.3	(d)	18, 20, 21, 22
22						177.5	(s)	
23	0.89	d	6.6 (3)	3	4α, 4β	22.8	(q)	2, 3, 4
24	1.05	s			1β, 5, 8	17.3	(q)	8, 9, 10, 11

a ^1H chemical shift values (δ ppm from $SiMe_4$) followed by multiplicity and then the coupling constants (J/Hz). Figures in parentheses indicate the proton coupling with that position. b The correlations with geminal and vicinal protones are removed. c Long range ^1H-^{13}C correlations from H to C observed in the HMBC experiment.

Table S3 NMR spectral data of **4** in acetne-d_6

Position	δ_H[a]		J/Hz	^{1}H-^{1}H COSY	NOESY[b]	δ_C		HMBC (C)[c]
1α	1.67	m		1β		27.6	(t)	
1β	1.20	qd	12.6 (1α, 2α, 10), 3.6 (2β)	1α, 2β, 10	5, 24			
2α	0.85	qd	12.6 (1β, 2β, 3), 3.6 (1α)	2β, 3	23	36.1	(t)	1
2β	1.72	m		2α	23			
3	1.49	m		2α, 23	1β, 5	33.5	(d)	
4α	0.79	q	12.6 (3, 4β, 5)	4β, 5	10, 23	42.6	(t)	2, 3, 5, 10, 23
4β	1.85	ddd	12.6 (4α), 5.4 (3), 3.6 (5)	4α	6, 23			
5	2.08	m		4α, 10	1β, 3, 24	39.1	(d)	
6	5.48	d	10.2 (7)	7	4β	133.3	(d)	4, 5, 8, 10
7	6.04	dd	10.2 (6), 3.0	6	14	127.7	(d)	5, 8, 9, 10
8						141.5	(s)	
9						50.7	(s)	
10	1.70	m		1β, 5	4α	44.7	(d)	5, 24
11						208.4	(s)	
12						70.5	(s)	
13	3.71	d	9.6 (15)	14, 15	16, 24	43.6	(d)	8, 12, 14, 15, 16, 22
14	5.52	d	1.8	13	7, 15	124.6	(d)	7, 9, 12, 13, 15
15	5.37	ddq	16.8 (16), 9.0 (13), 1.8	13, 16	14, 17	128.4	(d)	12, 16, 17
16	5.57	dq	16.8 (15), 6.6 (17)	15, 17	13	131.0	(d)	13, 17
17	1.62	d	6.6 (16)	16	15	18.0	(q)	15, 16
18						208.4	(s)	
19	3.49	d	7.2 (20)	20	21, NH	69.9	(d)	18, 20, 21, 22
20	3.99	m		19, 21		68.1	(d)	
21	1.28	d	6.6 (20)	20	19, NH	20.2	(q)	19, 20
22						168.1	(s)	
23	0.89	d	6.6 (3)	3	2α, 2β, 4α, 4β	22.6	(q)	2, 3, 4
24	1.12	s			1β, 5, 13	17.4	(q)	8, 9, 10, 11
20-OH	4.06	br s						
NH	7.88	br s			19, 21			

a ^{1}H chemical shift values (δ ppm from $SiMe_4$) followed by multiplicity and then the coupling constants (J/Hz). Figures in parentheses indicate the proton coupling with that position. b The correlations with geminal and vicinal protones are removed. c Long range ^{1}H-^{13}C correlations from H to C observed in the HMBC experiment.

Table S4 NMR spectral data of **5** in acetne-d_6

Position	δ_H[a]		J/Hz	^{1}H-^{1}H COSY	NOESY[b]	δ_C		HMBC (C)[c]
1α	1.91	dq	12.6 (1β), 3.6 (2α, 2β, 10)	1β		28.0	(t)	9, 10
1β	1.20	qd	12.6 (1α, 2α, 10), 3.6 (2β)	1α	3, 5			
2α	0.90	m		2β	4α	36.2	(t)	
2β	1.73	ddt	11.4 (2α), 5.4 (3), 3.6 (1α, 1β)	2α, 3	23			
3	1.49	m		2β, 23	1β, 5	33.5	(d)	
4α	0.80	q	12.0 (3, 4β, 5)	4β	2α, 10, 23	42.9	(t)	2, 3, 5, 6, 10, 23
4β	1.87	ddd	12.0 (4α), 5.8 (3), 3.5 (5)	4α	6, 23			
5	2.09	m		10	1β, 3, 24	38.6	(d)	4, 6, 7
6	5.49	d	10.2 (7)	7	4β	133.5	(d)	4, 5, 8, 10
7	6.07	dd	10.2 (6), 3.0	6	14	127.4	(d)	5, 8, 9, 14
8						140.1	(s)	
9						50.6	(s)	
10	1.37	td	12.6 (1α, 5), 3.6 (1α)	5	4α	45.3	(d)	
11						205.2	(s)	
12						72.7	(s)	
13	3.96	d	9.0 (15)	14, 15	16, 24	41.5	(d)	12, 14, 15, 16, 18
14	5.70	d	1.8	13	7	126.9	(d)	7, 9, 12, 13, 15
15	5.37	ddq	16.8 (16), 9.0 (13), 1.8	13, 16	17	129.4	(d)	17
16	5.62	dq	16.8 (15), 6.6 (17)	15, 17	13	129.9	(d)	13, 17
17	1.59	d	6.6 (16)	16	15	17.9	(q)	15, 16
18						204.7	(s)	
19	3.81	d	4.8 (20)	20	21, NH	68.9	(d)	18, 20, 21, 22
20	3.90	m		19, 21		67.0	(d)	
21	1.29	d	6.0 (20)	20	19, NH	20.8	(q)	19, 20
22						170.7	(s)	
23	0.89	d	6.6 (3)	3	2β, 4α, 4β	22.6	(q)	2, 3, 4
24	1.12	s			5, 13	16.6	(q)	8, 9, 10, 11
20-OH	4.02	d	6.0 (20)					
NH	7.86	br s			19, 21			

a ^{1}H chemical shift values (δ ppm from $SiMe_4$) followed by multiplicity and then the coupling constants (J/Hz). Figures in parentheses indicate the proton coupling with that position. b The correlations with geminal and vicinal protones are removed. c Long range ^{1}H-^{13}C correlations from H to C observed in the HMBC experiment.

Figure S1 ^{1}H and ^{13}C NMR spectrum of 1 in acetone-d6

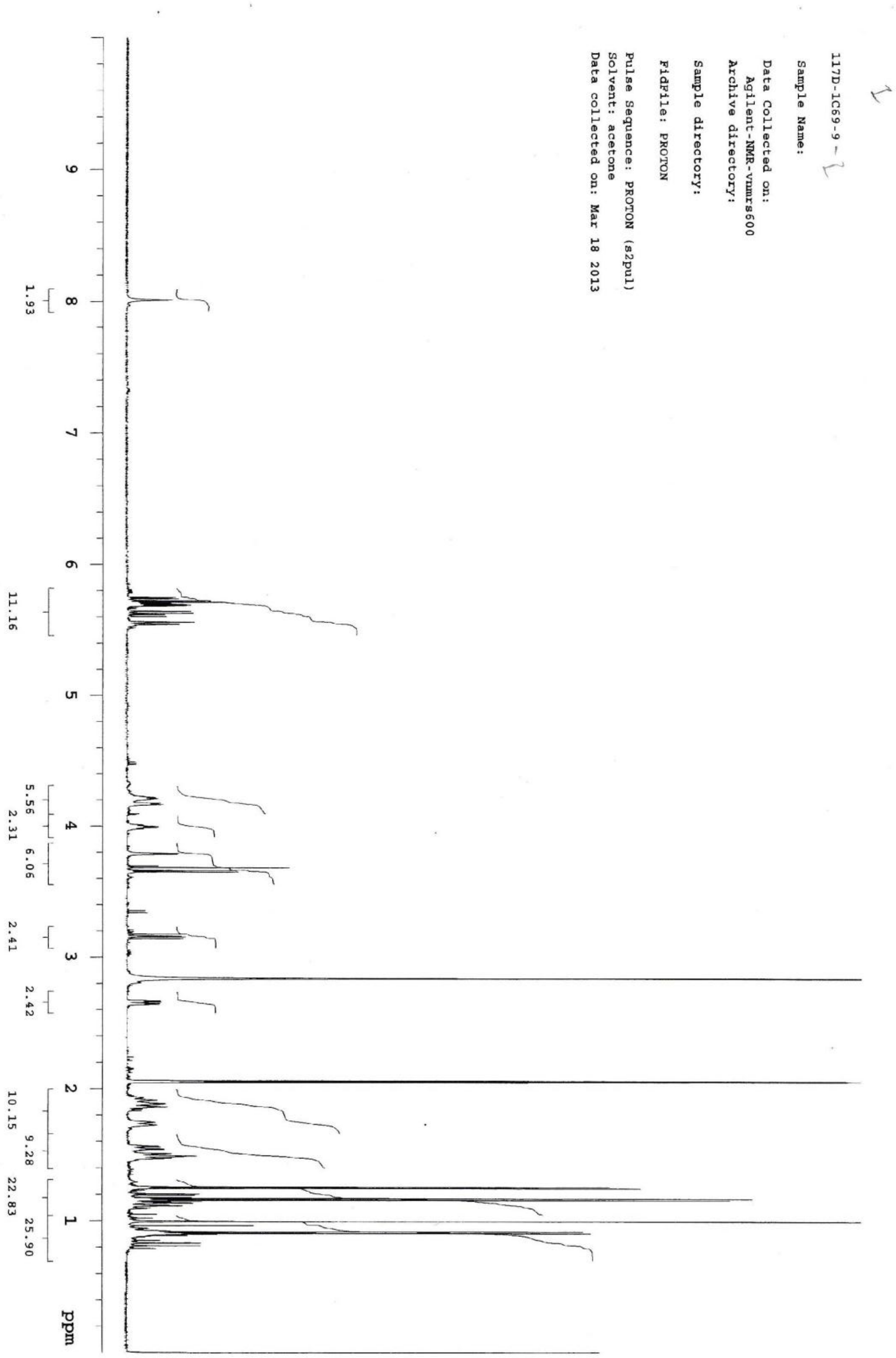

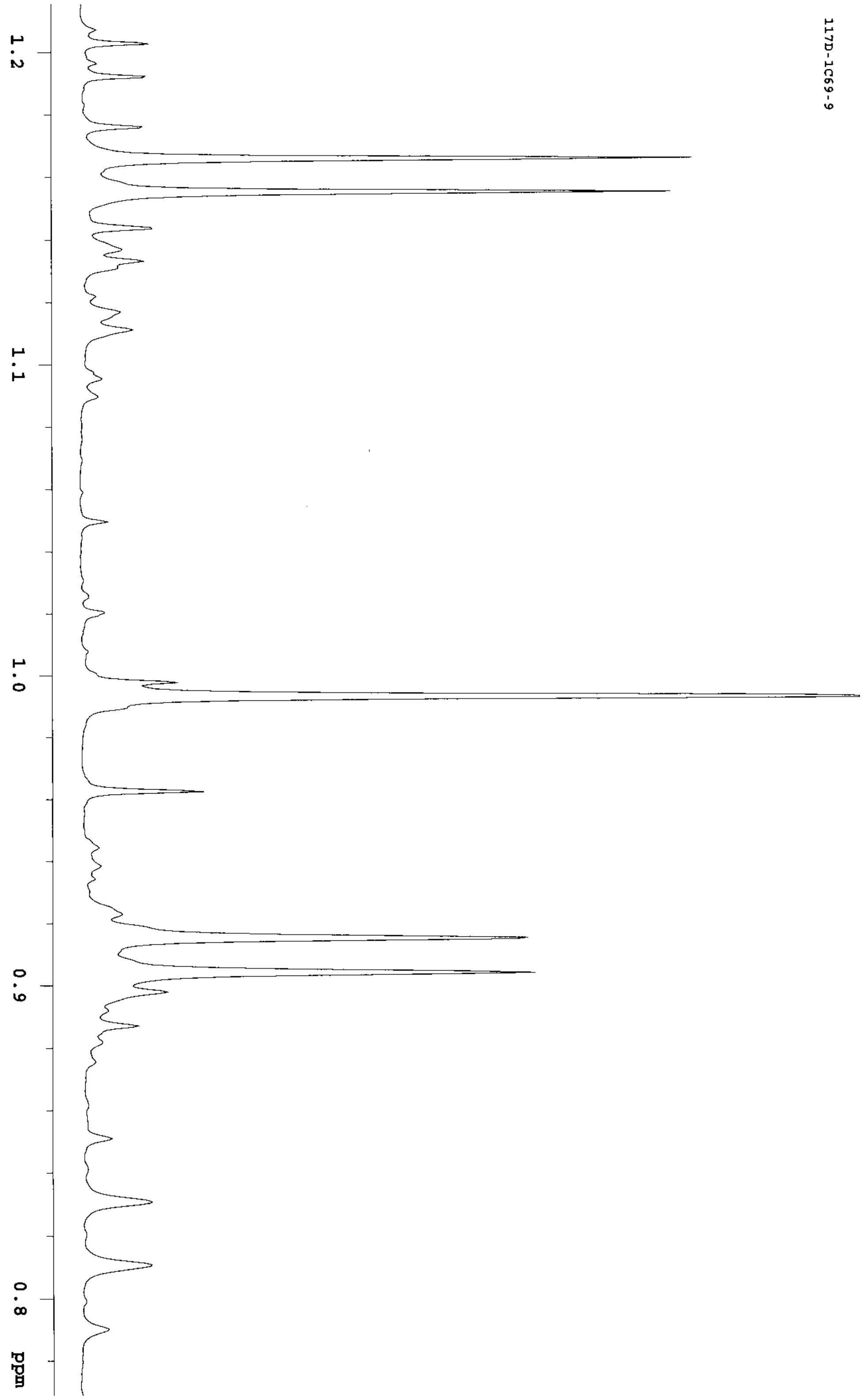
117D-1C69-9
1.2
1.1
1.0
0.9
0.8
ppm

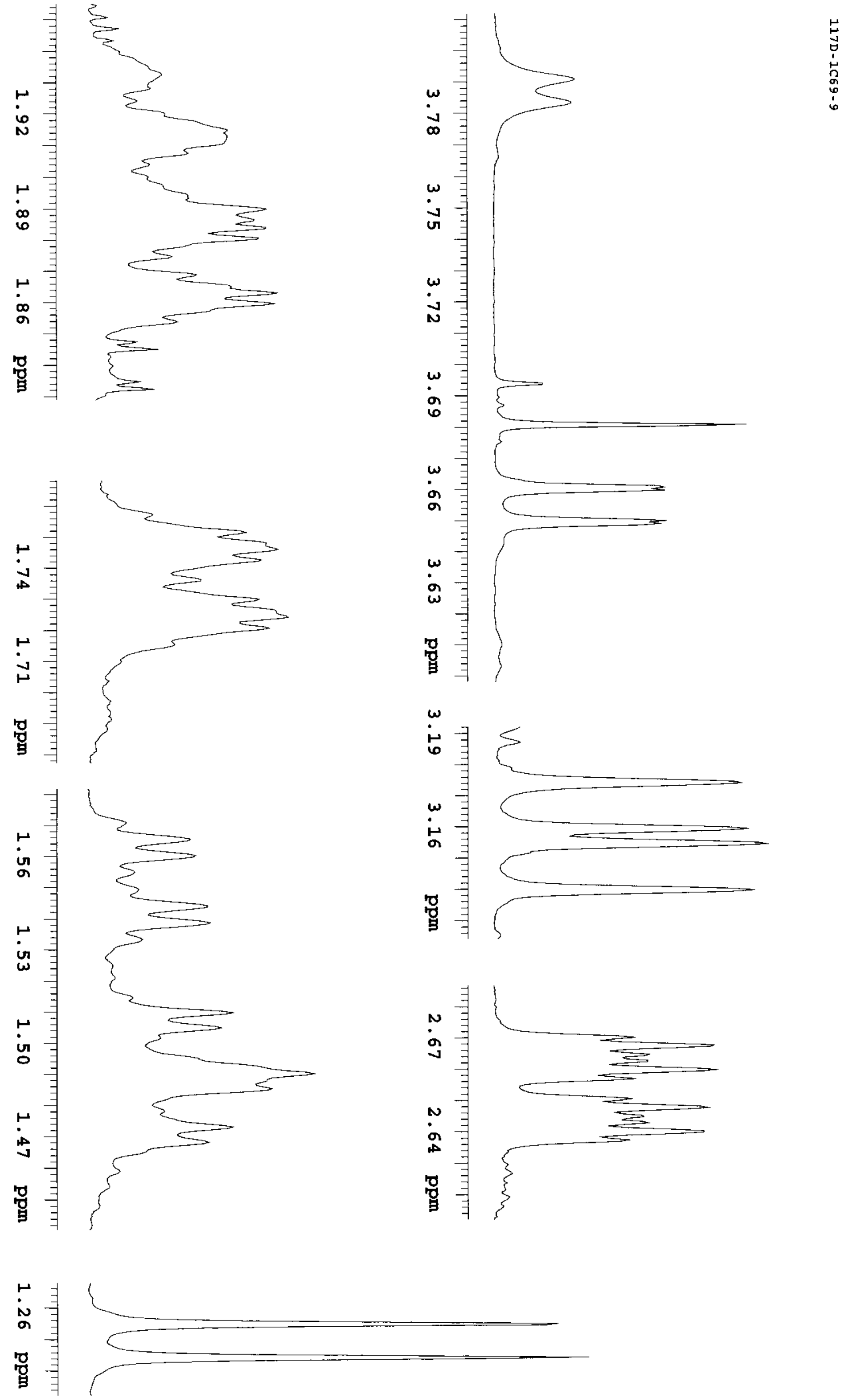
117D-1C69-9
3.78 3.75 3.72 3.69 3.66 3.63 ppm
3.19 3.16 ppm
2.67 2.64 ppm
1.92 1.89 1.86 ppm
1.74 1.71 ppm
1.56 1.53 1.50 1.47 ppm
1.26 ppm

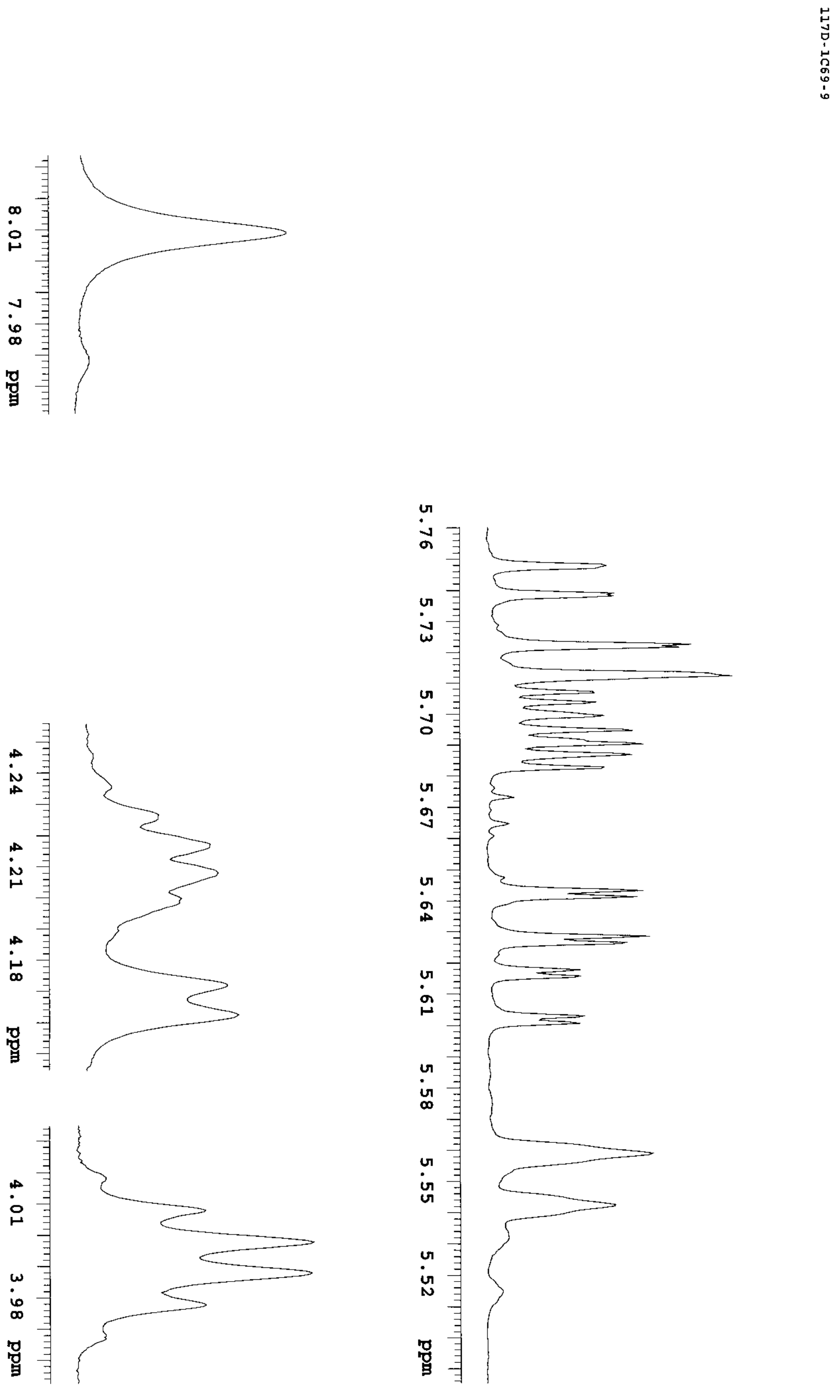
117D-1C69-9
8.01
7.98
ppm
5.76
5.73
5.70
5.67
5.64
5.61
5.58
5.55
5.52
ppm
4.24
4.21
4.18
ppm
4.01
3.98
ppm

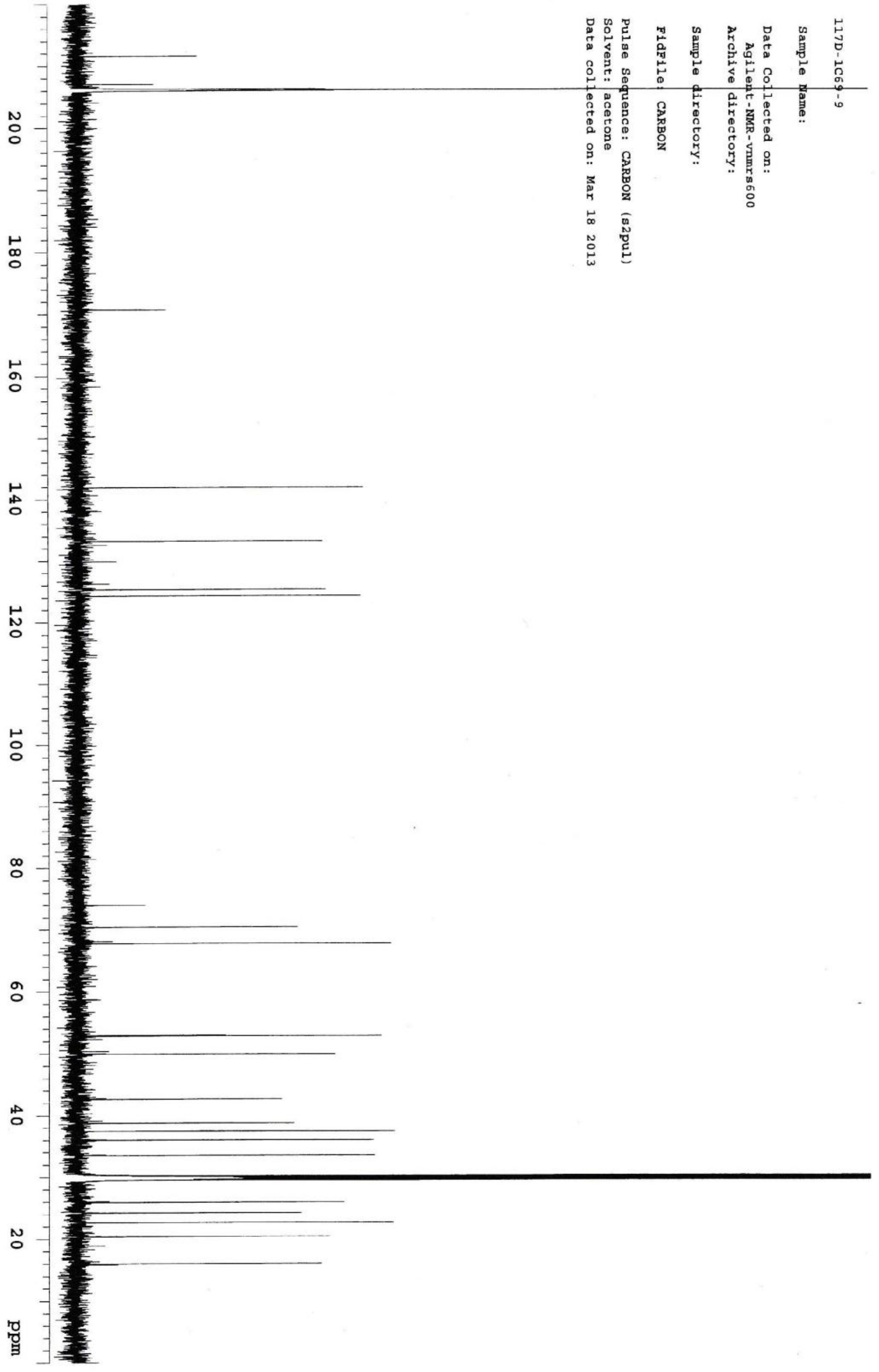
117D-1C69-9
Sample Name:
Data Collected on:
Agilent-NMR-vnmrs600
Archive directory:
Sample directory:
FidFile: CARBON
Pulse Sequence: CARBON (s2pul)
Solvent: acetone
Data collected on: Mar 18 2013
200
180
160
140
120
100
80
60
40
20
ppm

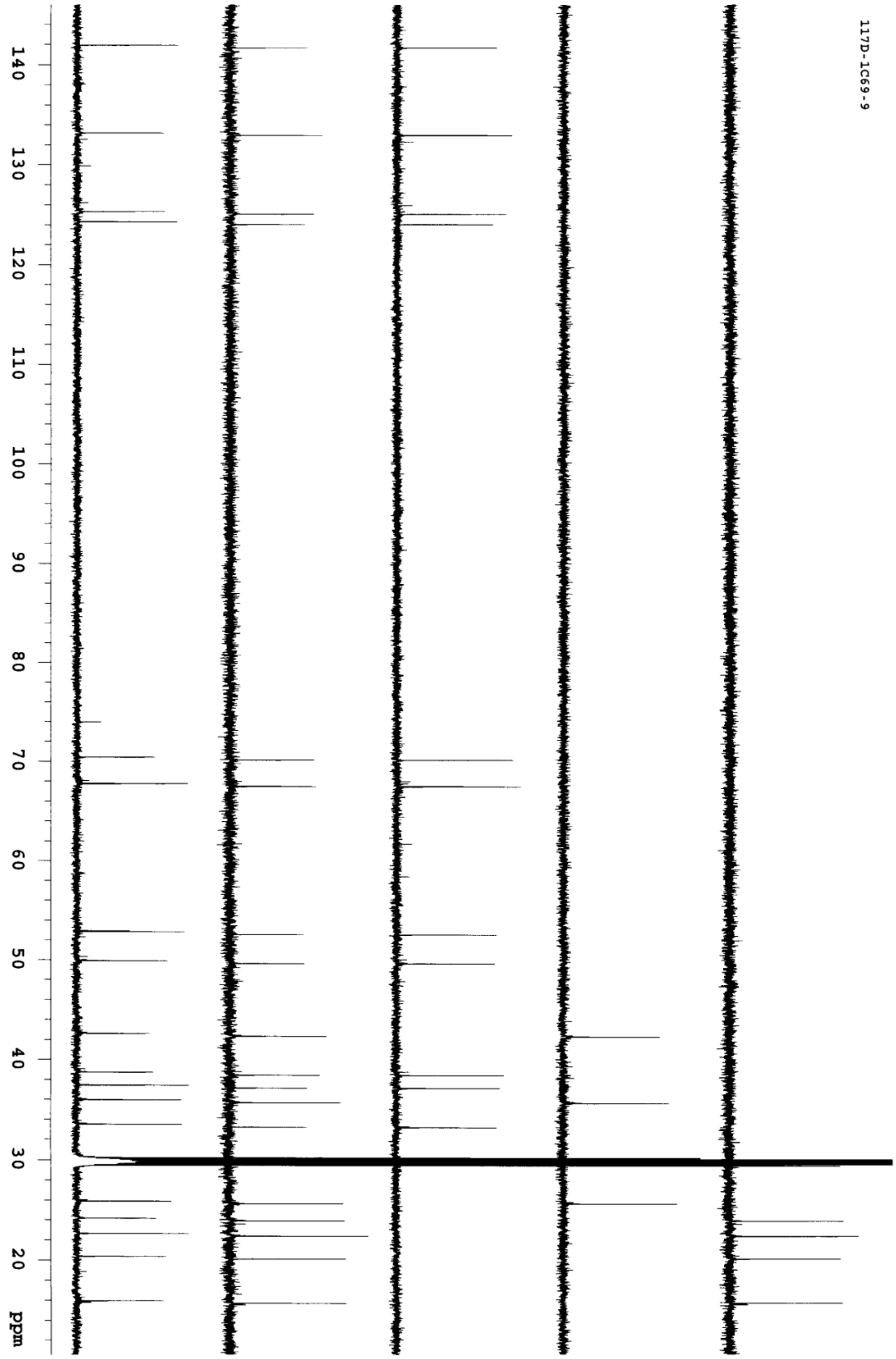
117D-1C69-9
140
130
120
110
100
90
80
70
60
50
40
30
20
ppm

Figure S2 ^{1}H-^{1}H COSY of 1

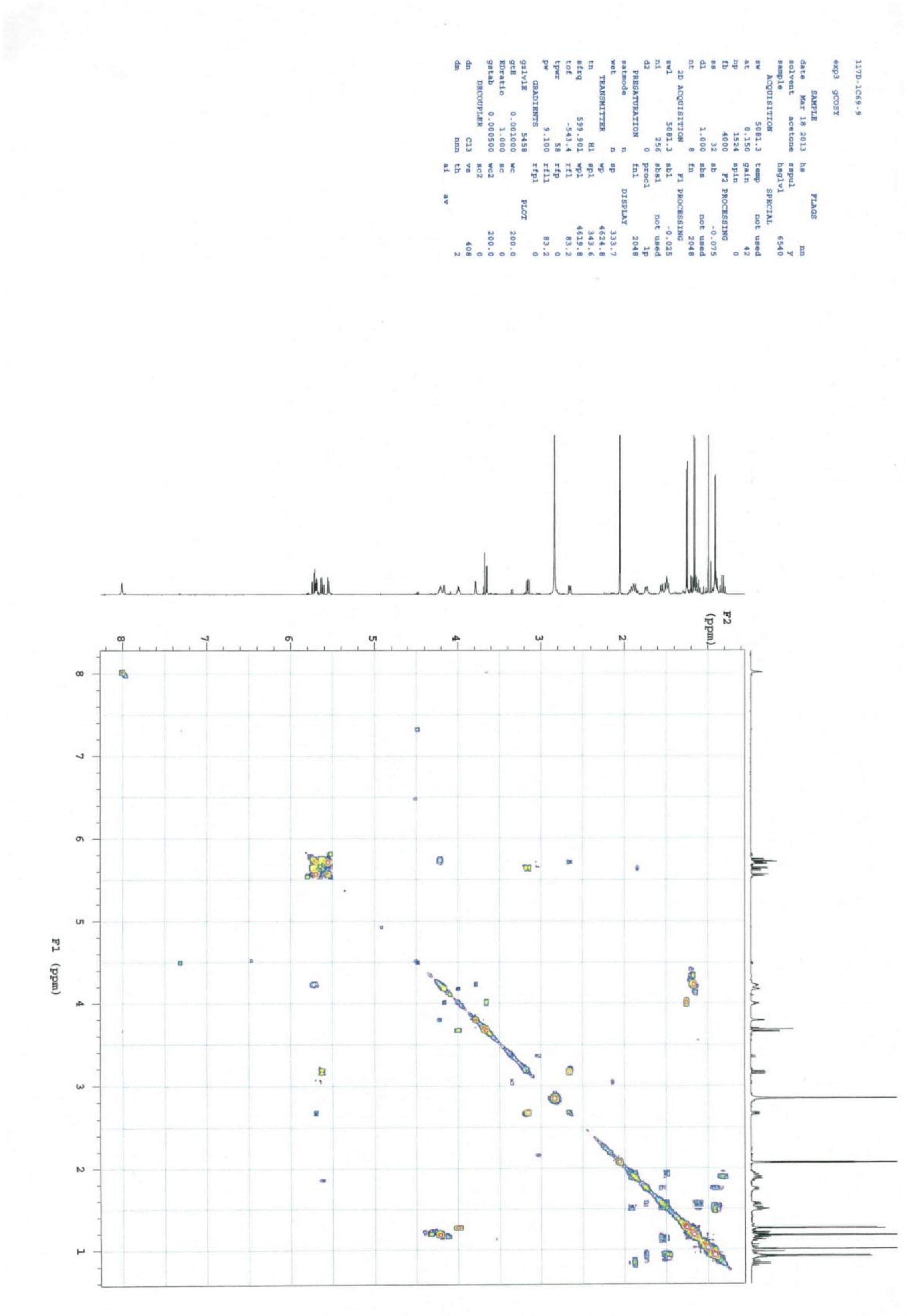

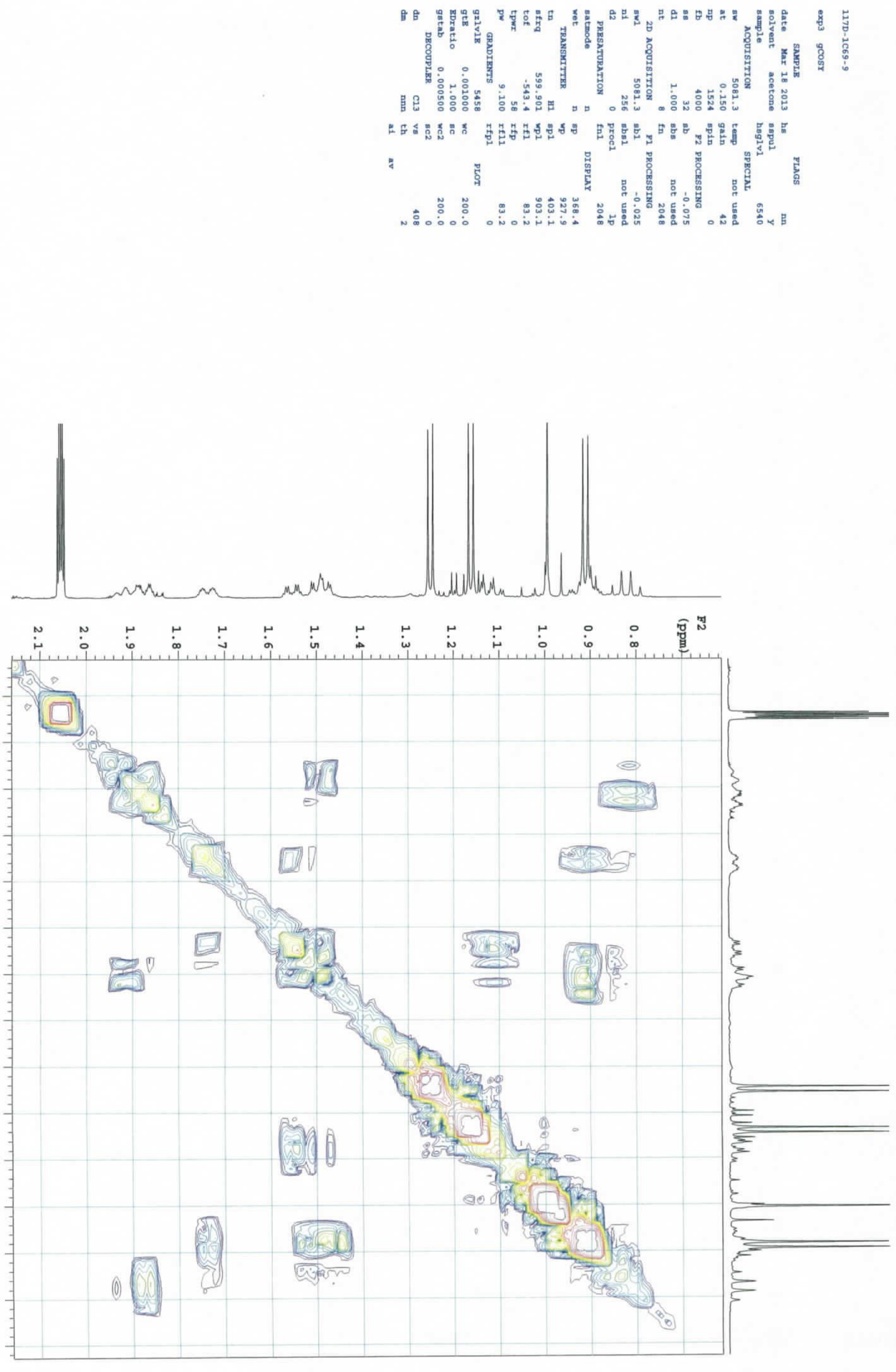
117D-1C69-9
exp3 gCOSY
SAMPLE
date Mar 18 2013
solvent acetone
sample
FLAGS
hs nn
sspul y
hsglvl 6540
ACQUISITION
sw 5081.3
at 0.150
np 1524
fb 4000
ss 32
d1 1.000
nt 8
SPECIAL
temp not used
gain 42
spin 0
F2 PROCESSING
sb -0.075
sbs not used
fn 2048
2D ACQUISITION
sw1 5081.3
ni 256
d2 0
F1 PROCESSING
sb1 -0.025
sbs1 not used
proc1 lp
fn1 2048
PRESATURATION
satmode n
wet n
DISPLAY
sp 368.4
wp 927.9
sp1 403.1
wp1 903.1
rfl 83.2
rfp 0
rfl1 83.2
rfp1 0
TRANSMITTER
tn H1
sfrq 599.901
tof -543.4
tpwr 58
pw 9.100
GRADIENTS
gzlvlE 5458
gtE 0.001000
EDratio 1.000
gstab 0.000500
PLOT
wc 200.0
sc 0
wc2 200.0
sc2 0
DECOUPLER
dn C13
dm nnn
vs 408
th 2
ai av
F2 (ppm)
F1 (ppm)

Figure S3 NOESY of 1

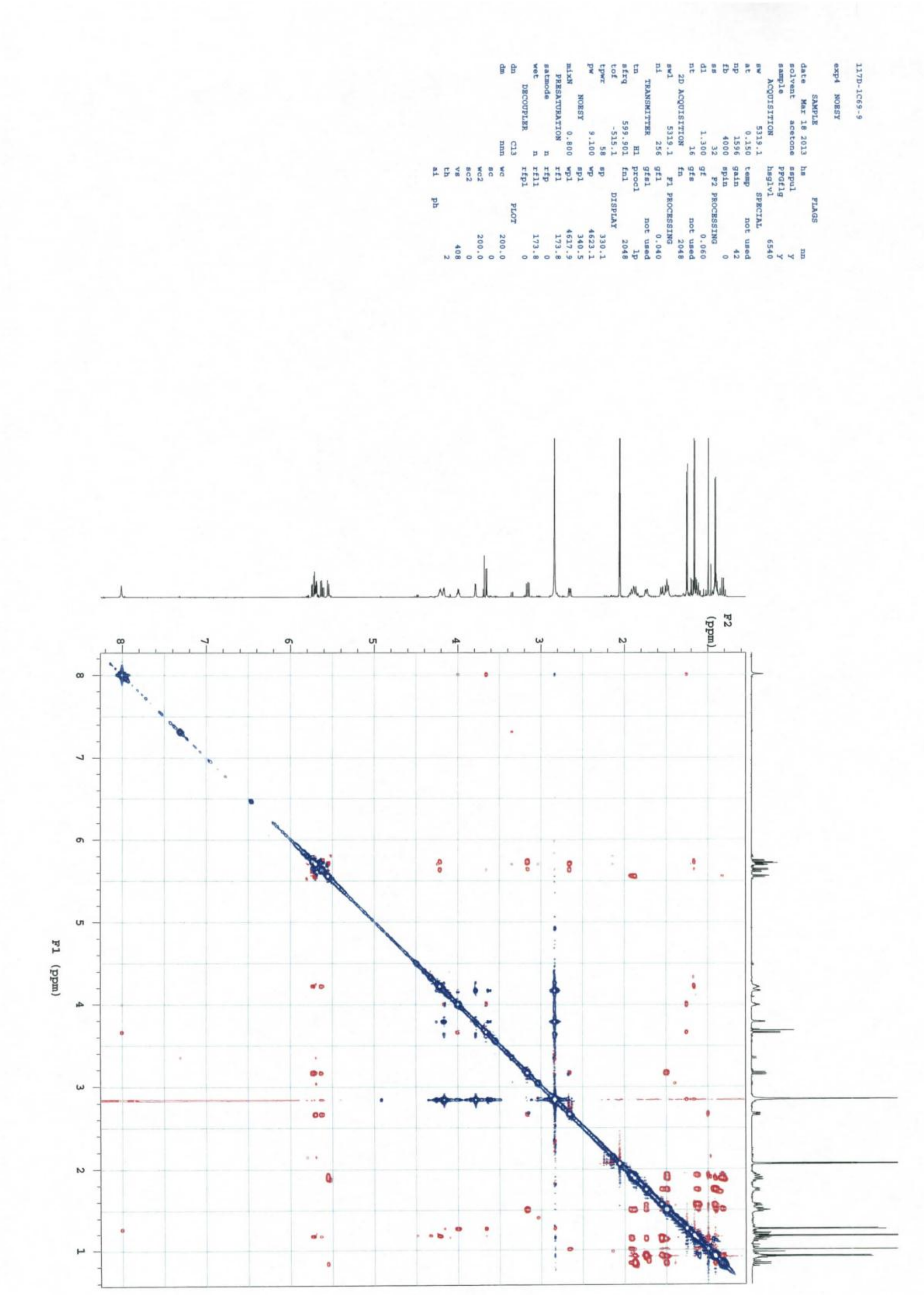

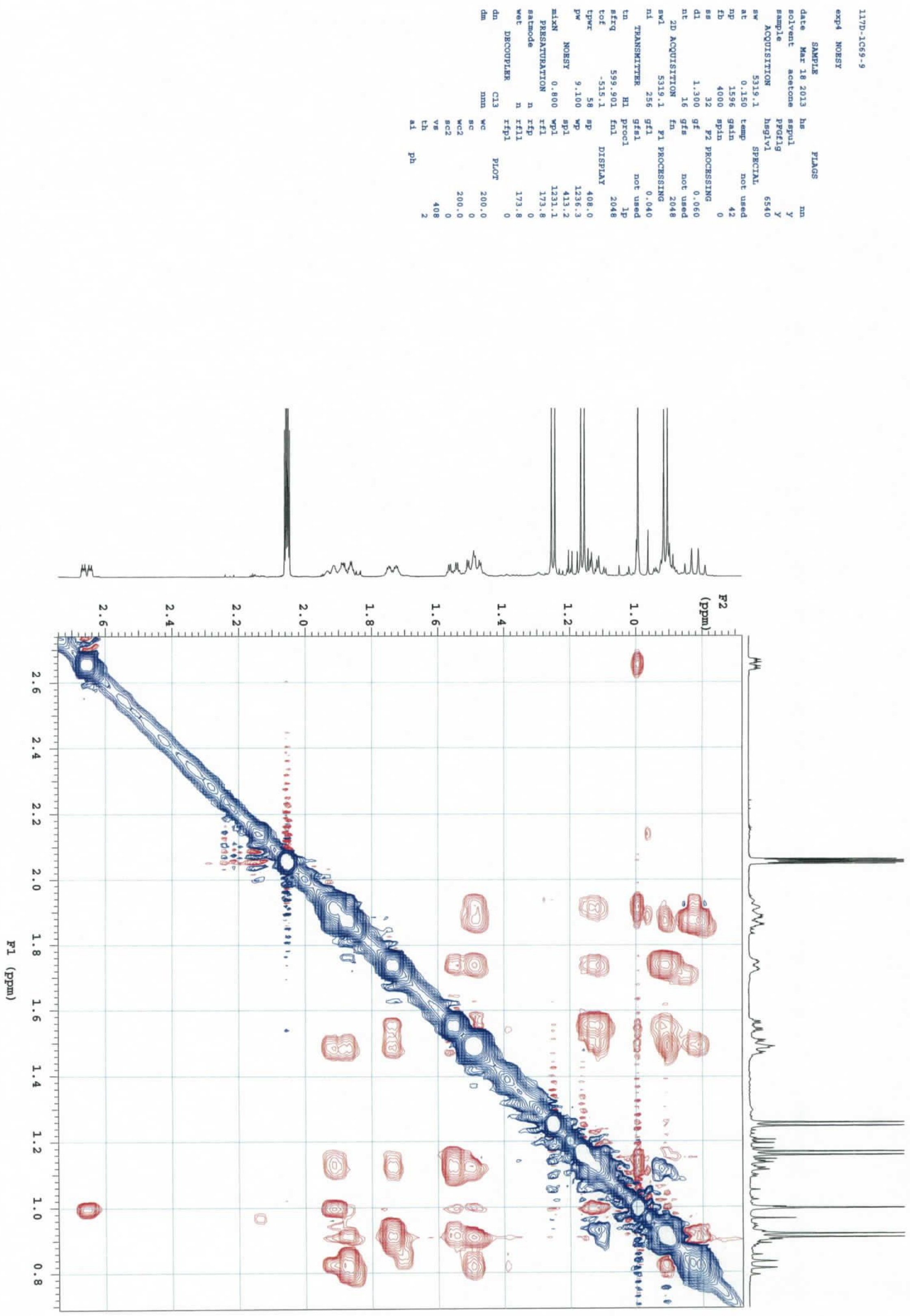
117D-1C69-9
exp4 NOESY
SAMPLE
date Mar 18 2013
solvent acetone
sample
ACQUISITION
sw 5319.1
at 0.150
np 1596
fb 4000
ss 32
d1 1.300
nt 16
2D ACQUISITION
sw1 5319.1
ni 256
TRANSMITTER
tn H1
sfrq 599.901
tof -515.1
tpwr 58
pw 9.100
NOESY
mixN 0.800
PRESATURATION
satmode n
wet n
DECOUPLER
dn C13
dm nnn
FLAGS
hs nn
sspul y
PFGflg y
hsglvl 6540
SPECIAL
temp not used
gain 42
spin 0
F2 PROCESSING
gf 0.060
gfs not used
fn 2048
F1 PROCESSING
gf1 0.040
gfs1 not used
procl lp
fn1 2048
DISPLAY
sp 408.0
wp 1236.3
sp1 413.2
wp1 1231.1
rfl 173.8
rfp 0
rfl1 173.8
rfp1 0
PLOT
wc 200.0
sc 0
wc2 200.0
sc2 0
vs 408
th 2
ai ph
F2 (ppm)
F1 (ppm)

Figure S4 HMQC of 1

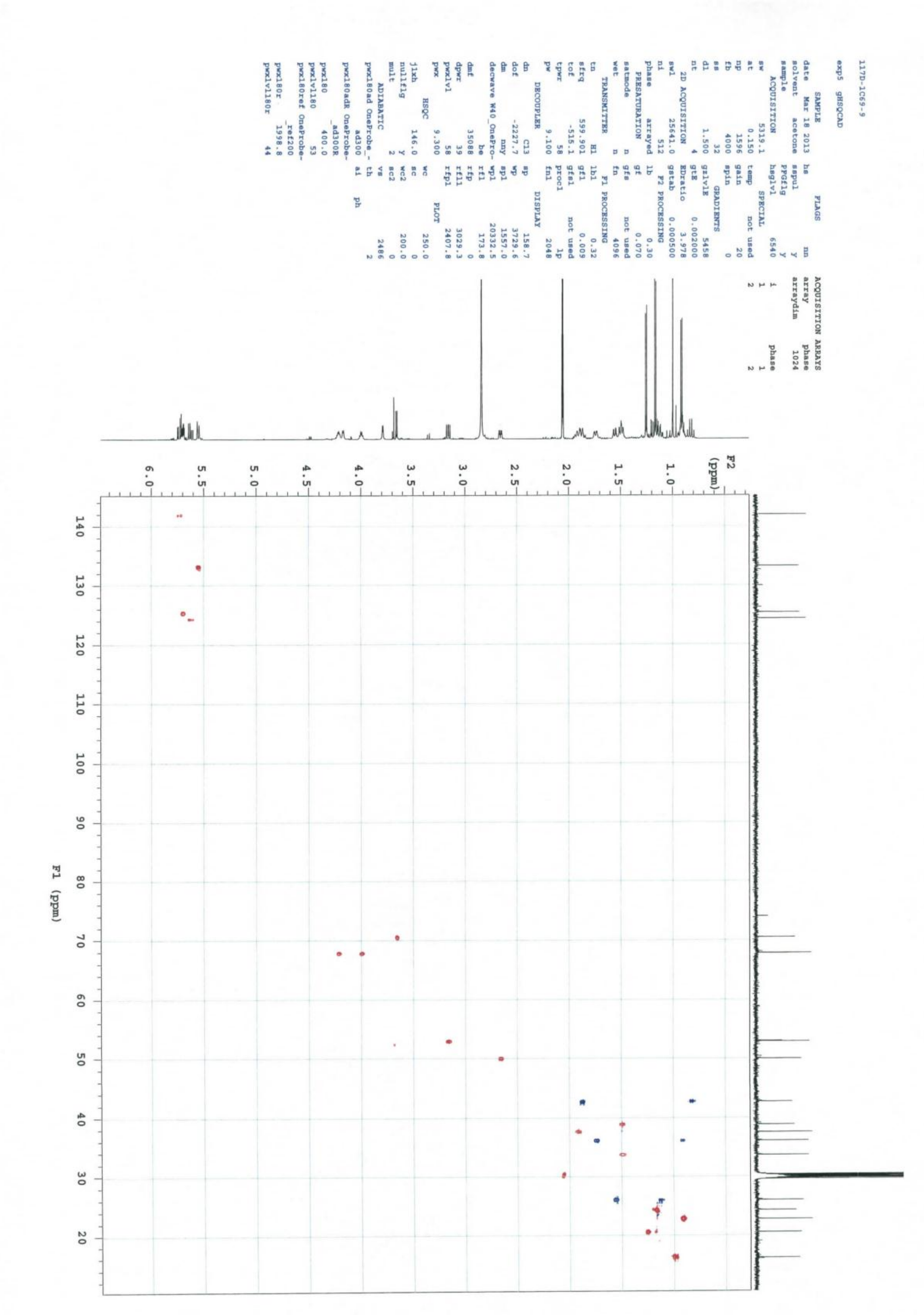

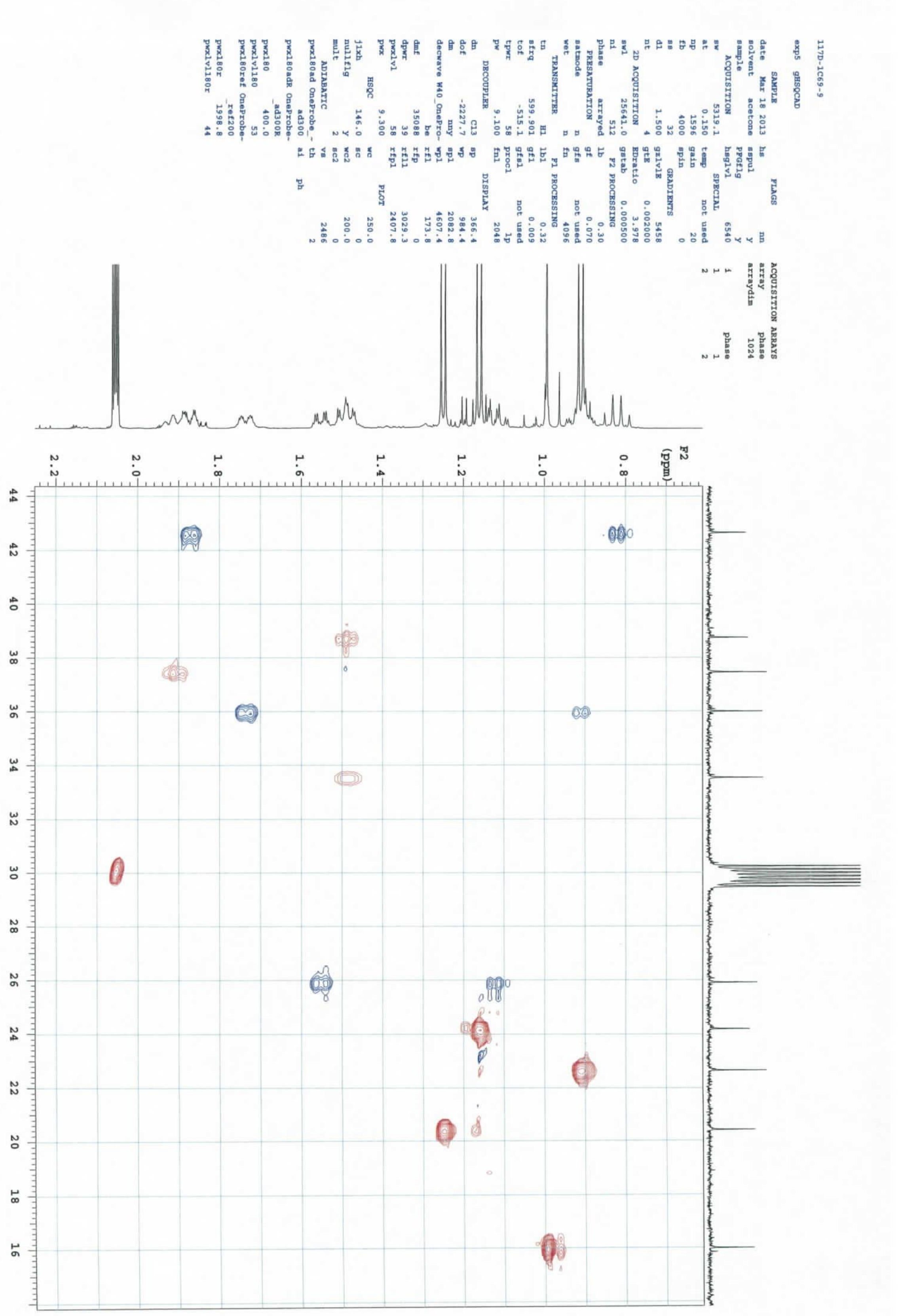
117D-1C69-9
exp5 gHSQCAD
F2 (ppm)
F1 (ppm)

Figure S5 HMBC of 1

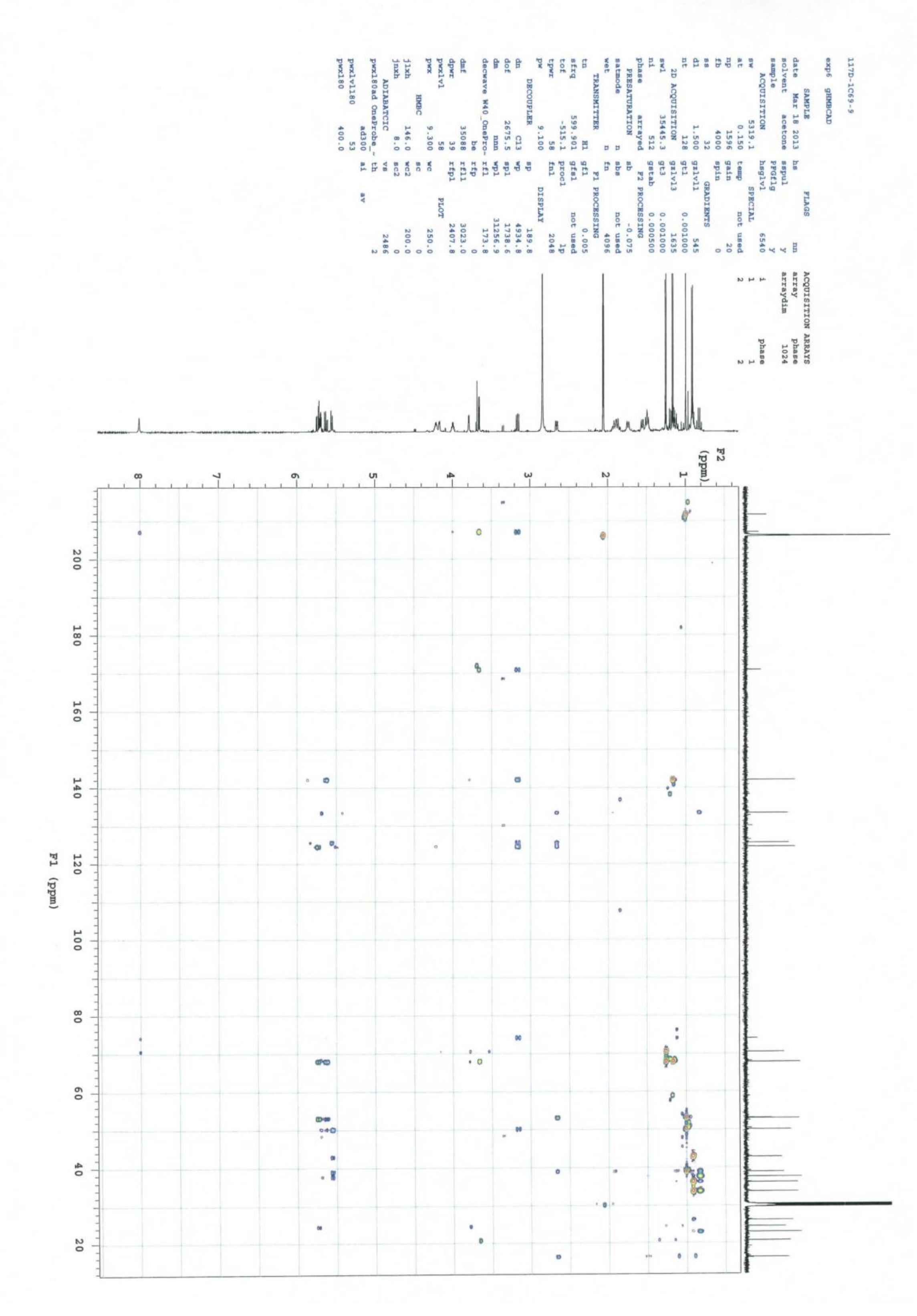

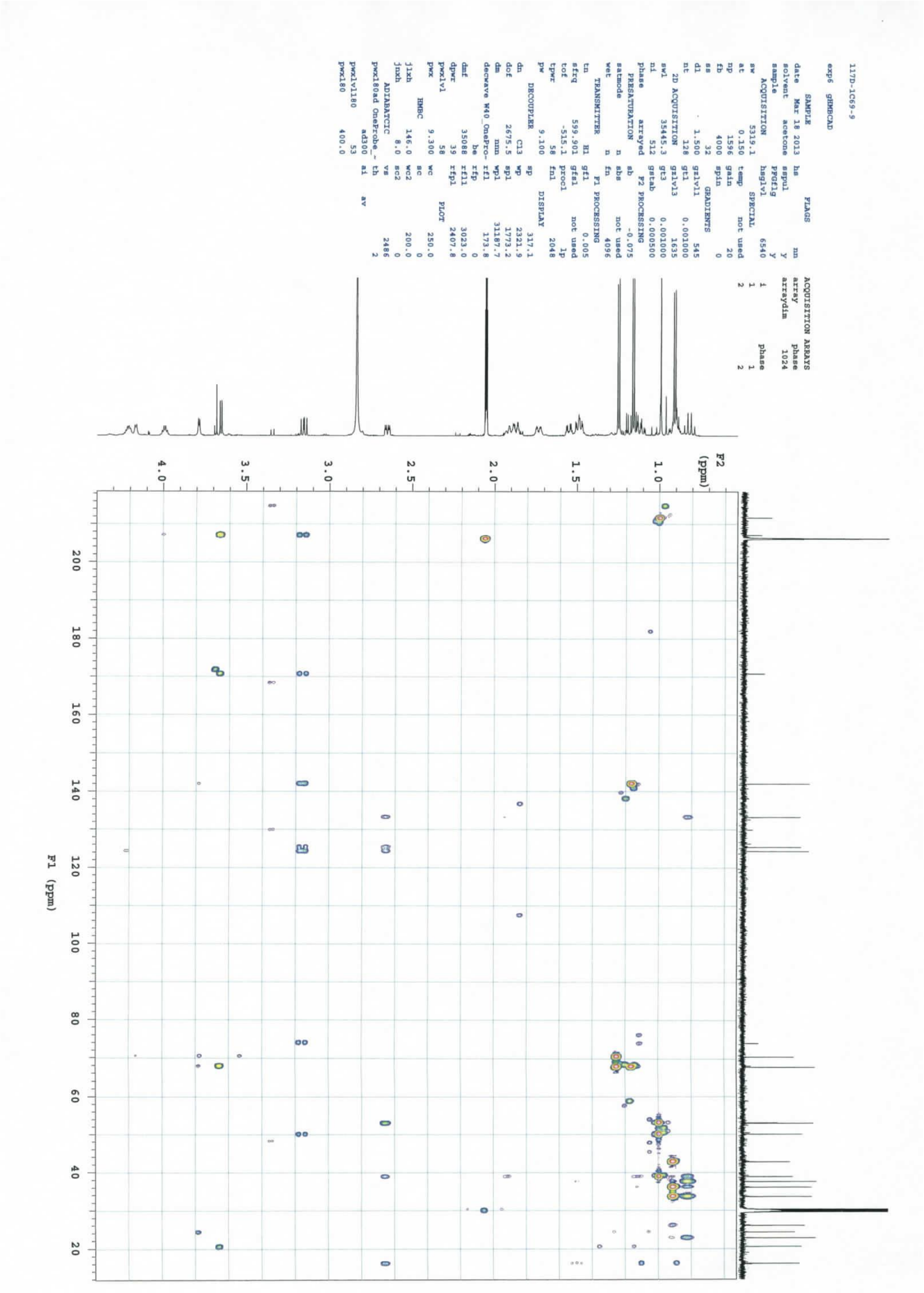
117D-1C69-9
exp6 gHMBCAD
F2 (ppm)
F1 (ppm)

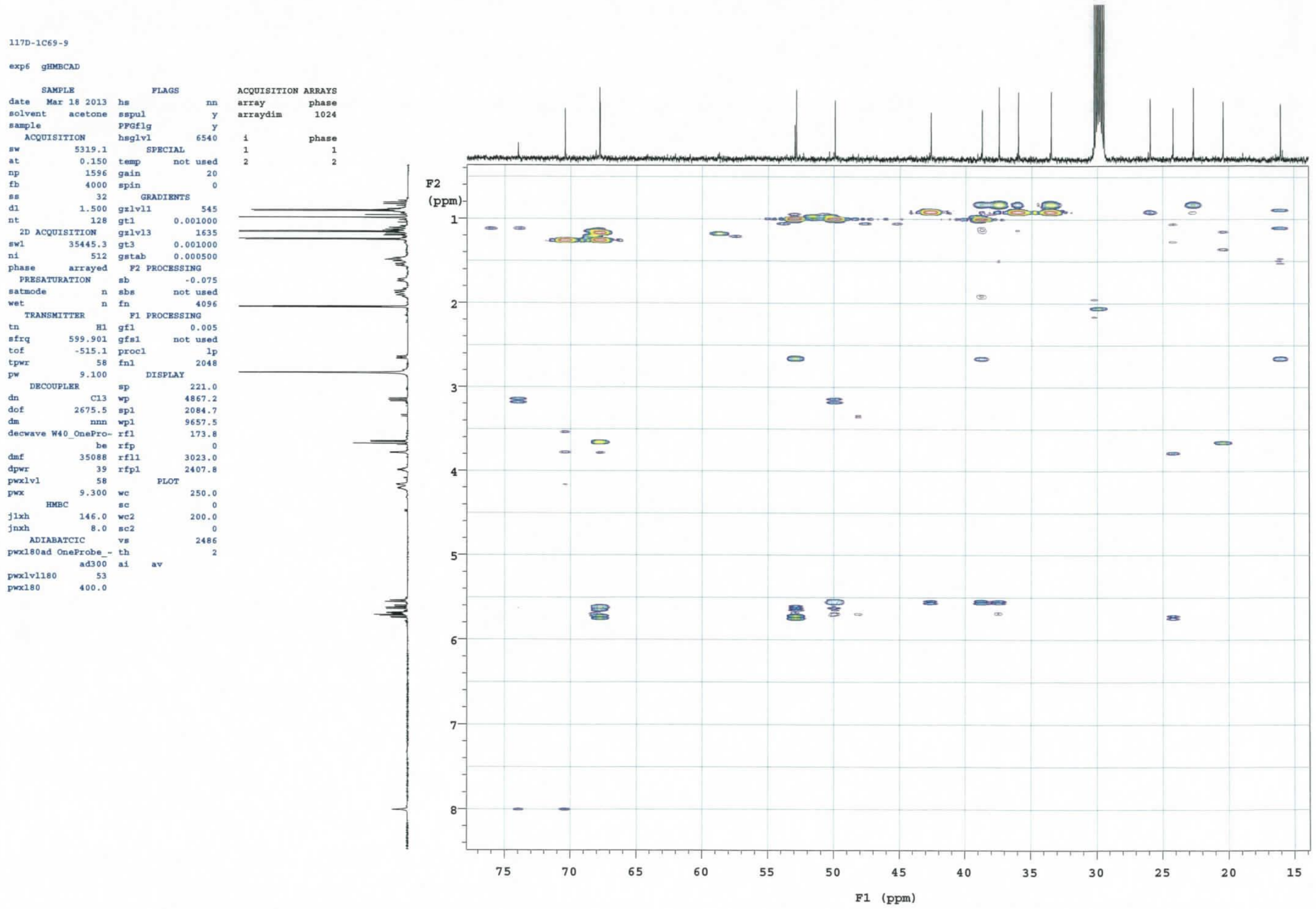
117D-1C69-9
exp6 gHMBCAD
SAMPLE
date Mar 18 2013
solvent acetone
sample
ACQUISITION
sw 5319.1
at 0.150
np 1596
fb 4000
ss 32
d1 1.500
nt 128
2D ACQUISITION
sw1 35445.3
ni 512
phase arrayed
PRESATURATION
satmode n
wet n
TRANSMITTER
tn H1
sfrq 599.901
tof -515.1
tpwr 58
pw 9.100
DECOUPLER
dn C13
dof 2675.5
dm nnn
decwave W40_OnePro-be
dmf 35088
dpwr 39
pwxlvl 58
pwx 9.300
HMBC
j1xh 146.0
jnxh 8.0
ADIABATCIC
pwx180ad OneProbe_-ad300
pwxlvl180 53
pwx180 400.0
FLAGS
hs nn
sspul y
PFGflg y
hsglvl 6540
SPECIAL
temp not used
gain 20
spin 0
GRADIENTS
gzlvl1 545
gt1 0.001000
gzlvl3 1635
gt3 0.001000
gstab 0.000500
F2 PROCESSING
sb -0.075
sbs not used
fn 4096
F1 PROCESSING
gf1 0.005
gfs1 not used
proc1 lp
fn1 2048
DISPLAY
sp 221.0
wp 4867.2
sp1 2084.7
wp1 9657.5
rfl 173.8
rfp 0
rfl1 3023.0
rfp1 2407.8
PLOT
wc 250.0
sc 0
wc2 200.0
sc2 0
vs 2486
th 2
ai av
ACQUISITION ARRAYS
array phase
arraydim 1024
i phase
1 1
2 2
F2 (ppm)
F1 (ppm)

```
117D-1C69-9

exp6  gHMBCAD

      SAMPLE              FLAGS          ACQUISITION ARRAYS
date    Mar 18 2013  hs              nn   array          phase
solvent     acetone  sspul            y   arraydim        1024
sample               PFGflg           y
    ACQUISITION      hsglvl        6540     i             phase
sw           5319.1        SPECIAL          1                 1
at            0.150  temp      not used     2                 2
np             1596  gain            20
fb             4000  spin             0
ss               32       GRADIENTS
d1            1.500  gzlvl1         545
nt              128  gt1       0.001000
  2D ACQUISITION     gzlvl3        1635
sw1         35445.3  gt3       0.001000
ni              512  gstab     0.000500
phase       arrayed     F2 PROCESSING
  PRESATURATION      sb          -0.075
satmode           n  sbs       not used
wet               n  fn            4096
    TRANSMITTER         F1 PROCESSING
tn               H1  gf1          0.005
sfrq        599.901  gfs1      not used
tof          -515.1  proc1           lp
tpwr             58  fn1           2048
pw            9.100        DISPLAY
     DECOUPLER       sp          3135.1
dn              C13  wp           509.1
dof          2675.5  sp1         1773.2
dm              nnn  wp1        20353.3
decwave W40_OnePro-  rfl          173.8
                 be  rfp              0
dmf           35088  rfl1        3023.0
dpwr             39  rfp1        2407.8
pwxlvl           58         PLOT
pwx           9.300  wc           250.0
       HMBC          sc               0
j1xh          146.0  wc2          200.0
jnxh            8.0  sc2              0
     ADIABATCIC      vs            2486
pwx180ad OneProbe_-  th               2
              ad300  ai    av
pwxlvl180        53
pwx180        400.0
```

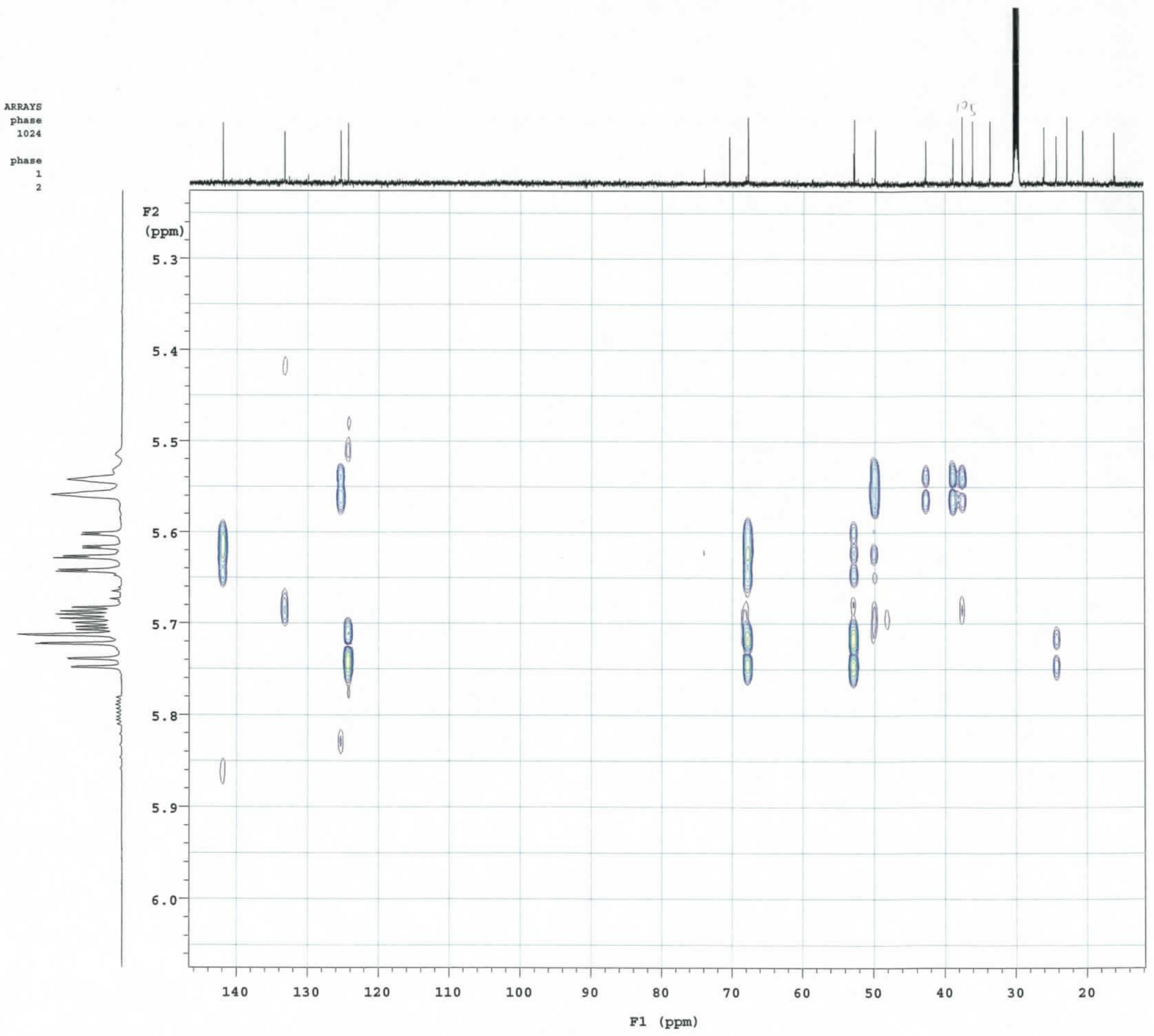

Figure S6　FABMS of 1

[Elemental Composition]　　　　　　　　　　　　　　　　　　Page: 1

Data : 1607230　　　　　　　　　　　Date : 04-Aug-2016 17:33

Sample: 117D-1 C176-11-VI

Note : Matrix; NBA

Inlet : Direct　　　　　　　　　　　　Ion Mode : FAB+

RT : 8.07 min　　　　　　　　　　　　Scan#: (40,45)

Elements : C 30/20, H 40/30, N 5/0, O 10/0

Mass Tolerance : 20ppm, 1mmu if m/z > 50

Unsaturation (U.S.) : -1.0 - 30.0

Observed m/z	Int%	Err[ppm / mmu]	U.S.	Composition
416.2436	100.0	-0.3 / -0.1	8.5	C 24 H 34 N O 5

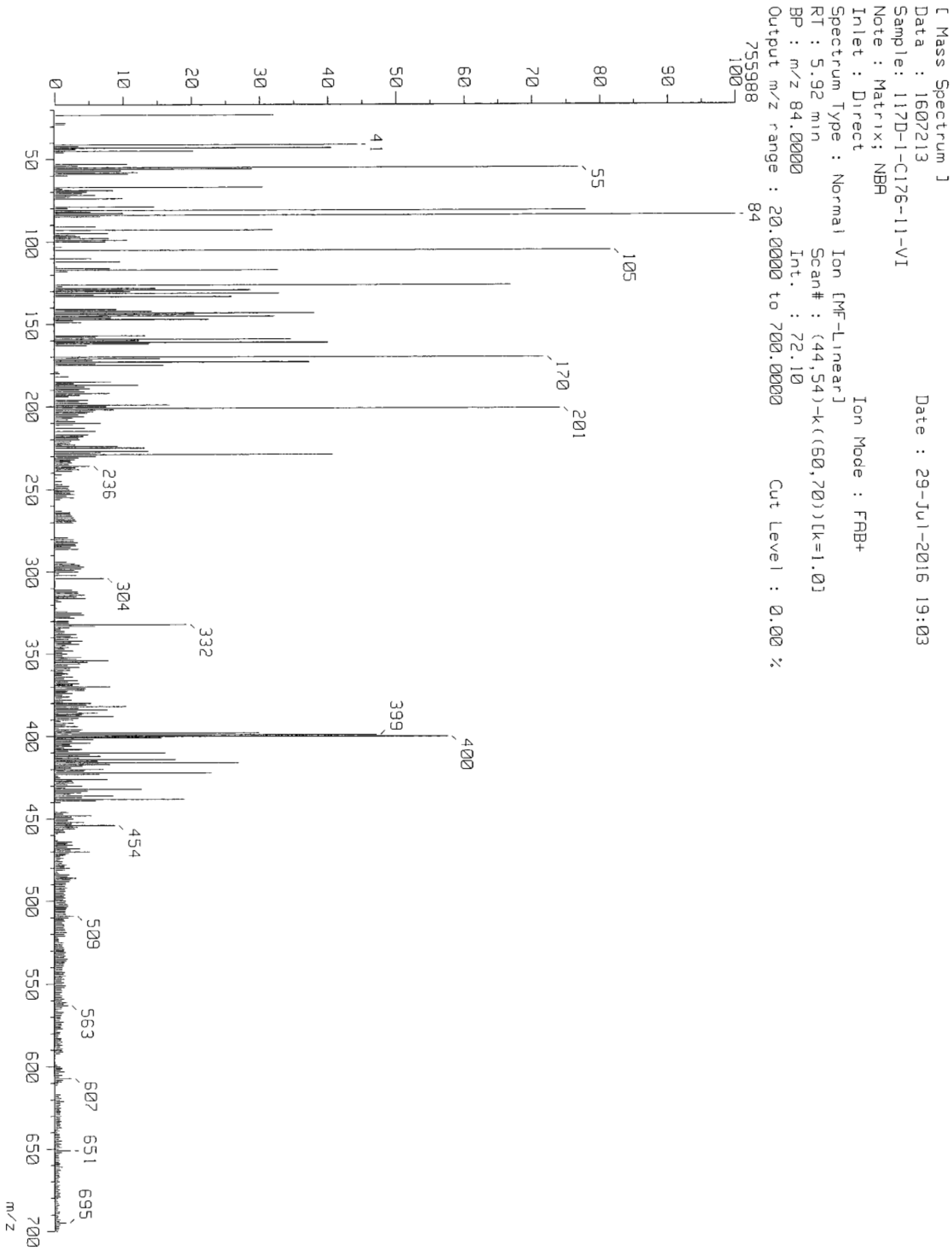
[Mass Spectrum]
Data : 1607213
Sample: 117D-1-C176-11-VI
Note : Matrix; NBA
Inlet : Direct
Spectrum Type : Normal Ion [MF-Linear]
RT : 5.92 min
BP : m/z 84.0000
Output m/z range : 20.0000 to 700.0000
Date : 29-Jul-2016 19:03
Ion Mode : FAB+
Scan# : (44,54)-k((60,70))[k=1.0]
Int. : 72.10
Cut Level : 0.00 %
755988
100
90
80
70
60
50
40
30
20
10
0
41
55
84
105
170
201
236
304
332
399
400
454
509
563
607
651
695
50
100
150
200
250
300
350
400
450
500
550
600
650
700
m/z
