# Supplementary material for: Altercrasins A–E, Decalin Derivatives, from a Sea-Urchin-Derived Alternaria sp.: Isolation and Structural Analysis Including Stereochemistry
Source: Mar Drugs. 2019 Apr 11;17(4):218. doi: 10.3390/md17040218 (PMC6521173; doi:10.3390/md17040218)

Figure S7 ^{1}H and ^{13}C NMR spectra in acetone-d_6 of 2

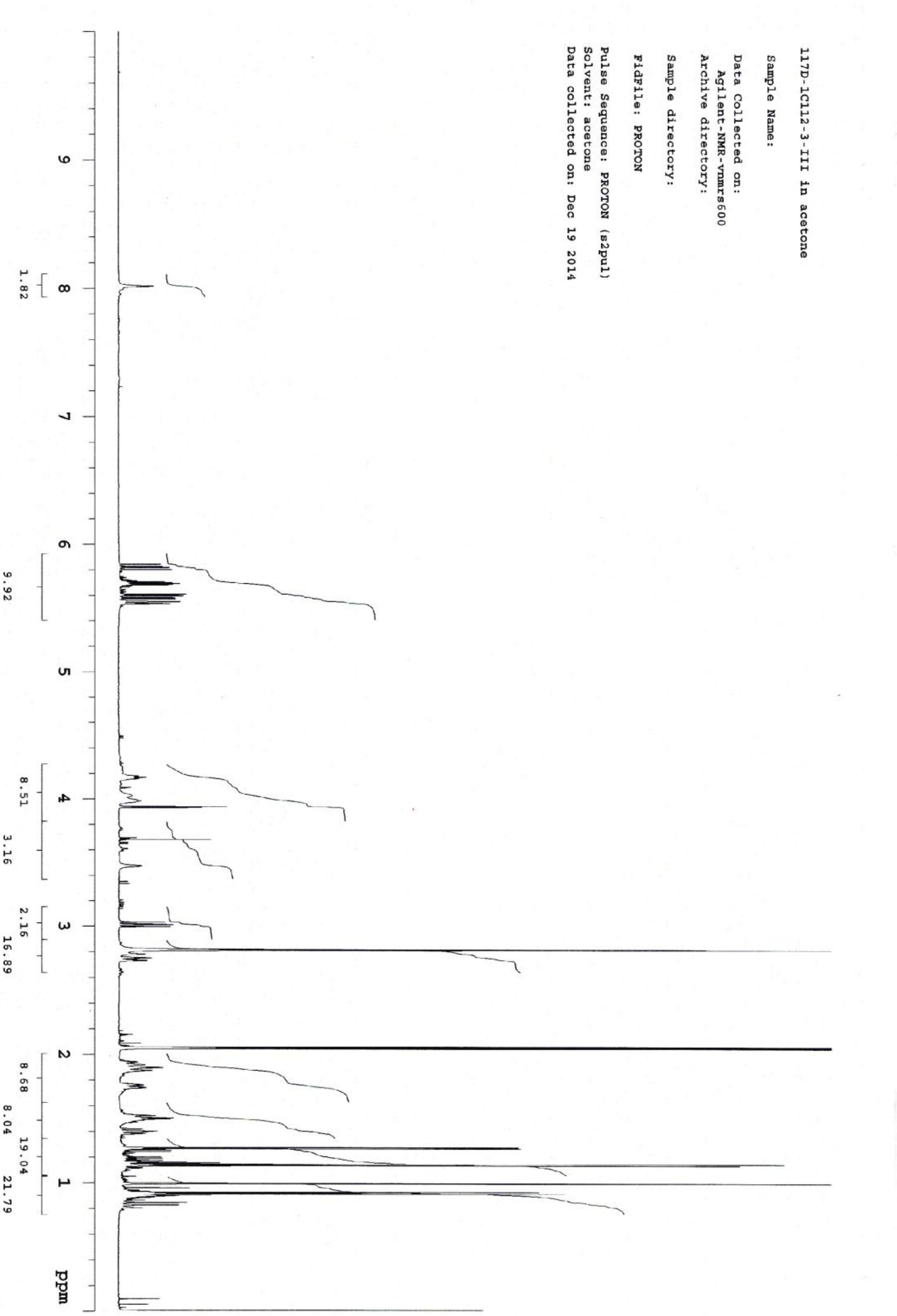

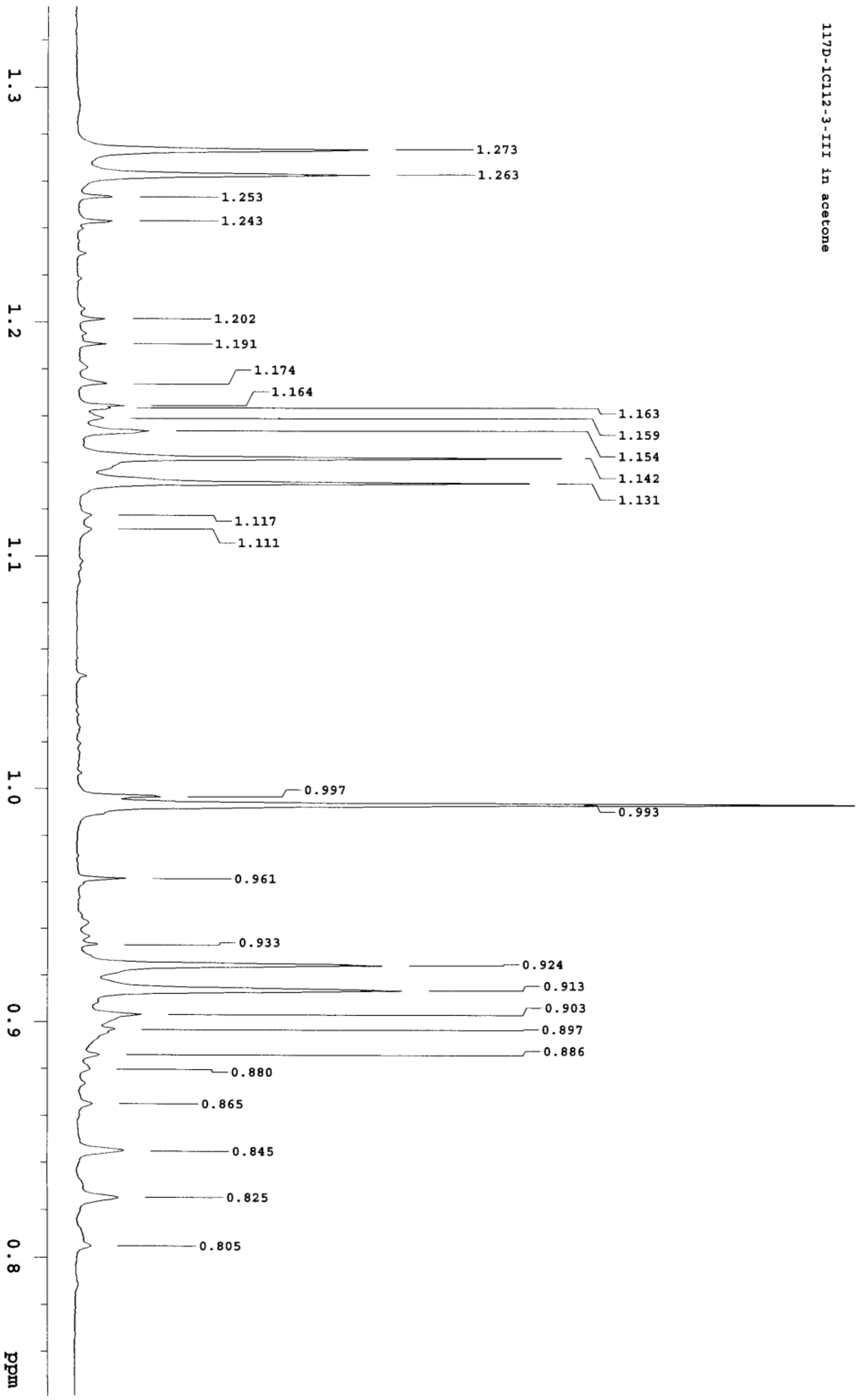
117D-1C112-3-III in acetone
1.273
1.263
1.253
1.243
1.202
1.191
1.174
1.164
1.163
1.159
1.154
1.142
1.131
1.117
1.111
0.997
0.993
0.961
0.933
0.924
0.913
0.903
0.897
0.886
0.880
0.865
0.845
0.825
0.805
1.3
1.2
1.1
1.0
0.9
0.8
ppm

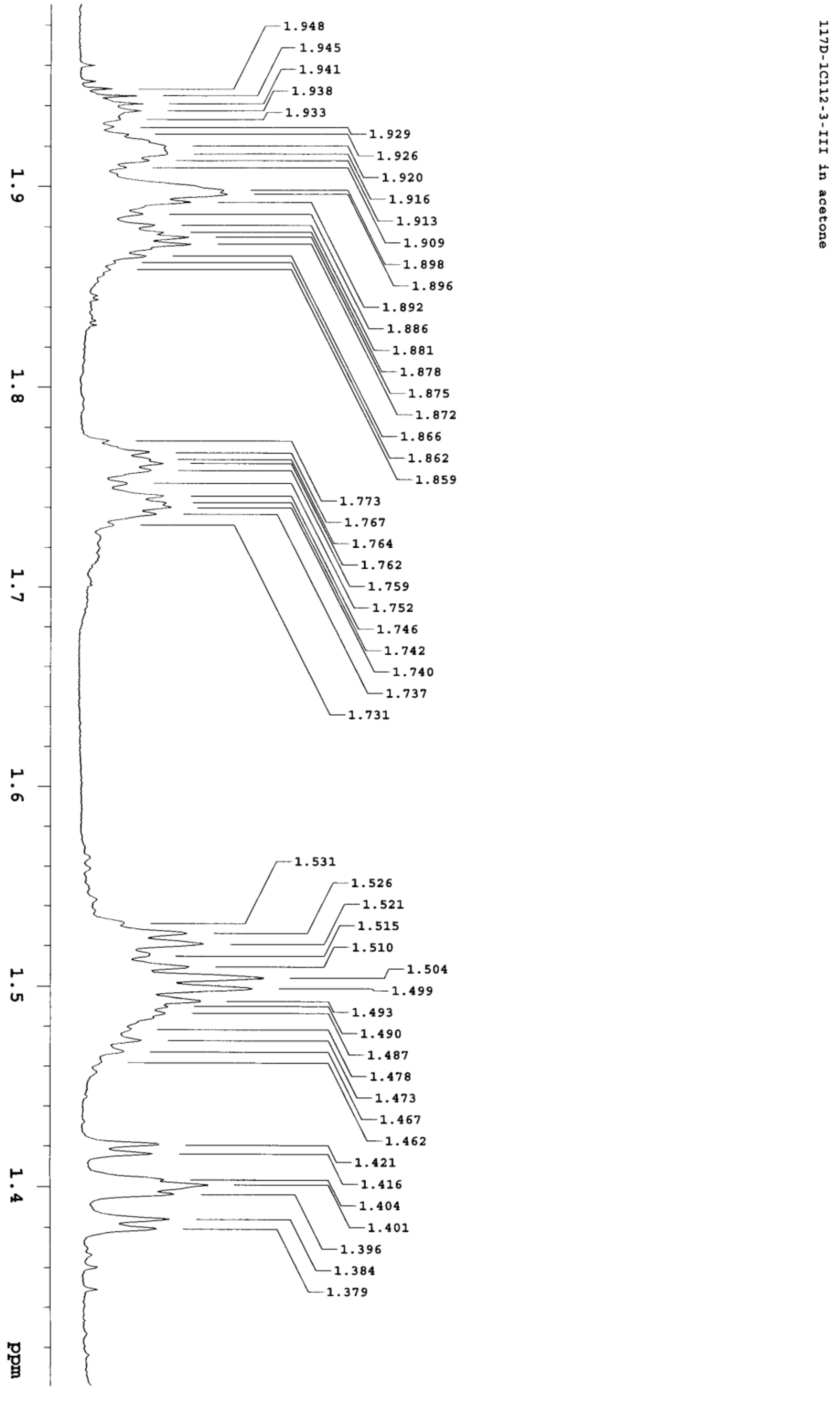
117D-1C112-3-III in acetone
1.9
1.8
1.7
1.6
1.5
1.4
ppm
1.948
1.945
1.941
1.938
1.933
1.929
1.926
1.920
1.916
1.913
1.909
1.898
1.896
1.892
1.886
1.881
1.878
1.875
1.872
1.866
1.862
1.859
1.773
1.767
1.764
1.762
1.759
1.752
1.746
1.742
1.740
1.737
1.731
1.531
1.526
1.521
1.515
1.510
1.504
1.499
1.493
1.490
1.487
1.478
1.473
1.467
1.462
1.421
1.416
1.404
1.401
1.396
1.384
1.379

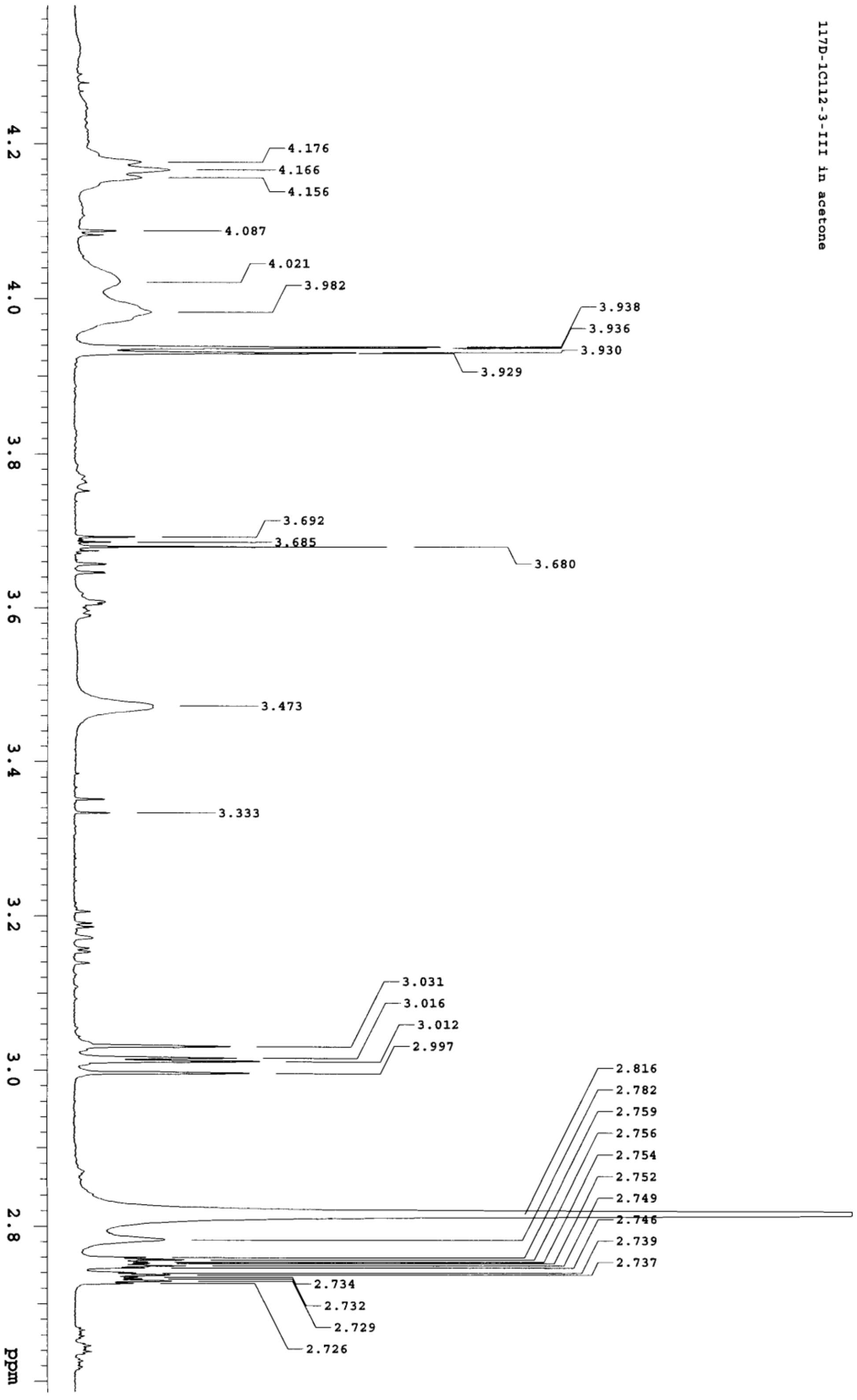
117D-1C112-3-III in acetone
4.176
4.166
4.156
4.087
4.021
3.982
3.938
3.936
3.930
3.929
3.692
3.685
3.680
3.473
3.333
3.031
3.016
3.012
2.997
2.816
2.782
2.759
2.756
2.754
2.752
2.749
2.746
2.739
2.737
2.734
2.732
2.729
2.726
4.2
4.0
3.8
3.6
3.4
3.2
3.0
2.8
ppm

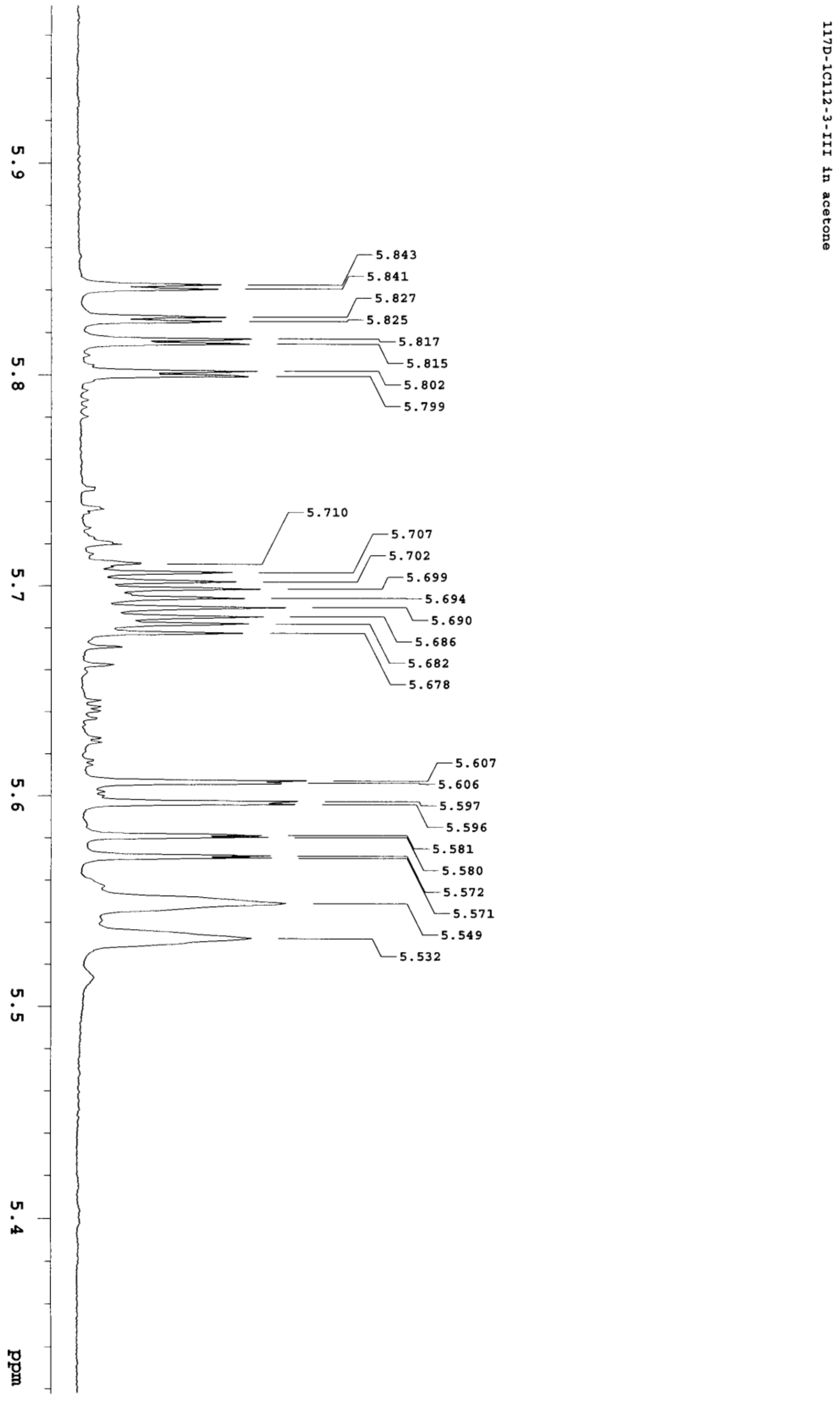
117D-1C112-3-III in acetone
5.843
5.841
5.827
5.825
5.817
5.815
5.802
5.799
5.710
5.707
5.702
5.699
5.694
5.690
5.686
5.682
5.678
5.607
5.606
5.597
5.596
5.581
5.580
5.572
5.571
5.549
5.532
5.9
5.8
5.7
5.6
5.5
5.4
ppm

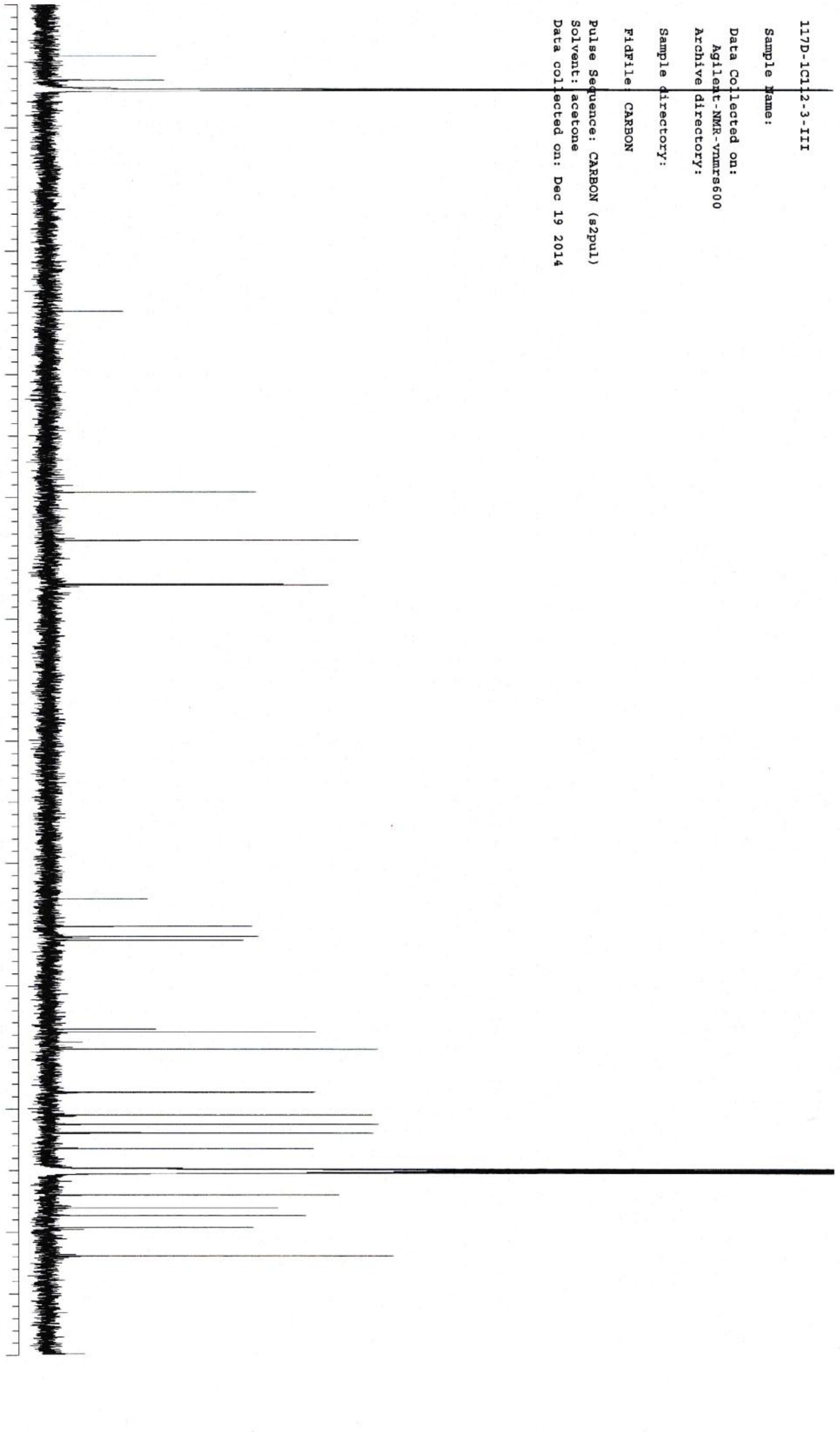
117D-1C112-3-III
Sample Name:
Data Collected on:
Agilent-NMR-vnmrs600
Archive directory:
Sample directory:
FidFile: CARBON
Pulse Sequence: CARBON (s2pul)
Solvent: acetone
Data collected on: Dec 19 2014
200
180
160
140
120
100
80
60
40
20
ppm

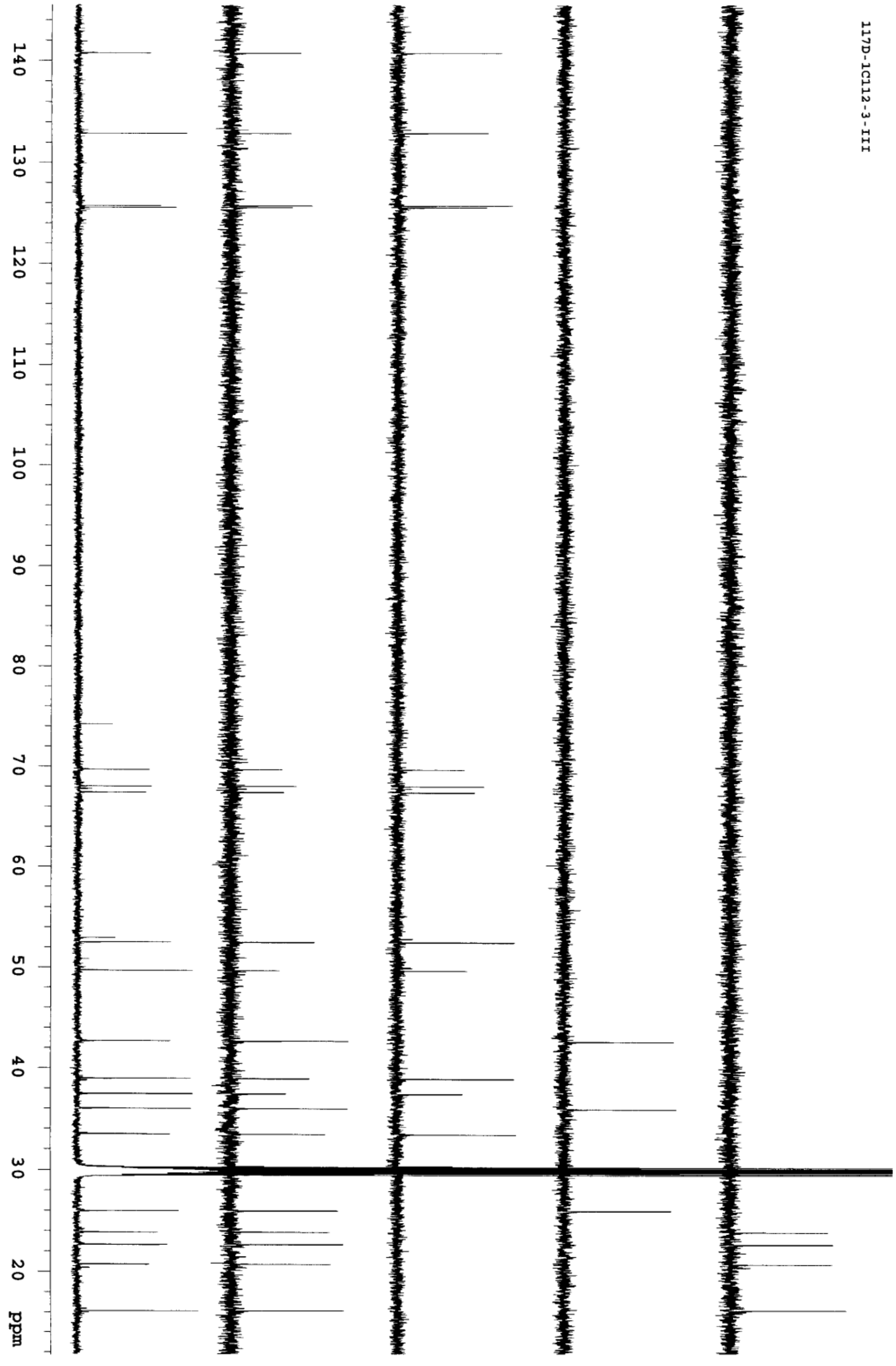
117D-1C112-3-III
140
130
120
110
100
90
80
70
60
50
40
30
20
ppm

Figure S8 ^{1}H-^{1}H COSY of 2

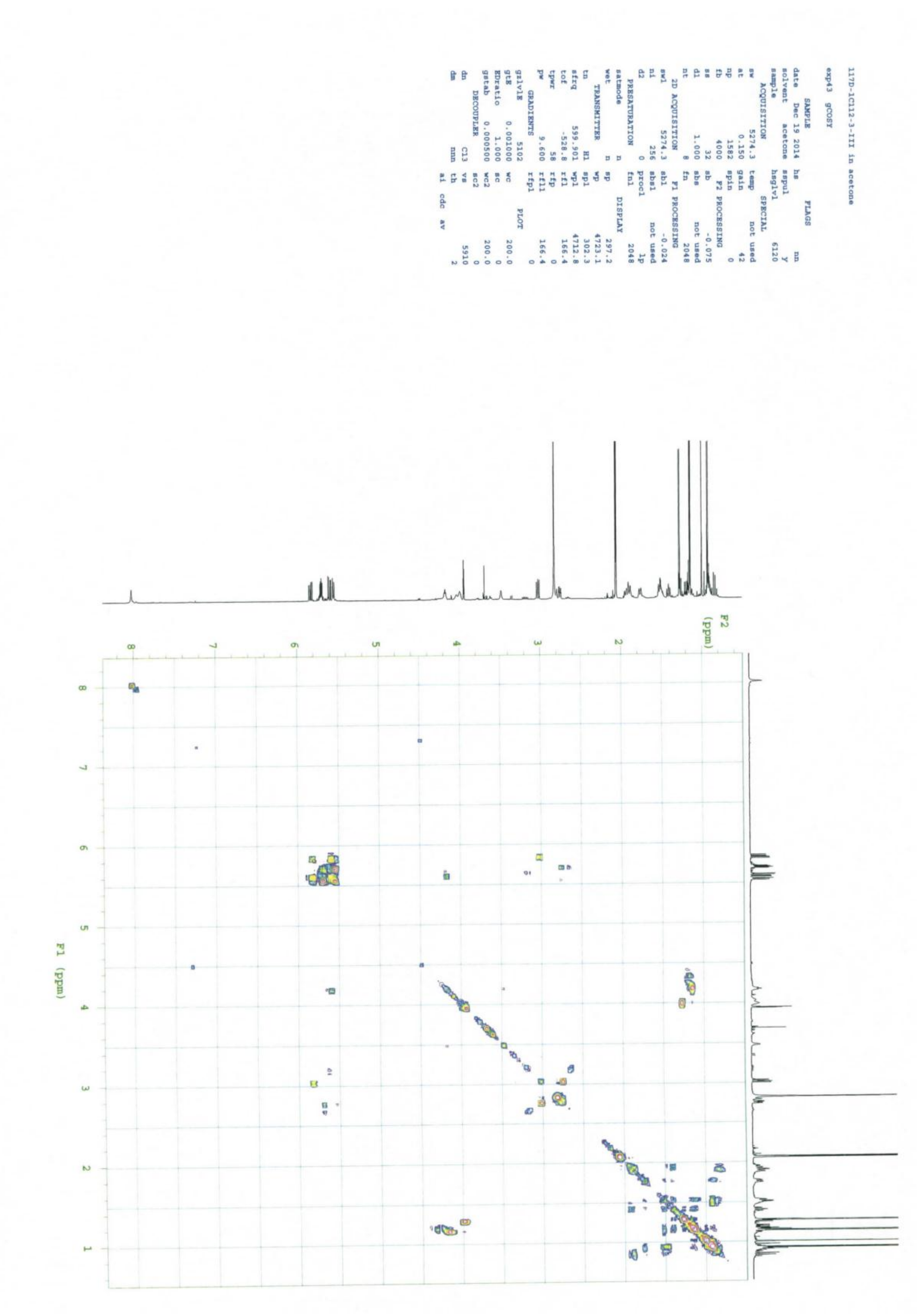

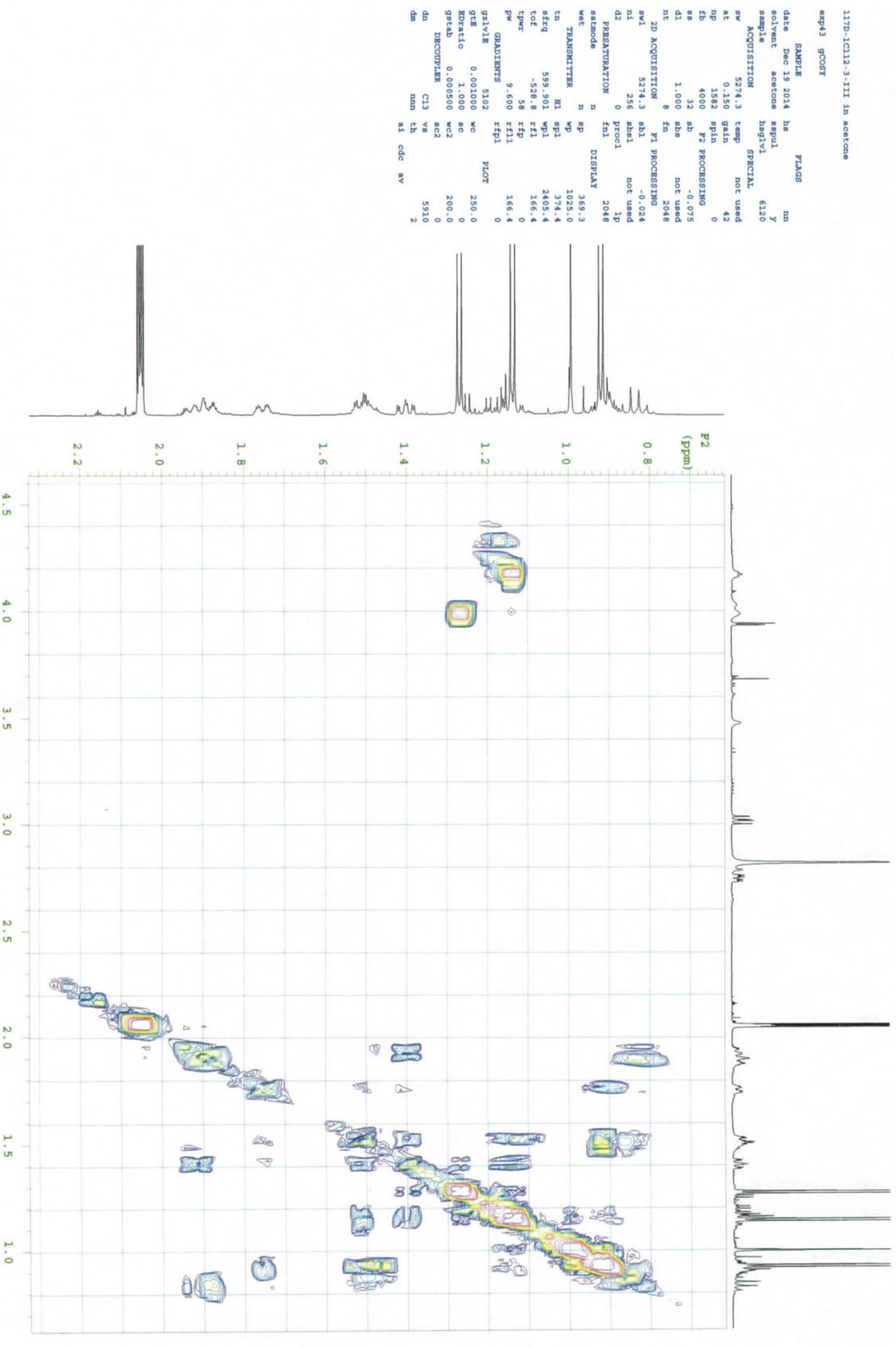
117D-1C112-3-III in acetone
exp43 gCOSY
F2 (ppm)
F1 (ppm)

Figure S9　NOESY of 2

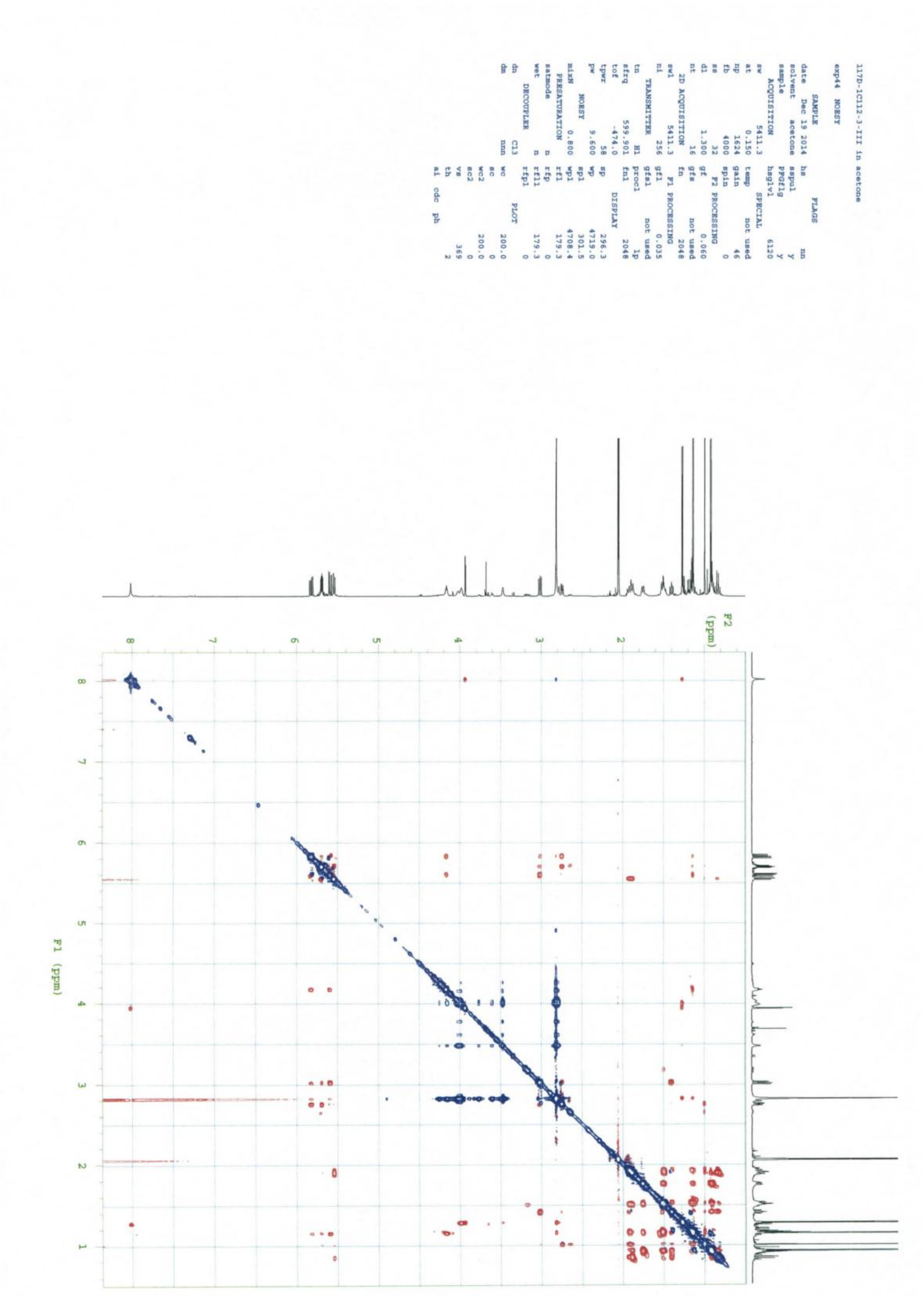

117D-1C112-3-III in acetone

exp44 NOESY

SAMPLE		FLAGS	
date	Dec 19 2014	hs	nn
solvent	acetone	sspul	y
sample		PFGflg	y
ACQUISITION		hsglvl	6120
sw	5411.3	SPECIAL	
at	0.150	temp	not used
np	1624	gain	46
fb	4000	spin	0
ss	32	F2 PROCESSING	
d1	1.300	gf	0.060
nt	16	gfs	not used
2D ACQUISITION		fn	2048
sw1	5411.3	F1 PROCESSING	
ni	256	gf1	0.035
TRANSMITTER		gfs1	not used
tn	H1	proc1	lp
sfrq	599.901	fn1	2048
tof	-474.0	DISPLAY	
tpwr	58	sp	407.2
pw	9.600	wp	914.2
NOESY		sp1	412.5
mixN	0.800	wp1	914.2
PRESATURATION		rfl	179.3
satmode	n	rfp	0
wet	n	rfl1	179.3
DECOUPLER		rfp1	0
dn	C13	PLOT	
dm	nnn	wc	200.0
		sc	0
		wc2	200.0
		sc2	0
		vs	369
		th	2
		ai cdc ph	

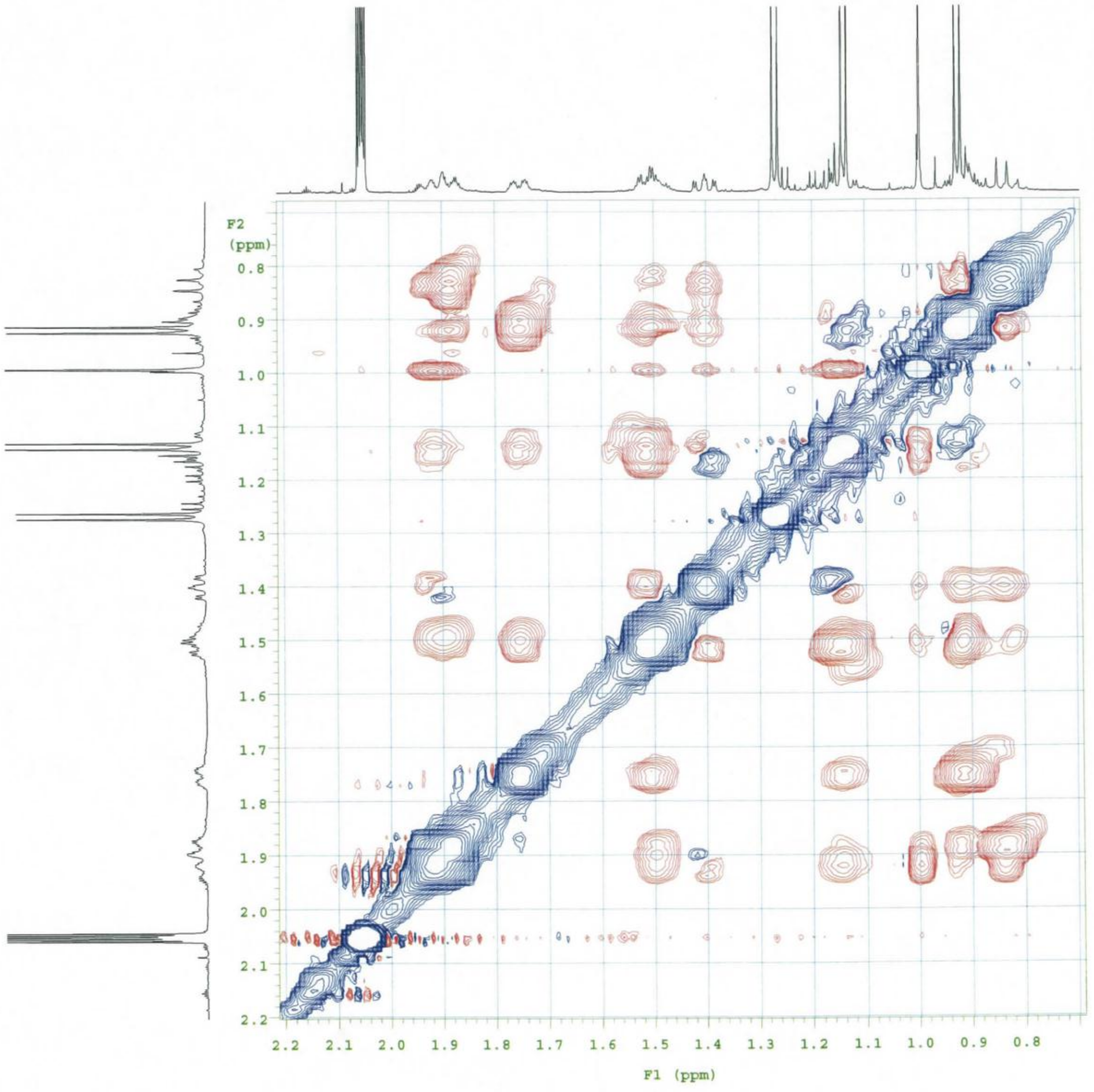

Figure S10　HMQC of 2

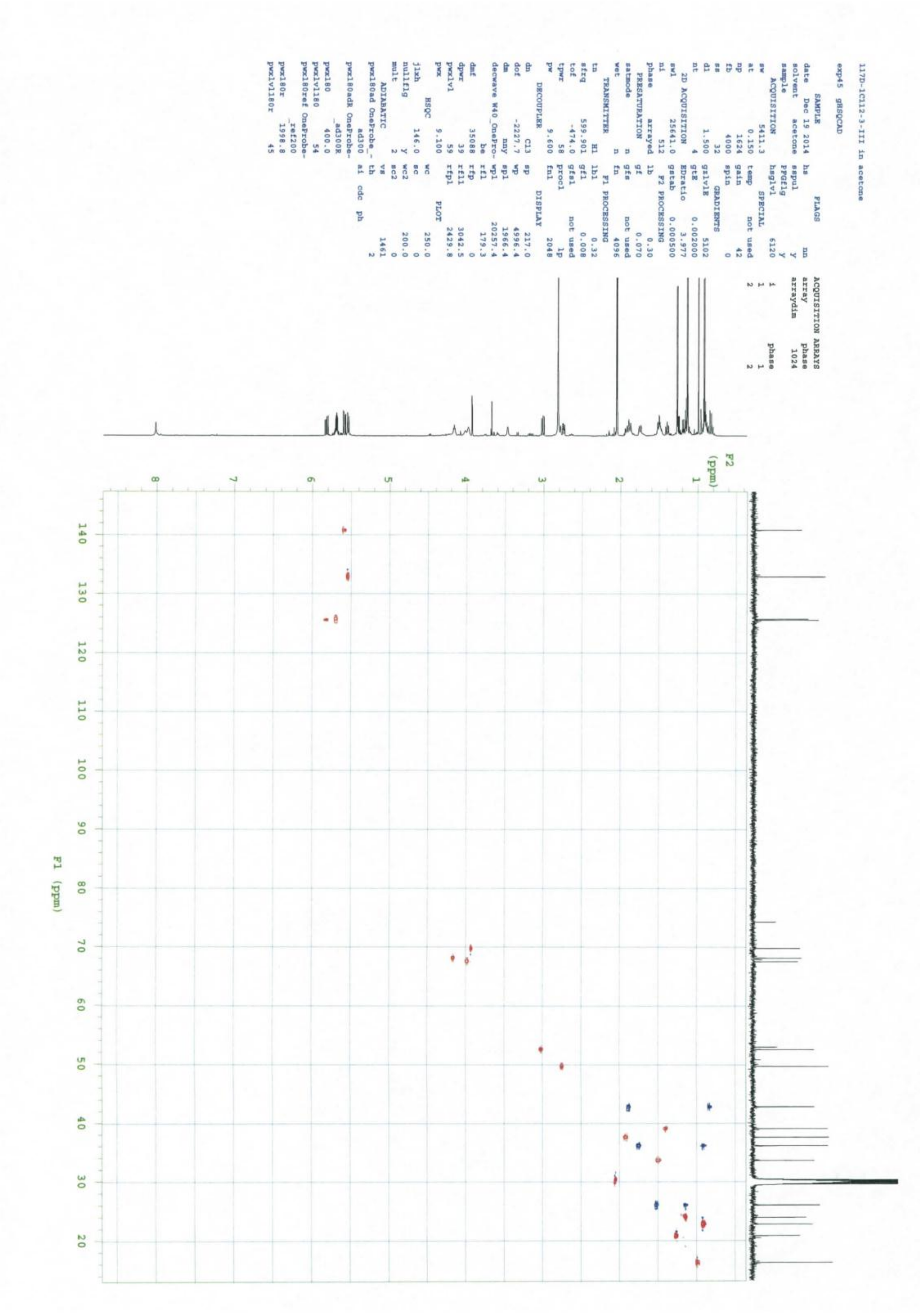

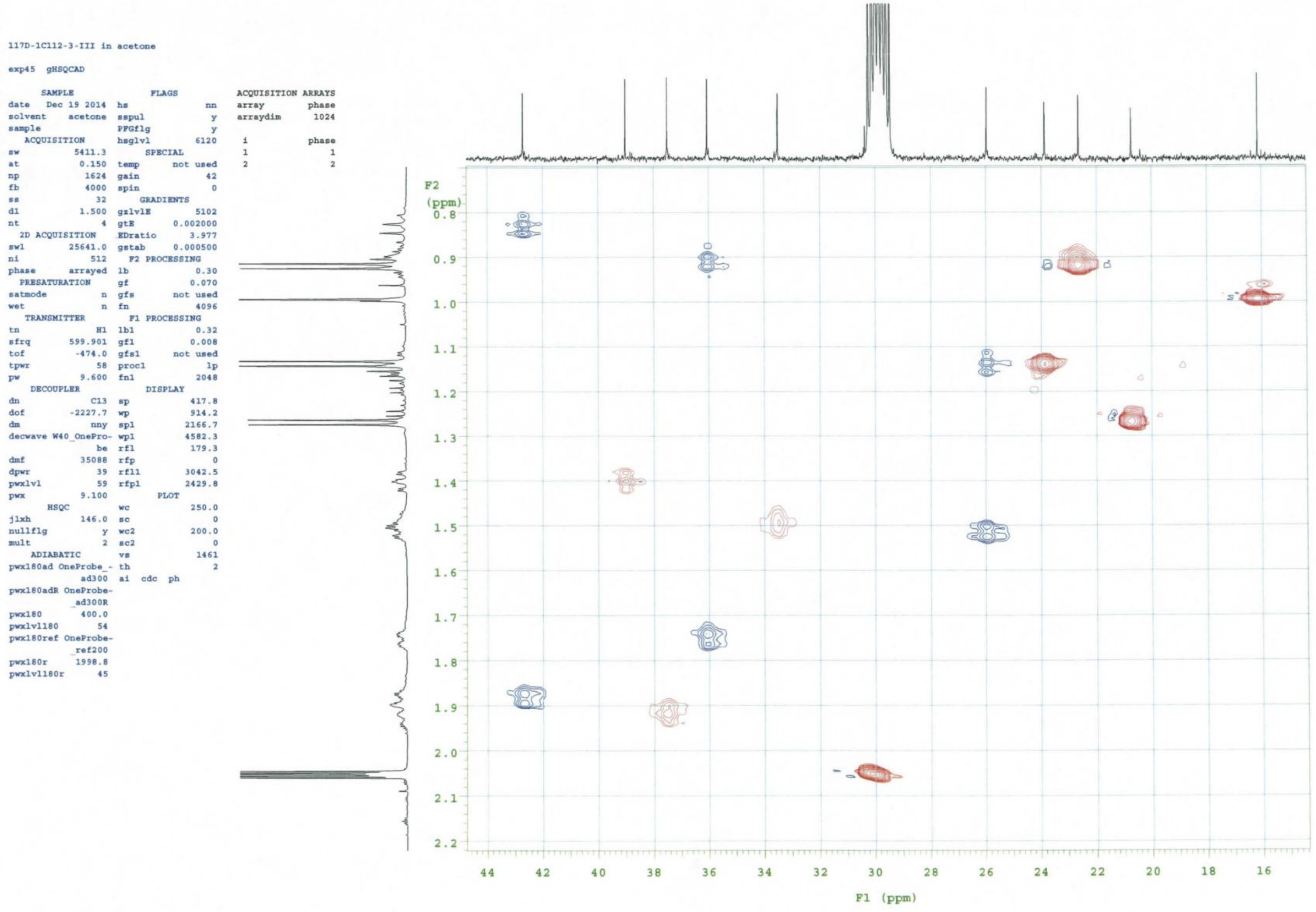
117D-1C112-3-III in acetone
exp45 gHSQCAD
SAMPLE
date Dec 19 2014
solvent acetone
sample
ACQUISITION
sw 5411.3
at 0.150
np 1624
fb 4000
ss 32
d1 1.500
nt 4
2D ACQUISITION
sw1 25641.0
ni 512
phase arrayed
PRESATURATION
satmode n
wet n
TRANSMITTER
tn H1
sfrq 599.901
tof -474.0
tpwr 58
pw 9.600
DECOUPLER
dn C13
dof -2227.7
dm nny
decwave W40_OnePro-be
dmf 35088
dpwr 39
pwxlvl 59
pwx 9.100
HSQC
j1xh 146.0
nullflg y
mult 2
ADIABATIC
pwx180ad OneProbe_-ad300
pwx180adR OneProbe-_ad300R
pwx180 400.0
pwxlvl180 54
pwx180ref OneProbe-_ref200
pwx180r 1998.8
pwxlvl180r 45
FLAGS
hs nn
sspul y
PFGflg y
hsglvl 6120
SPECIAL
temp not used
gain 42
spin 0
GRADIENTS
gzlvlE 5102
gtE 0.002000
EDratio 3.977
gstab 0.000500
F2 PROCESSING
lb 0.30
gf 0.070
gfs not used
fn 4096
F1 PROCESSING
lb1 0.32
gf1 0.008
gfs1 not used
proc1 lp
fn1 2048
DISPLAY
sp 417.8
wp 914.2
sp1 2166.7
wp1 4582.3
rfl 179.3
rfp 0
rfl1 3042.5
rfp1 2429.8
PLOT
wc 250.0
sc 0
wc2 200.0
sc2 0
vs 1461
th 2
ai cdc ph
ACQUISITION ARRAYS
array phase
arraydim 1024
i phase
1 1
2 2
F2 (ppm)
0.8
0.9
1.0
1.1
1.2
1.3
1.4
1.5
1.6
1.7
1.8
1.9
2.0
2.1
2.2
44 42 40 38 36 34 32 30 28 26 24 22 20 18 16
F1 (ppm)

Figure S11 HMBC of 2

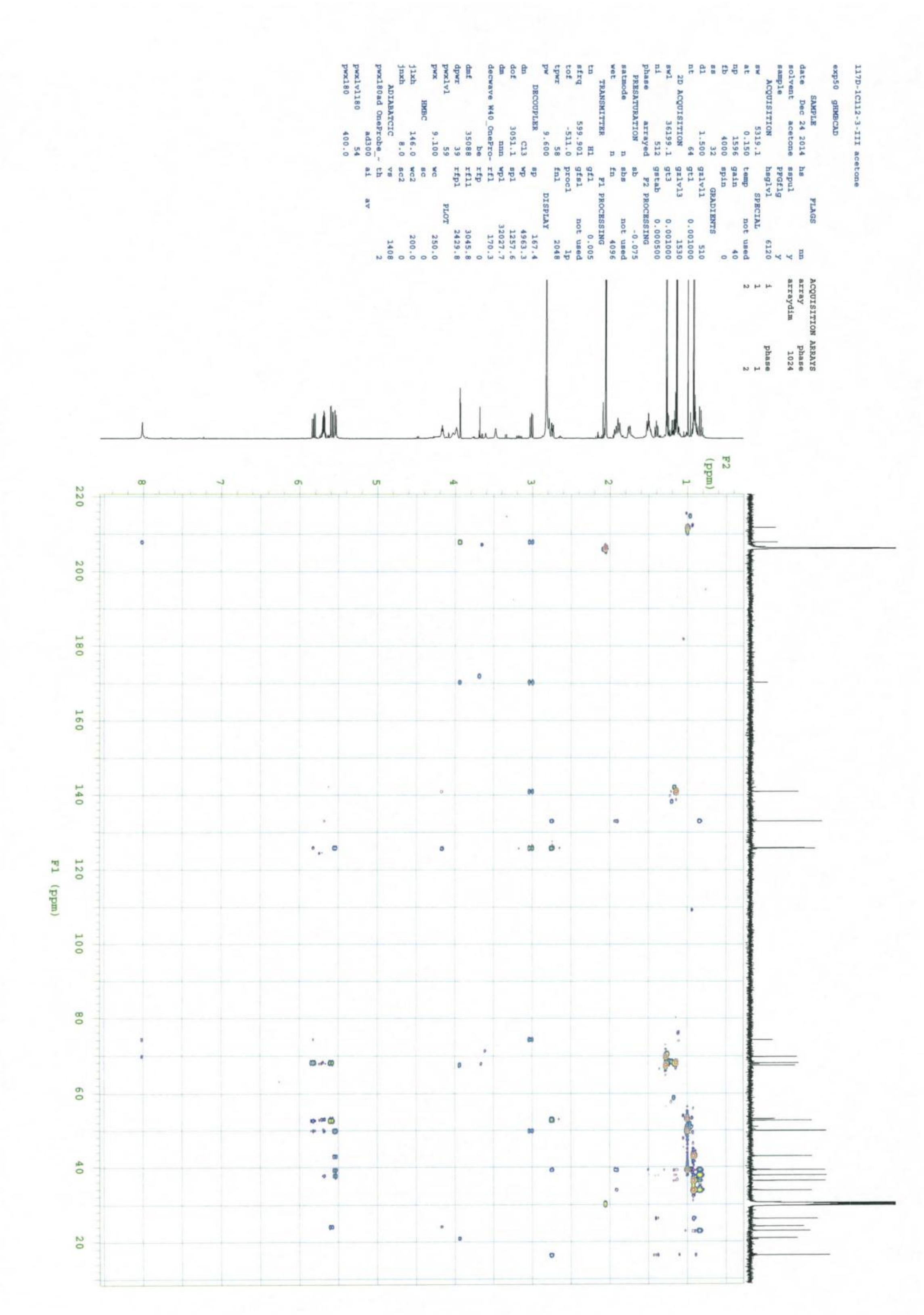

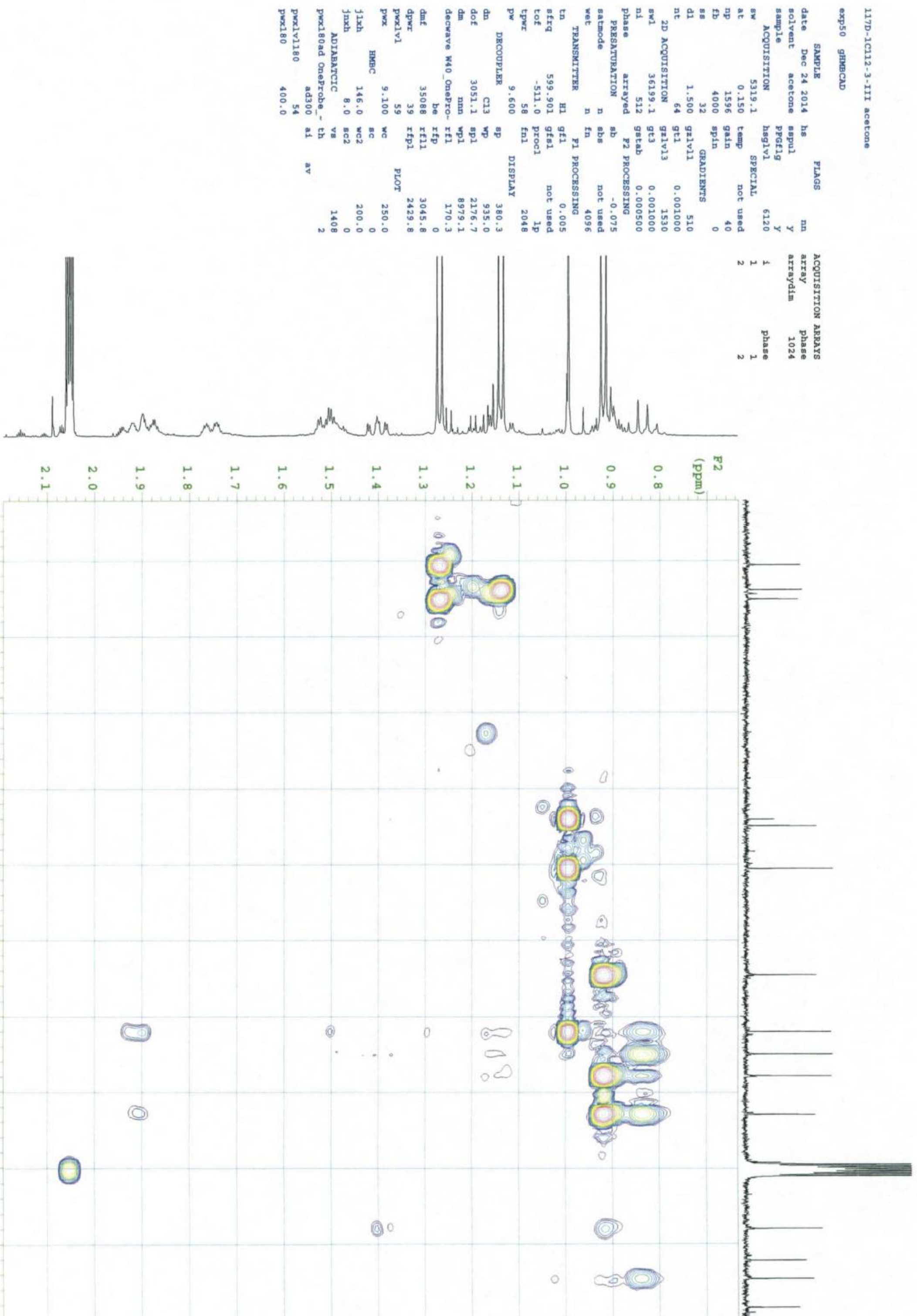
117D-1C112-3-III acetone
exp50 gHMBCAD
SAMPLE
date Dec 24 2014
solvent acetone
sample
ACQUISITION
sw 5319.1
at 0.150
np 1596
fb 4000
ss 32
d1 1.500
nt 64
2D ACQUISITION
sw1 36199.1
ni 512
phase arrayed
PRESATURATION
satmode n
wet n
TRANSMITTER
tn H1
sfrq 599.901
tof -511.0
tpwr 58
pw 9.600
DECOUPLER
dn C13
dof 3051.1
dm nnn
decwave W40_OnePro-
be
dmf 35088
dpwr 39
pwxlvl 59
pwx 9.100
HMBC
j1xh 146.0
jnxh 8.0
ADIABATCIC
pwx180ad OneProbe_-
ad300
pwxlvl180 54
pwx180 400.0
FLAGS
hs nn
sspul y
PFGflg y
hsglvl 6120
SPECIAL
temp not used
gain 40
spin 0
GRADIENTS
gzlvl1 510
gt1 0.001000
gzlvl3 1530
gt3 0.001000
gstab 0.000500
F2 PROCESSING
sb -0.075
sbs not used
fn 4096
F1 PROCESSING
gf1 0.005
gfs1 not used
proc1 lp
fn1 2048
DISPLAY
sp 380.3
wp 935.0
sp1 2176.7
wp1 8979.1
rfl 170.3
rfp 0
rfl1 3045.8
rfp1 2429.8
PLOT
wc 250.0
sc 0
wc2 200.0
sc2 0
vs 1408
th 2
ai av
ACQUISITION ARRAYS
array phase
arraydim 1024
i phase
1 1
2 2
F2 (ppm)
F1 (ppm)

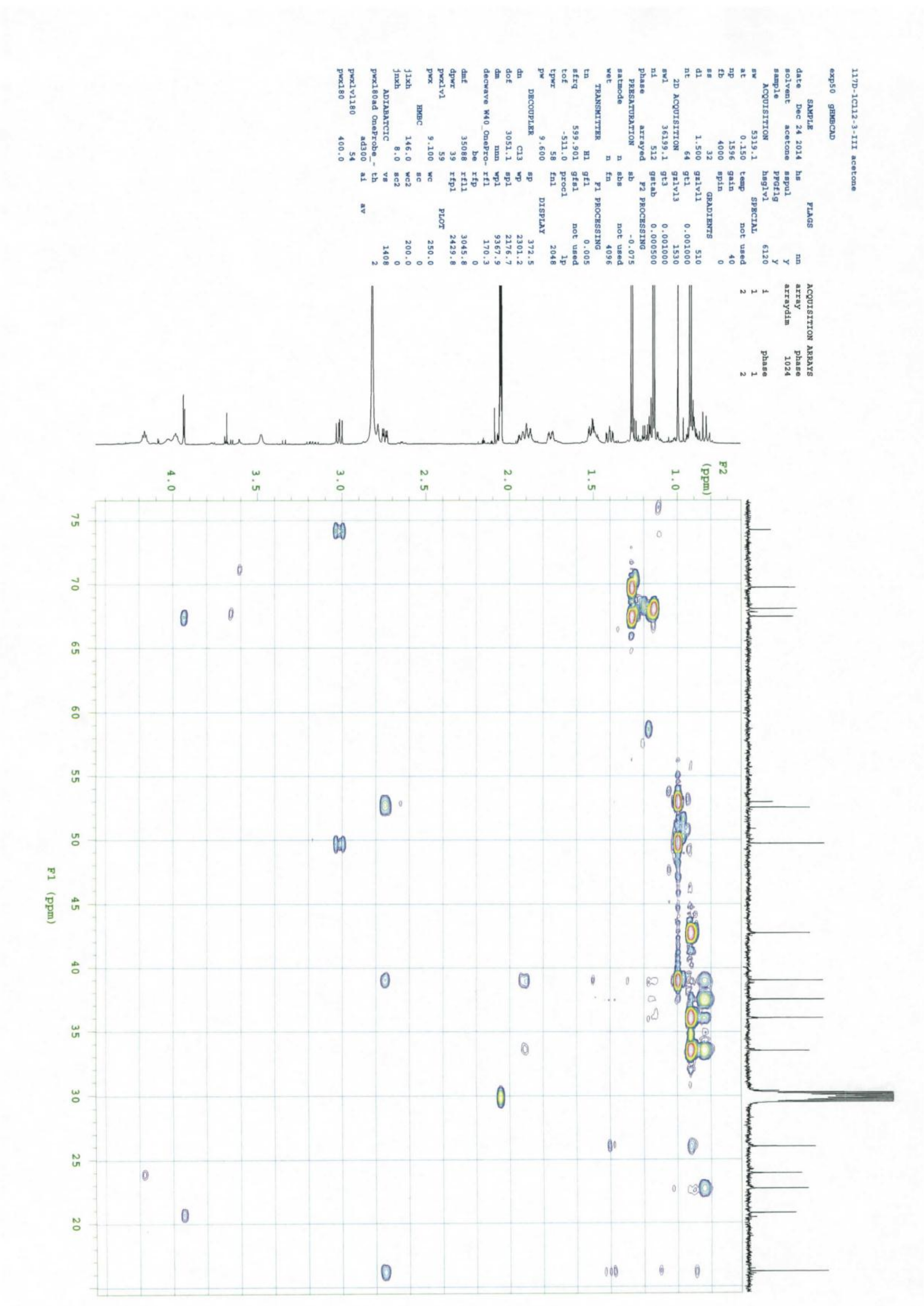
117D-1C112-3-III acetone
exp50 gHMBCAD
F2 (ppm)
F1 (ppm)

Figure S12 FABMS of 2

[Elemental Composition] 2

Page: 1

Data : 1503094

Date : 12-Mar-2015 15:41

Sample: 117D-1C112-3-III

Note : Matrix; G

Inlet : Direct

Ion Mode : FAB+

RT : 6.33 min

Scan#: (23,28)

Elements : C 28/20, H 38/30, N 3/0, O 7/0, Na 1/0

Mass Tolerance : 20ppm, 1mmu if m/z > 50

Unsaturation (U.S.) : -1.0 - 50.0

Observed m/z	Int%	Err[ppm / mmu]	U.S.	Composition
416.2430	80.6	-1.6 / -0.7	8.5	C 24 H 34 N O 5
		-2.2 / -0.9	10.0	C 25 H 33 N 2 O 2 Na
438.2263	59.4	+1.5 / +0.7	8.5	C 24 H 33 N O 5 Na

[Mass Spectrum]
Data : 1503079 Date : 11-Mar-2015 11:36
Sample: 117D-1C112-3-III
Note : Matrix; G
Inlet : Direct Ion Mode : FAB+
Spectrum Type : Normal Ion [MF-Linear]
RT : 1.11 min Scan# : (5,15)-k((30,40))[k=1.0]
BP : m/z 398.0000 Int. : 141.78
Output m/z range : 20.0000 to 550.0000 Cut Level : 0.00 %

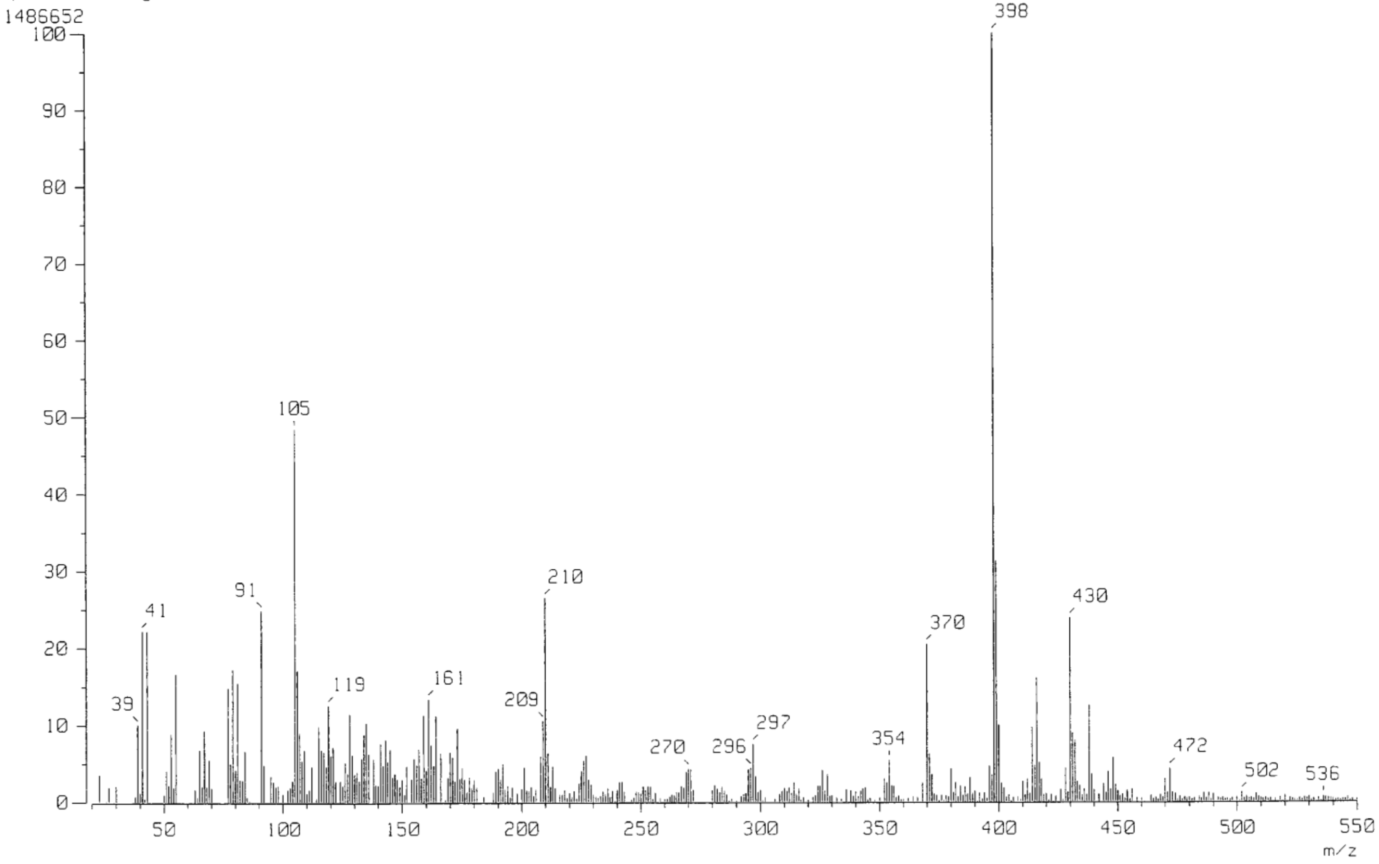

Supplement: Supplementary file 1 [file marinedrugs-17-00218-s001.zip › marinedrugs-480817 Suppl/S.M-R1/SM-vol2.pdf]
