# Supplementary material for: Altercrasins A–E, Decalin Derivatives, from a Sea-Urchin-Derived Alternaria sp.: Isolation and Structural Analysis Including Stereochemistry
Source: Mar Drugs. 2019 Apr 11;17(4):218. doi: 10.3390/md17040218 (PMC6521173; doi:10.3390/md17040218)
Supplement: Supplementary file 1 [file marinedrugs-17-00218-s001.zip › marinedrugs-480817 Suppl/S.M-R1/SM-vol3.pdf]

Figure S13 **^{1}H and ^{13}C NMR spectra in acetone-d_6 of 3**

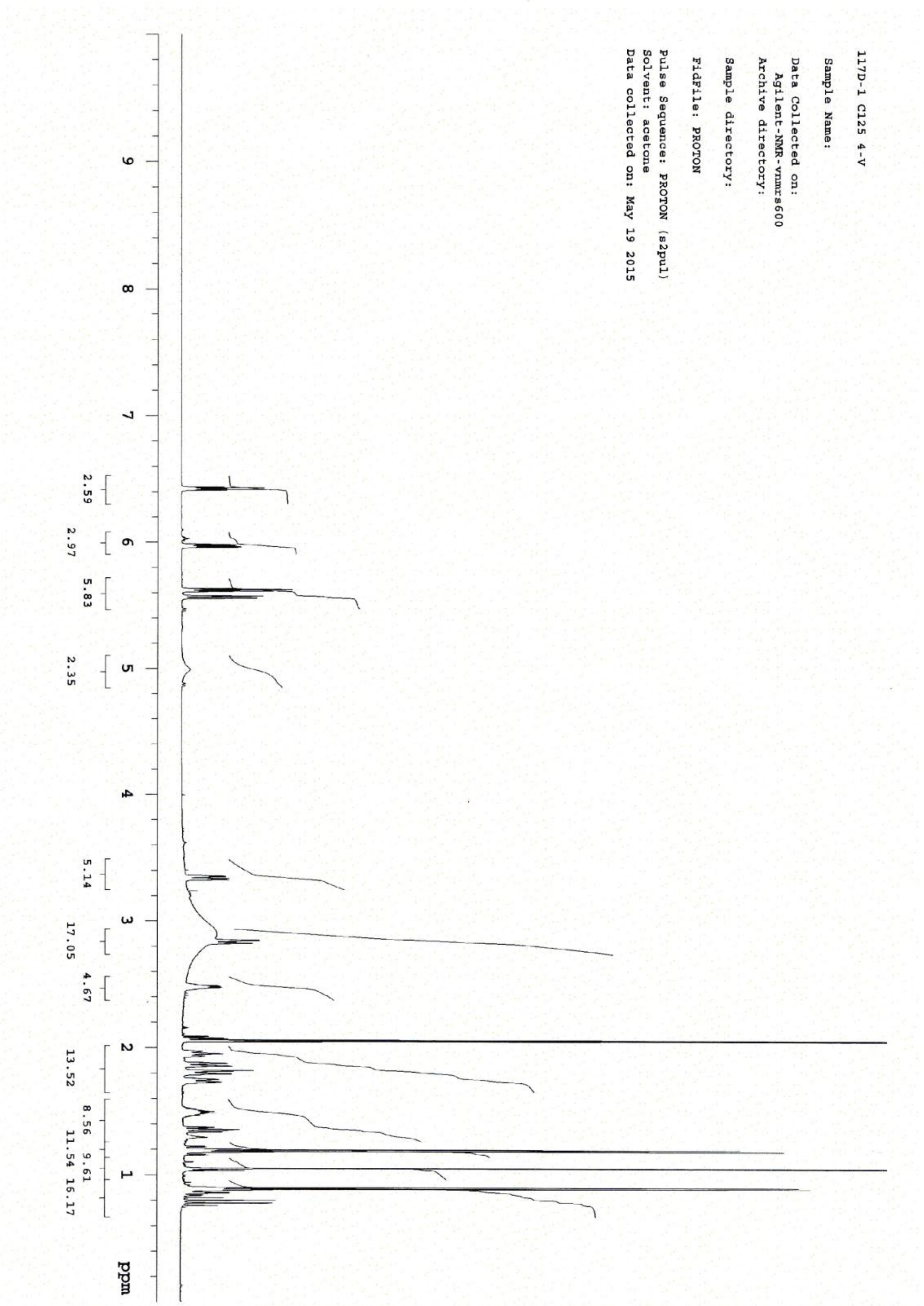

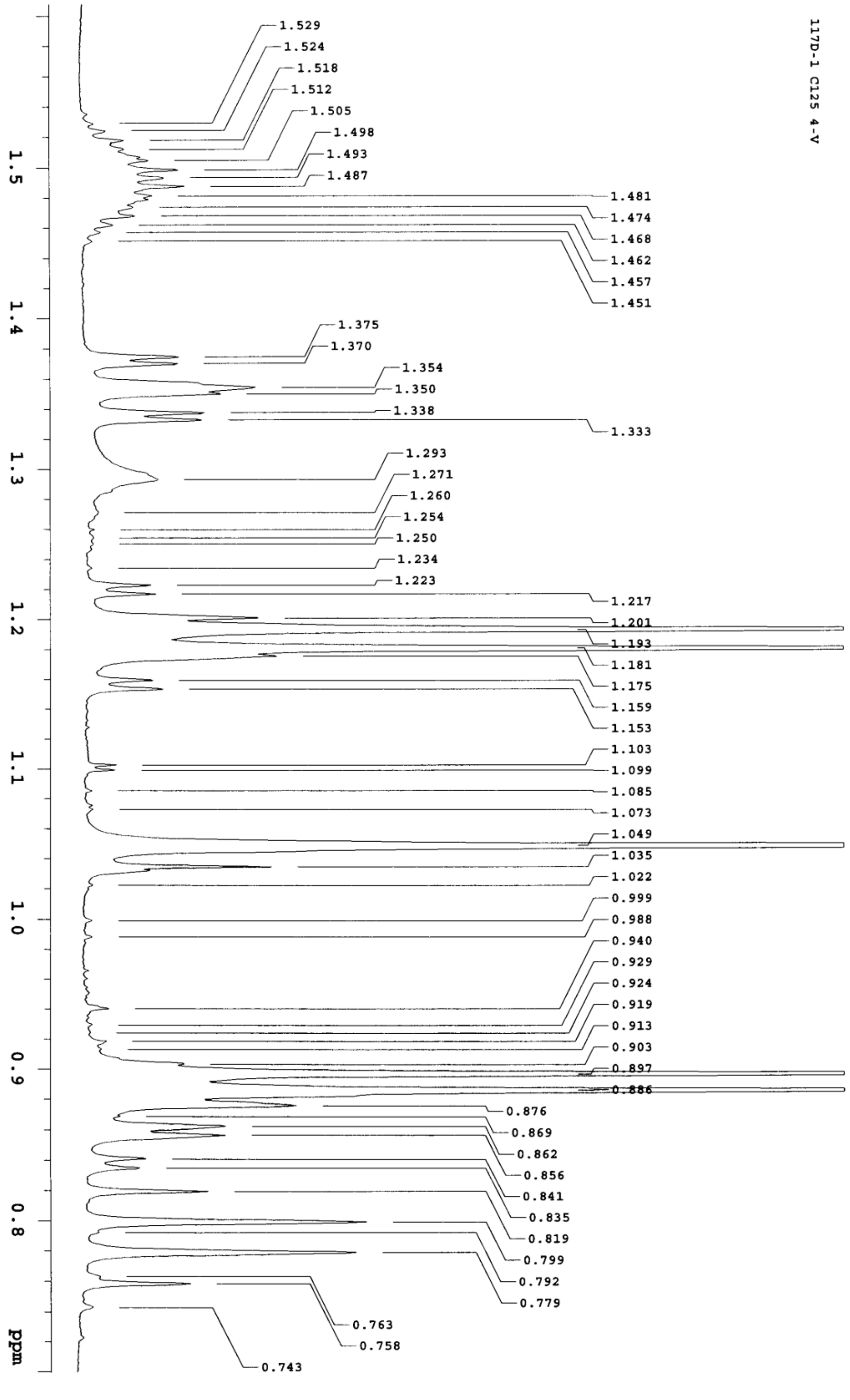
117D-1 C125 4-V
1.529
1.524
1.518
1.512
1.505
1.498
1.493
1.487
1.481
1.474
1.468
1.462
1.457
1.451
1.375
1.370
1.354
1.350
1.338
1.333
1.293
1.271
1.260
1.254
1.250
1.234
1.223
1.217
1.201
1.193
1.181
1.175
1.159
1.153
1.103
1.099
1.085
1.073
1.049
1.035
1.022
0.999
0.988
0.940
0.929
0.924
0.919
0.913
0.903
0.897
0.886
0.876
0.869
0.862
0.856
0.841
0.835
0.819
0.799
0.792
0.779
0.763
0.758
0.743
1.5
1.4
1.3
1.2
1.1
1.0
0.9
0.8
ppm

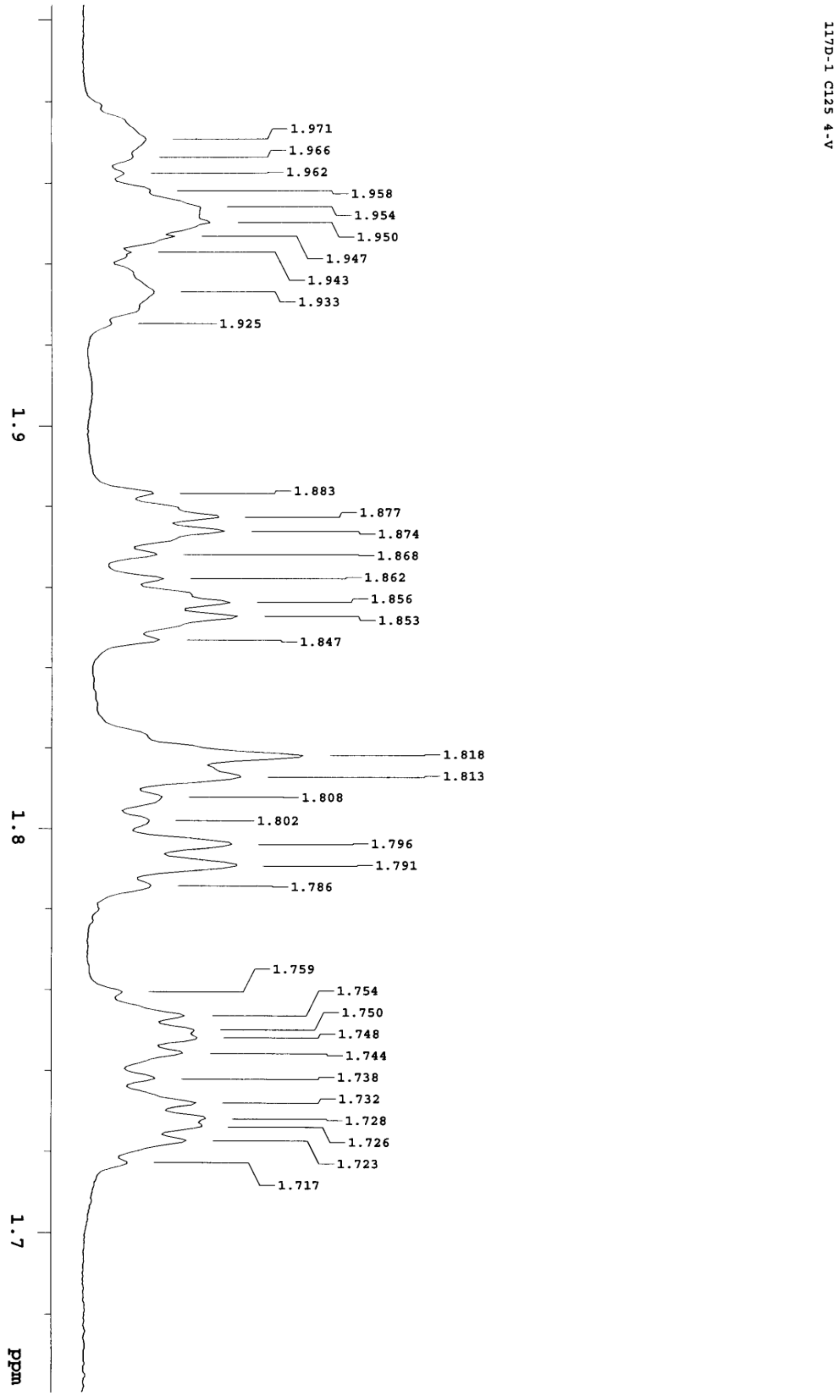
117D-1 C125 4-V
1.971
1.966
1.962
1.958
1.954
1.950
1.947
1.943
1.933
1.925
1.883
1.877
1.874
1.868
1.862
1.856
1.853
1.847
1.818
1.813
1.808
1.802
1.796
1.791
1.786
1.759
1.754
1.750
1.748
1.744
1.738
1.732
1.728
1.726
1.723
1.717
1.9
1.8
1.7
ppm

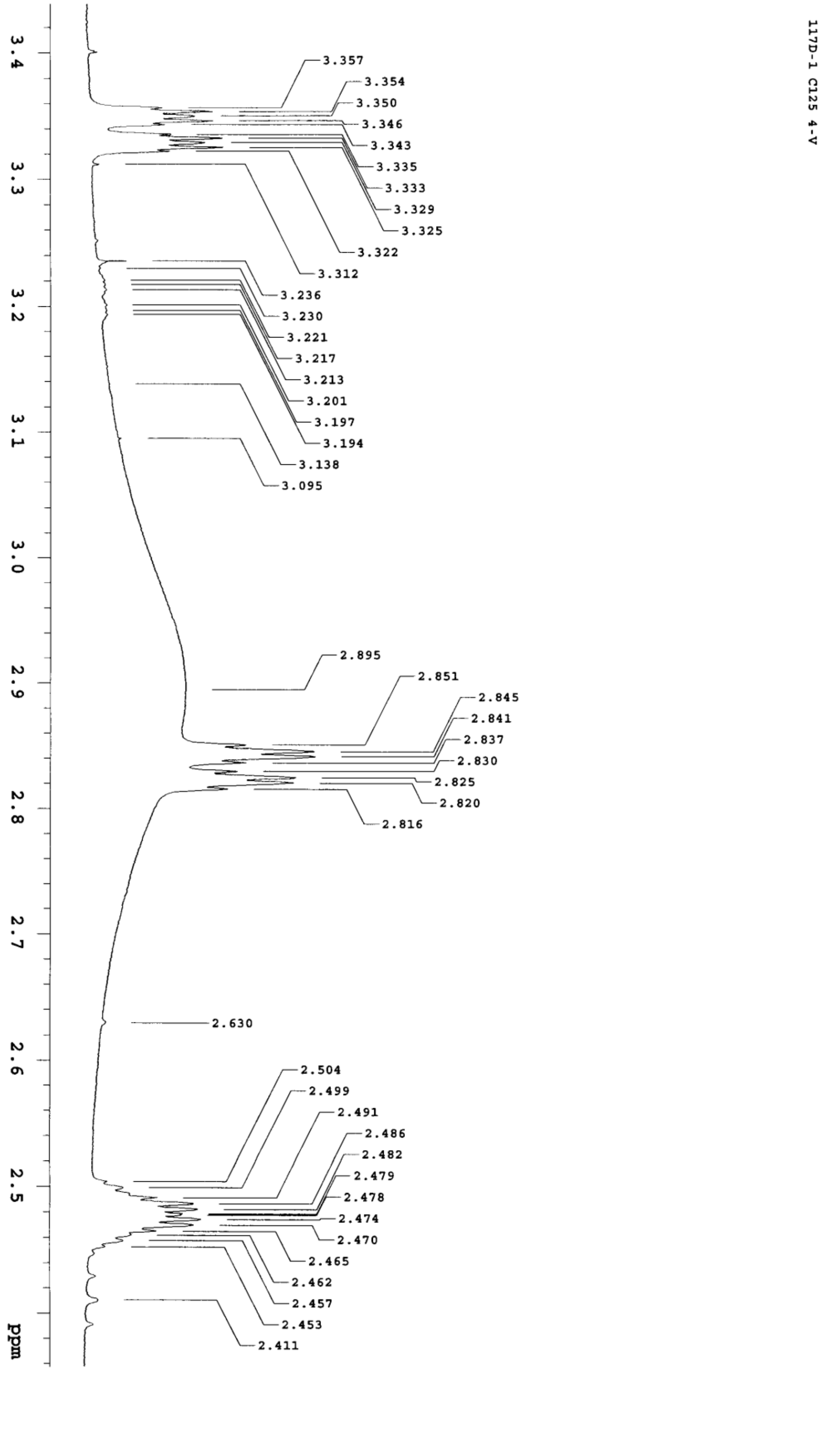
117D-1 C125 4-V
3.357
3.354
3.350
3.346
3.343
3.335
3.333
3.329
3.325
3.322
3.312
3.236
3.230
3.221
3.217
3.213
3.201
3.197
3.194
3.138
3.095
2.895
2.851
2.845
2.841
2.837
2.830
2.825
2.820
2.816
2.630
2.504
2.499
2.491
2.486
2.482
2.479
2.478
2.474
2.470
2.465
2.462
2.457
2.453
2.411
3.4
3.3
3.2
3.1
3.0
2.9
2.8
2.7
2.6
2.5
ppm

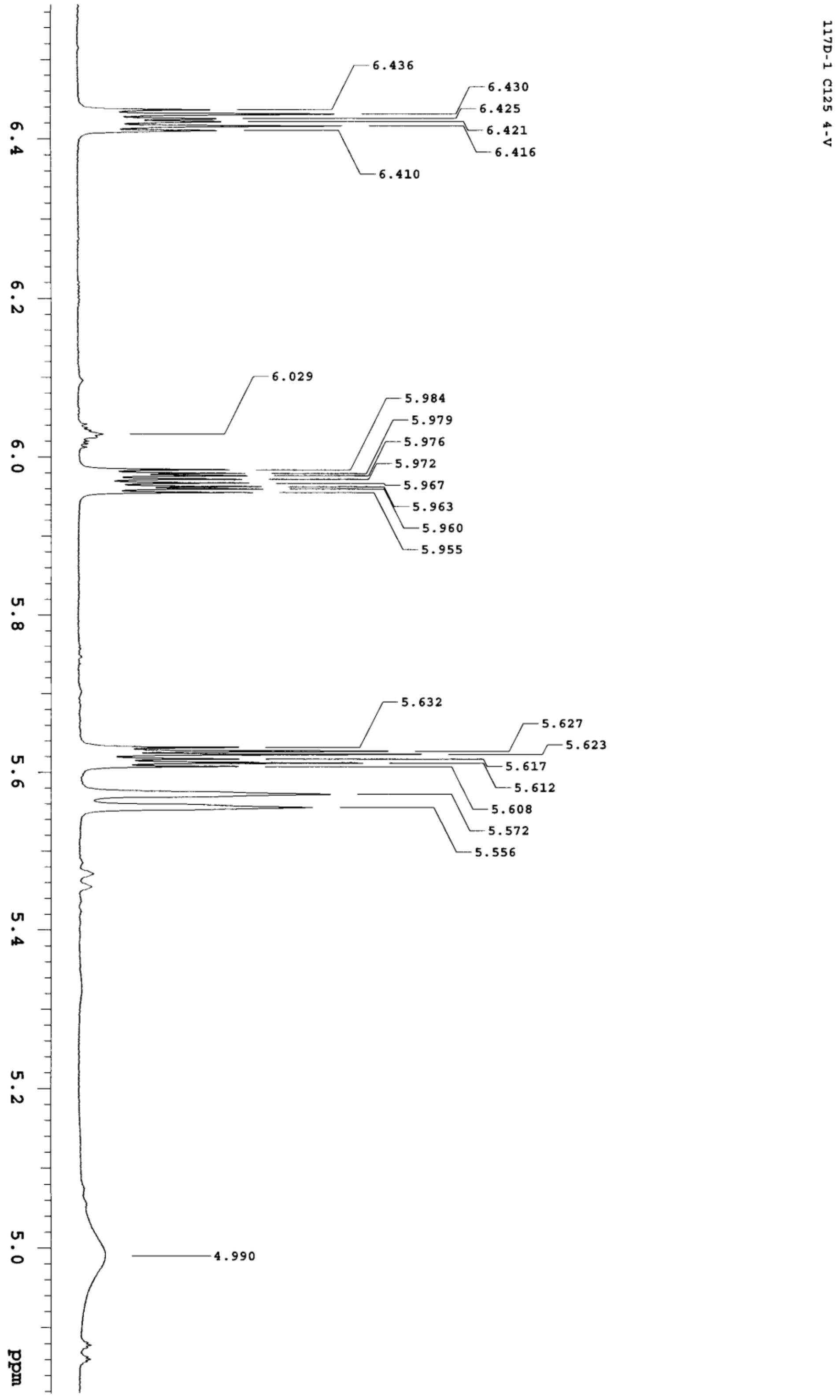
117D-1 C125 4-V
6.436
6.430
6.425
6.421
6.416
6.410
6.029
5.984
5.979
5.976
5.972
5.967
5.963
5.960
5.955
5.632
5.627
5.623
5.617
5.612
5.608
5.572
5.556
4.990
6.4
6.2
6.0
5.8
5.6
5.4
5.2
5.0
ppm

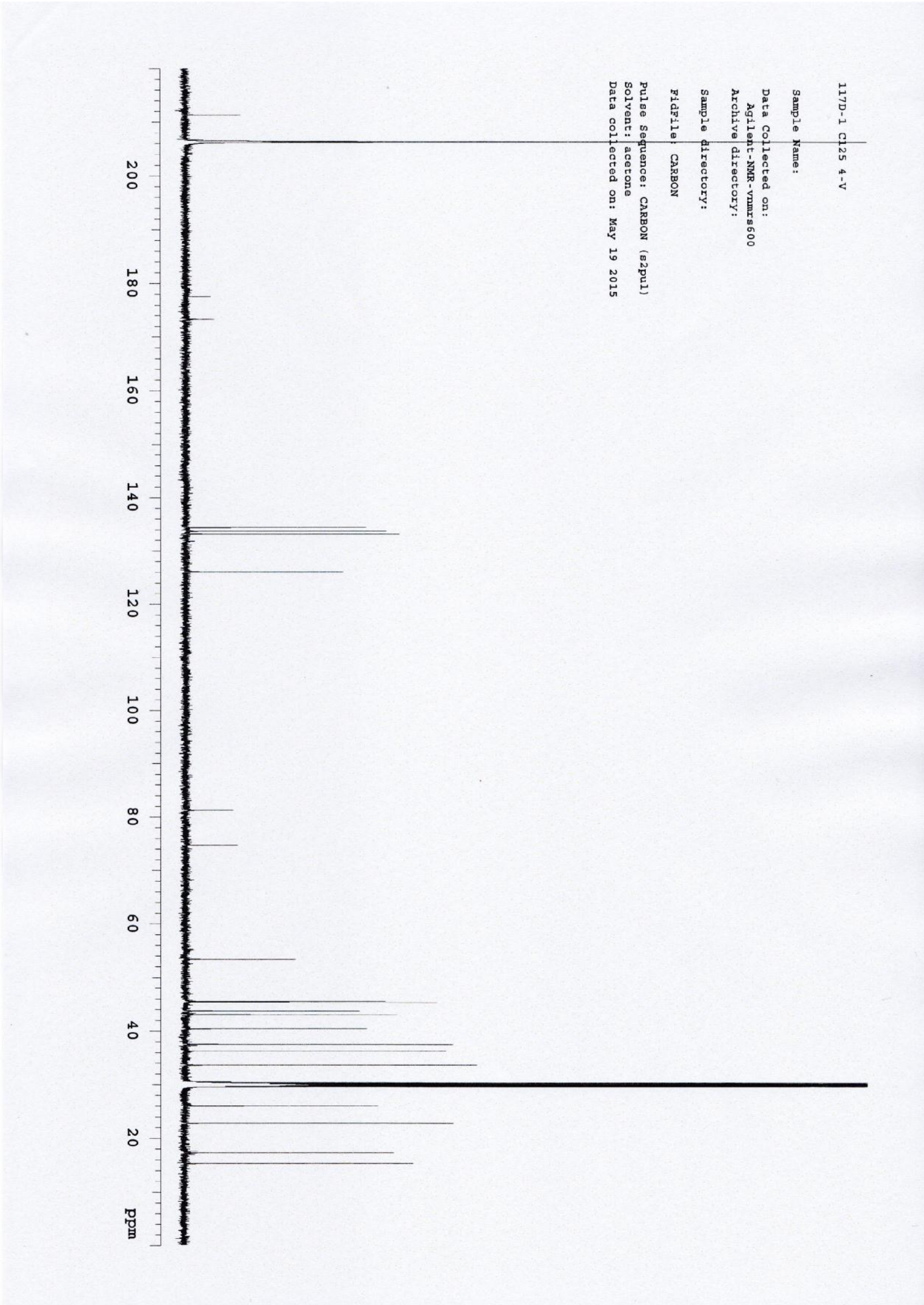
117D-1 C125 4-V
Sample Name:
Data Collected on:
Agilent-NMR-vnmrs600
Archive directory:
Sample directory:
FidFile: CARBON
Pulse Sequence: CARBON (s2pul)
Solvent: acetone
Data collected on: May 19 2015
200
180
160
140
120
100
80
60
40
20
ppm

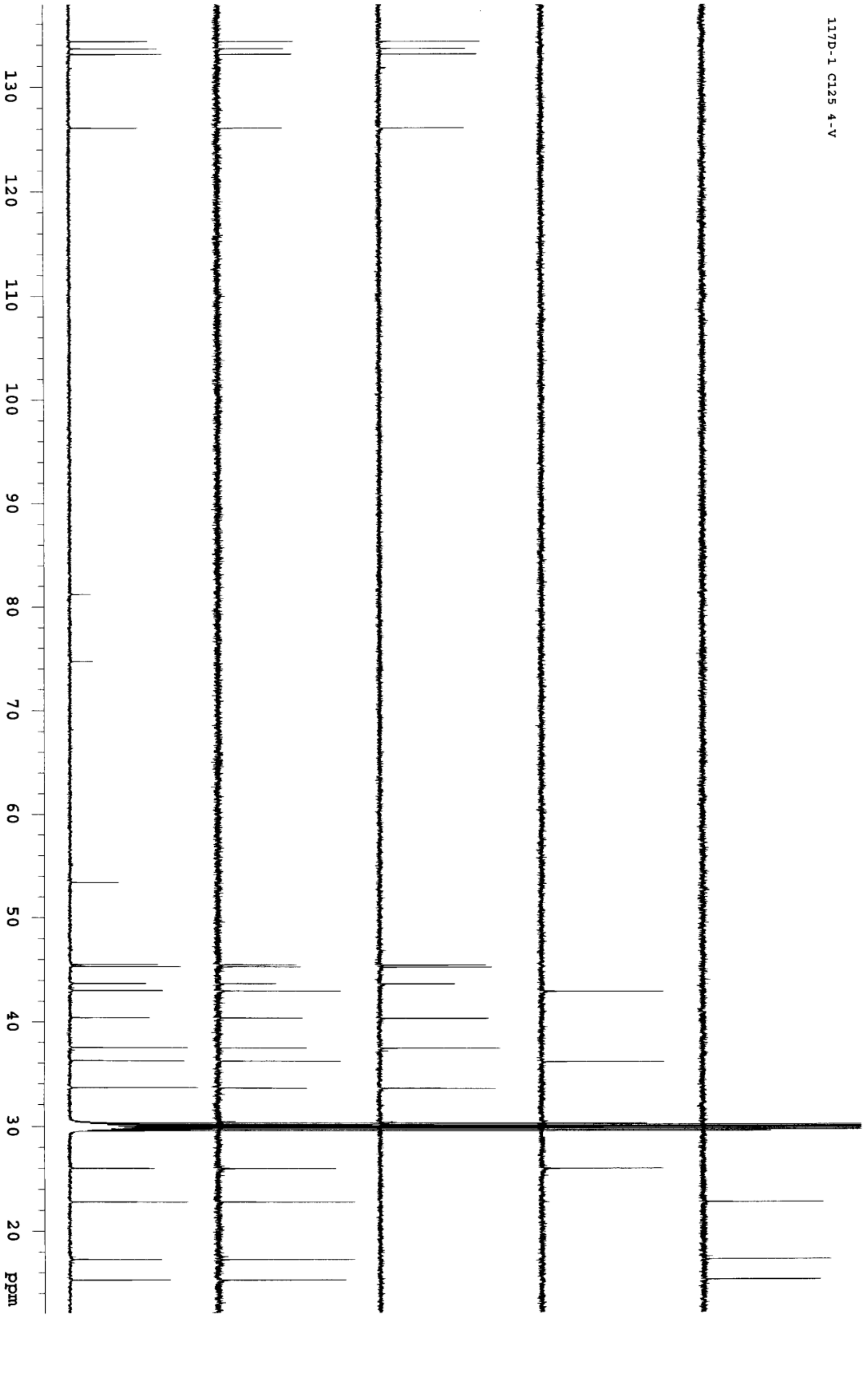
117D-1 C125 4-V
130
120
110
100
90
80
70
60
50
40
30
20
ppm

Figure S14 ^{1}H-^{1}H COSY of 3

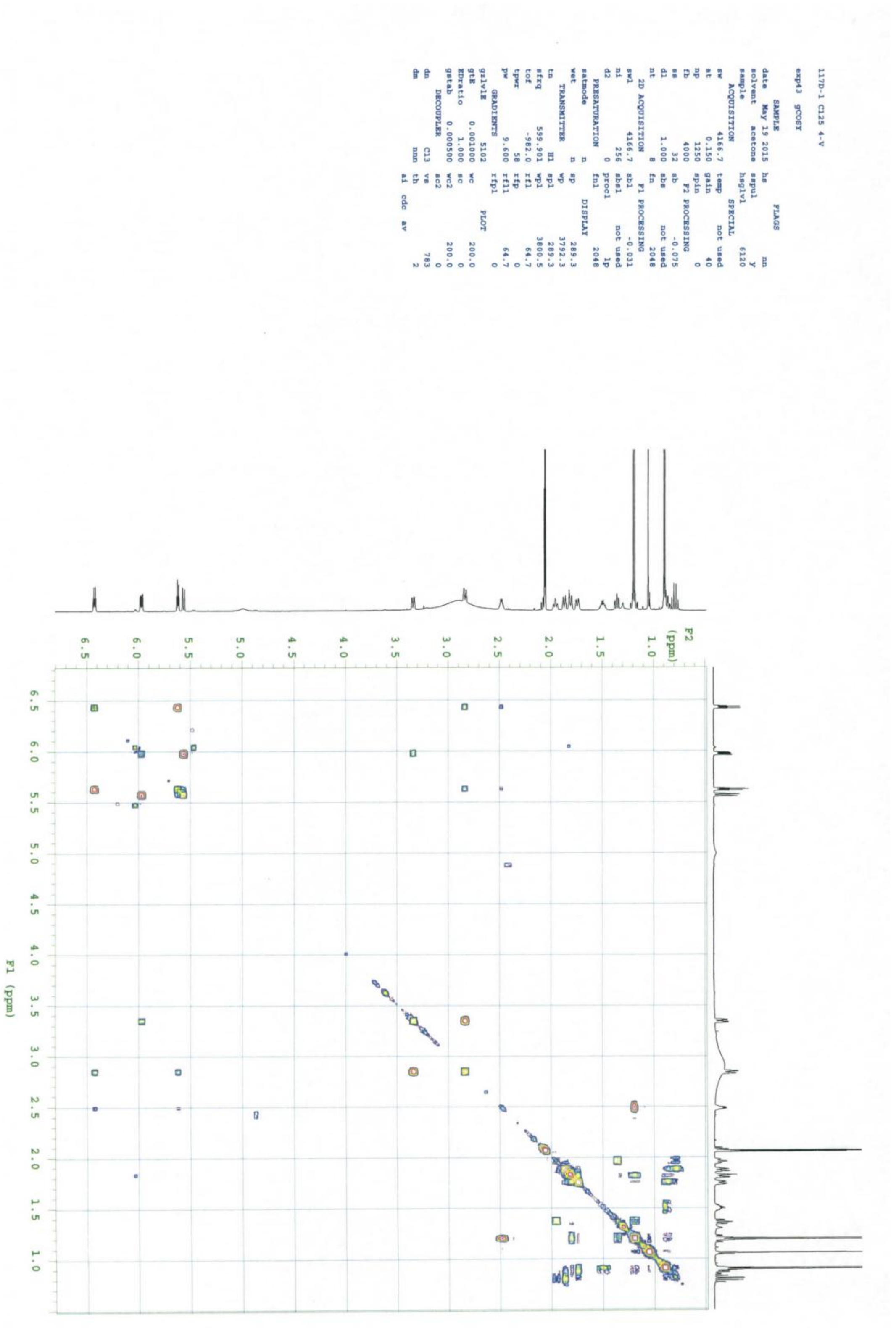

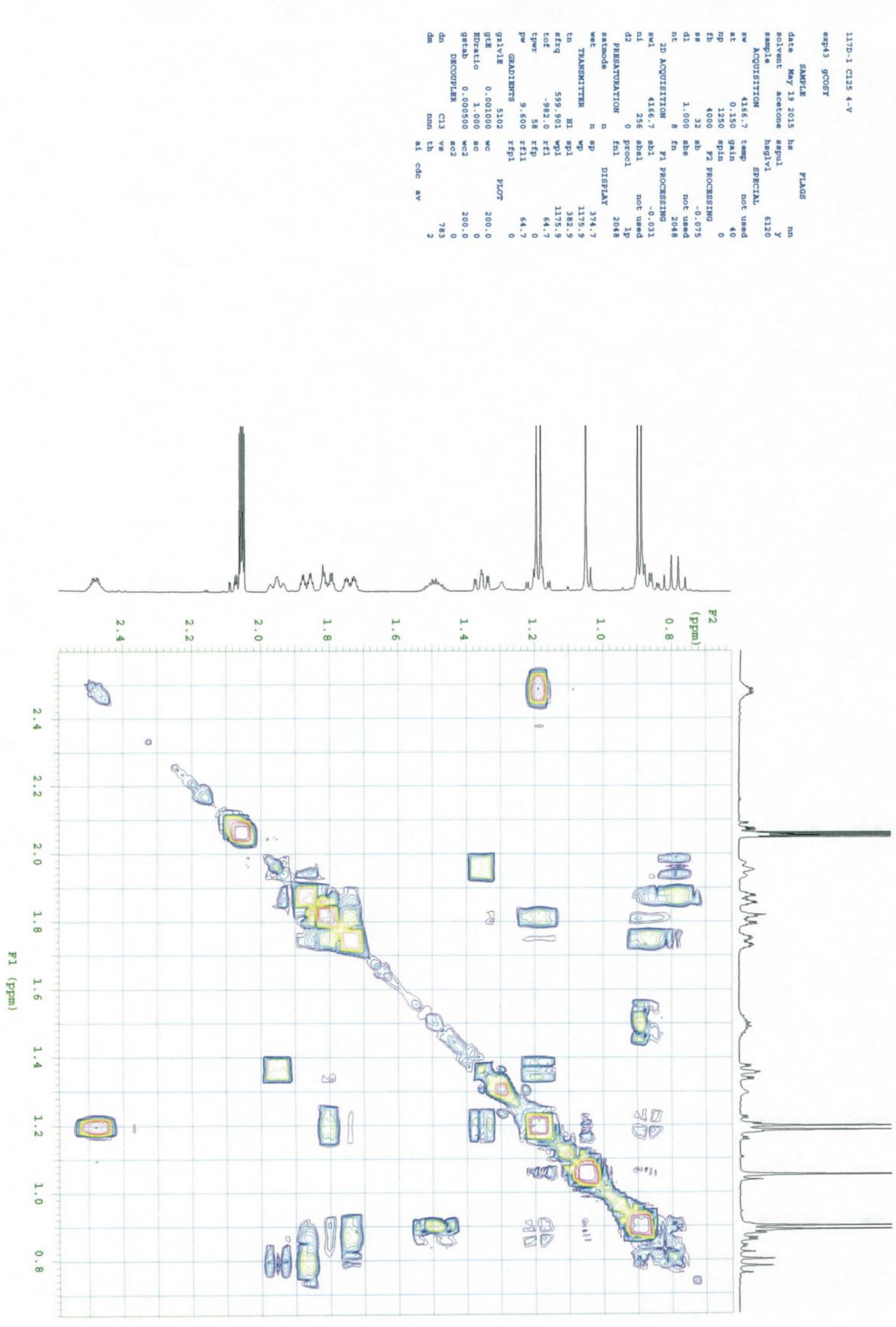

117D-1 C125 4-V
exp43 gCOSY
SAMPLE
date May 19 2015
solvent acetone
sample
ACQUISITION
sw 4166.7
at 0.150
np 1250
fb 4000
ss 32
d1 1.000
nt 8
2D ACQUISITION
sw1 4166.7
ni 256
d2 0
PRESATURATION
satmode n
wet n
TRANSMITTER
tn H1
sfrq 599.901
tof -982.0
tpwr 58
pw 9.600
GRADIENTS
gzlvlE 5102
gtE 0.001000
EDratio 1.000
gstab 0.000500
DECOUPLER
dn C13
dm nnn
FLAGS
hs nn
sspul y
hsglvl 6120
SPECIAL
temp not used
gain 40
spin 0
F2 PROCESSING
sb -0.075
sbs not used
fn 2048
F1 PROCESSING
sb1 -0.031
sbs1 not used
proc1 lp
fn1 2048
DISPLAY
sp 374.7
wp 1175.9
sp1 382.9
wp1 1175.9
rfl 64.7
rfp 0
rfl1 64.7
rfp1 0
PLOT
wc 200.0
sc 0
wc2 200.0
sc2 0
vs 783
th 2
ai cdc av
F2 (ppm)
0.8
1.0
1.2
1.4
1.6
1.8
2.0
2.2
2.4
F1 (ppm)

Figure S15　NOESY of 3

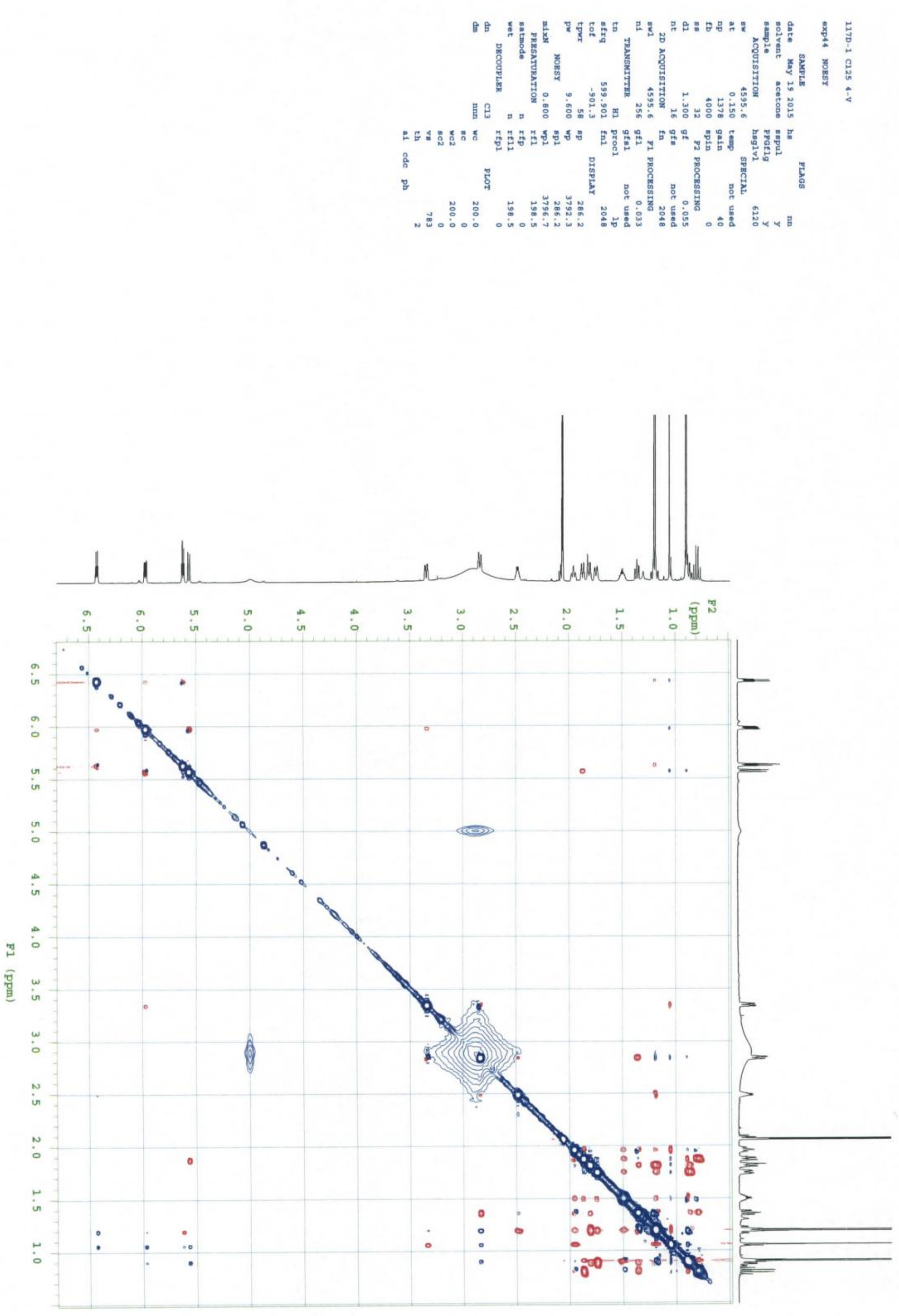

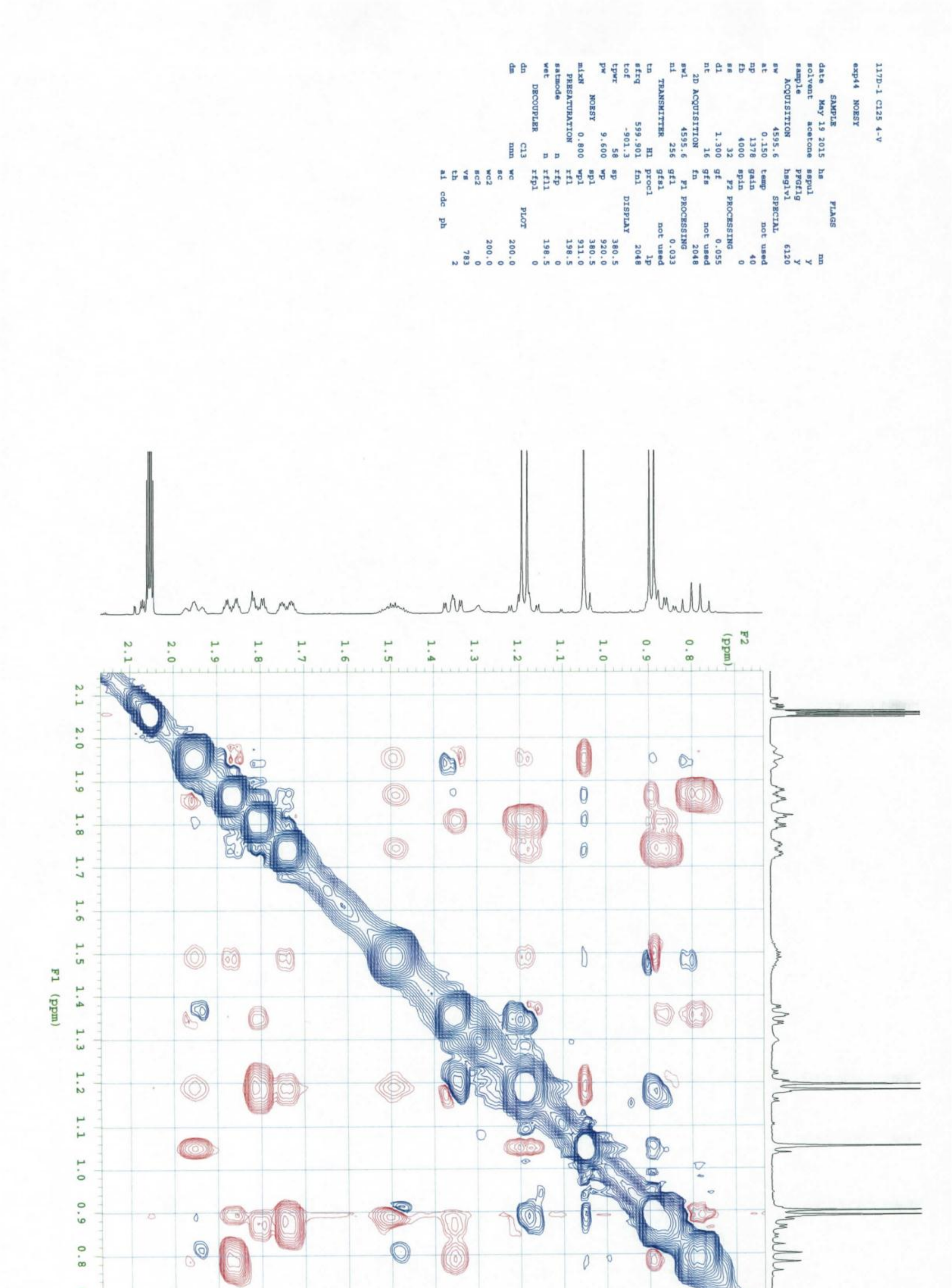
117D-1 C125 4-V
exp44 NOESY
F2 (ppm)
F1 (ppm)

Figure S16 HMQC of 3

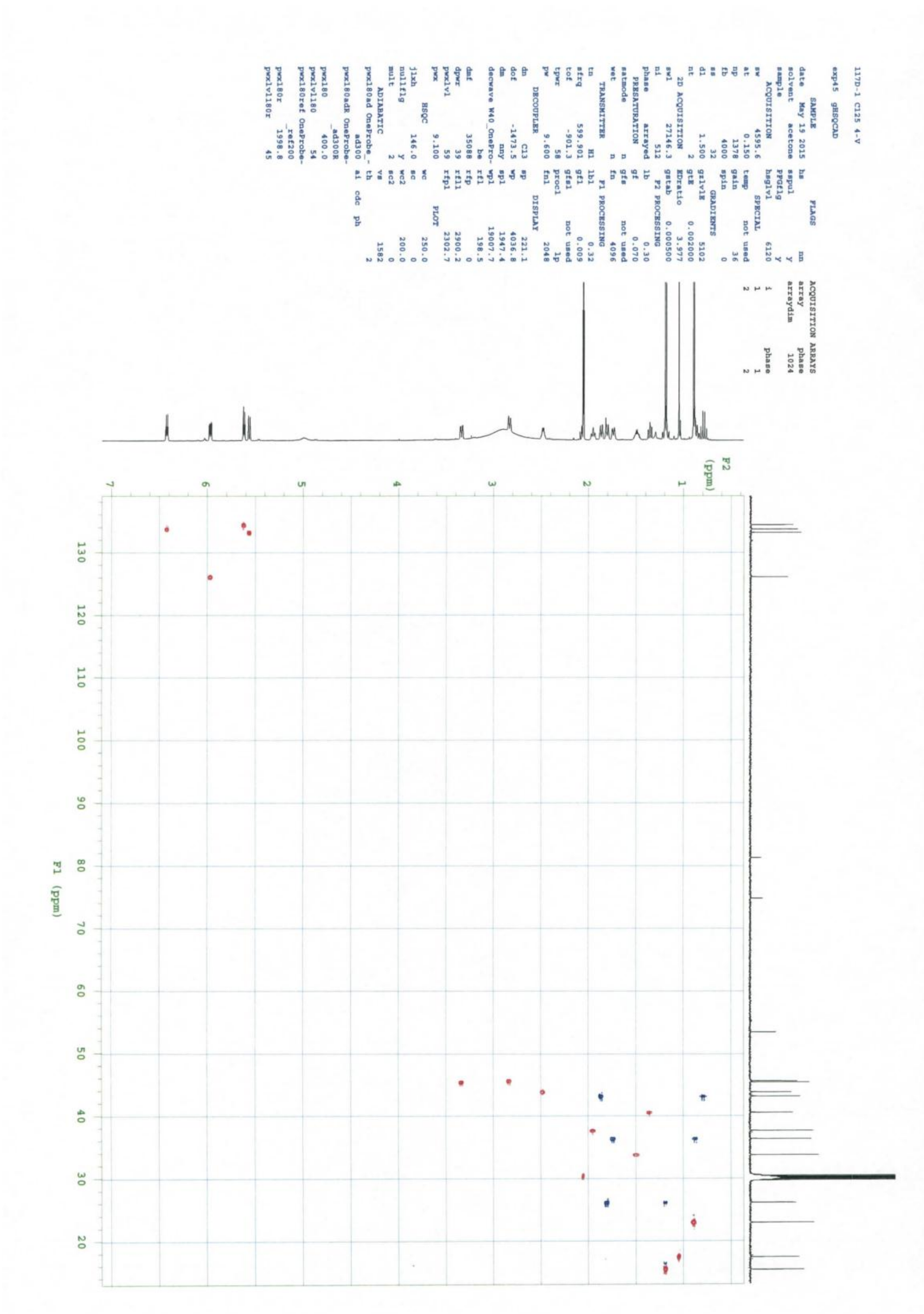

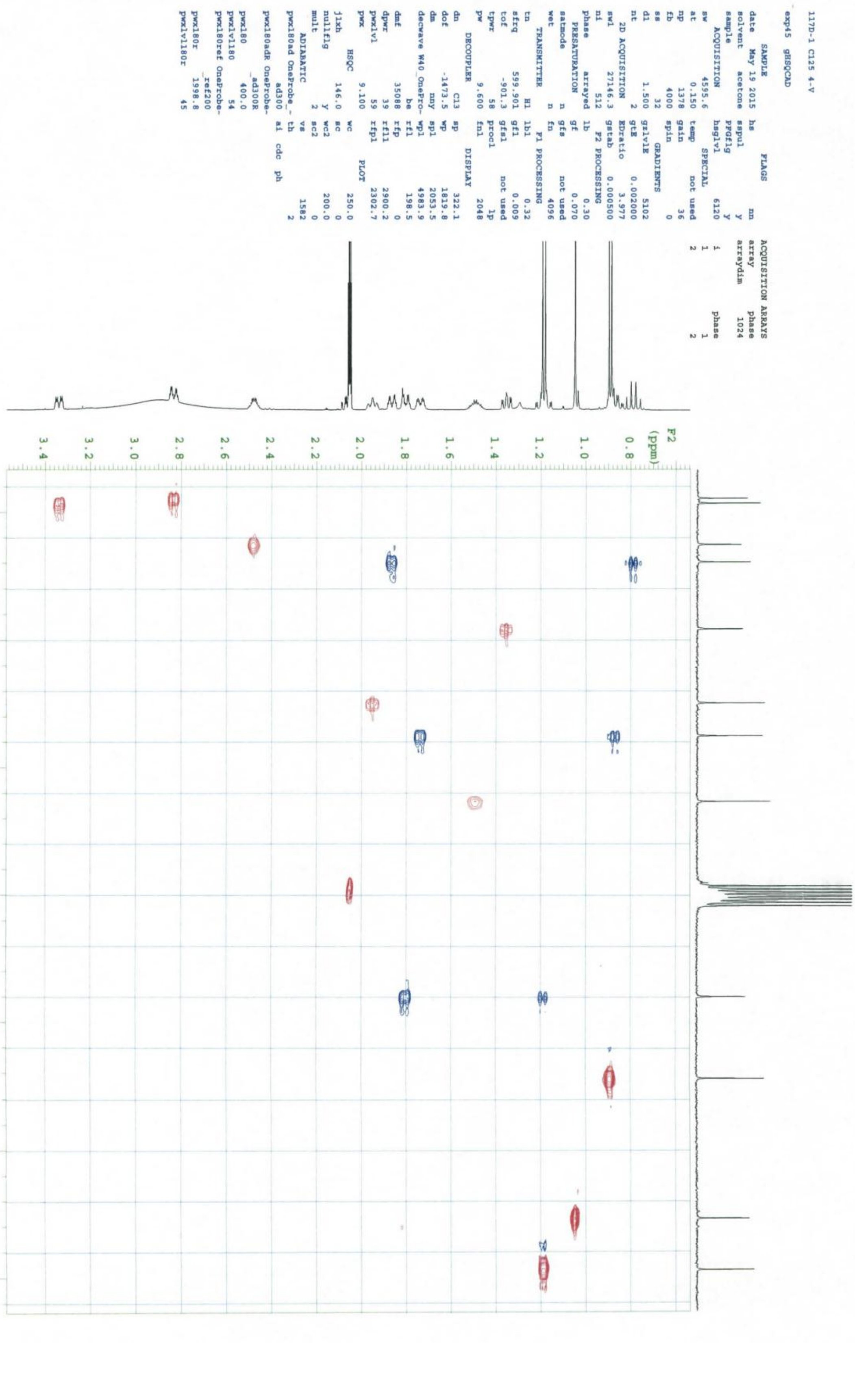

117D-1 C125 4-V
exp45 gHSQCAD
F2 (ppm)
F1 (ppm)

Figure S17   HMBC of 3

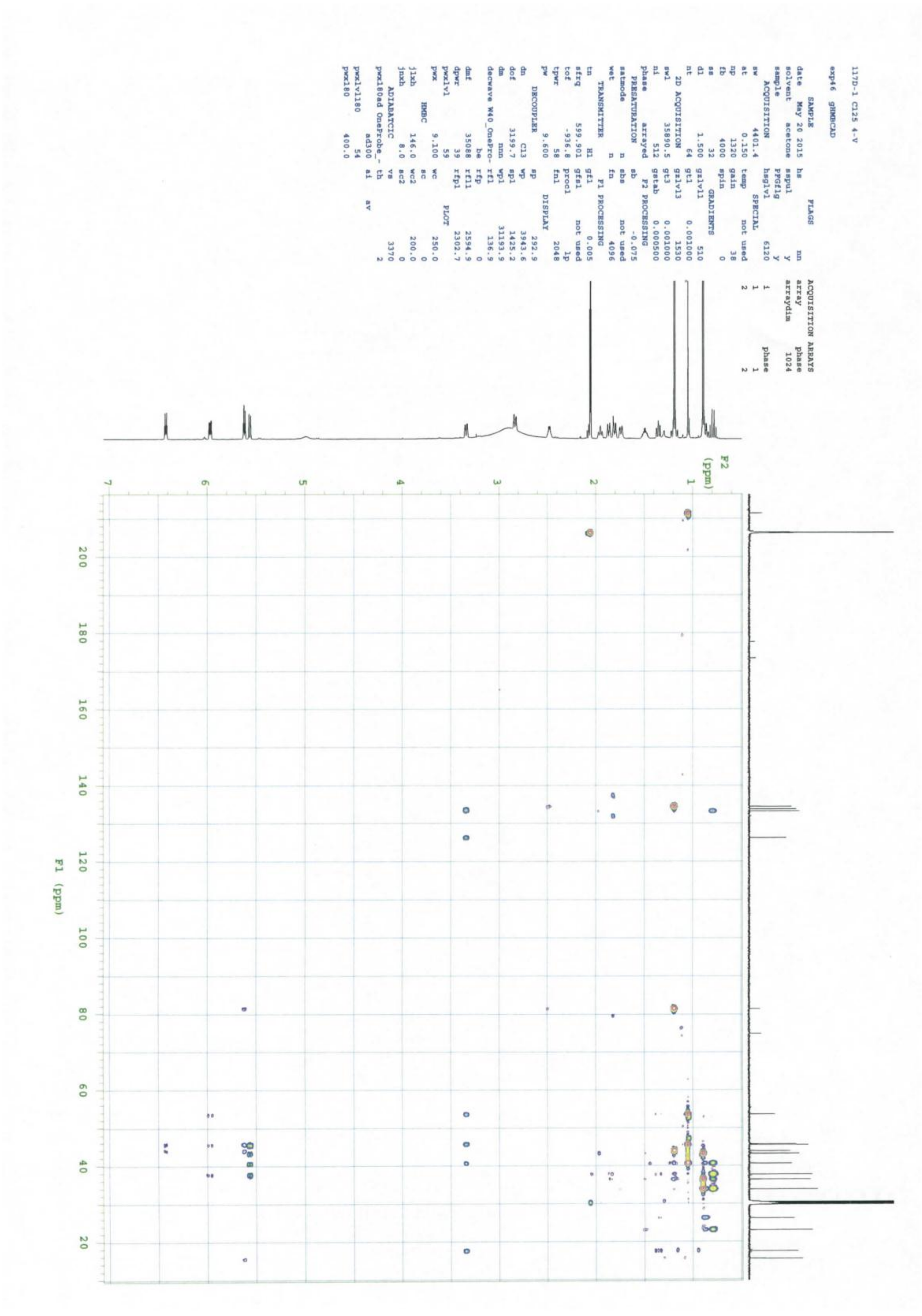

117D-1 C125 4-V

exp46 gHMBCAD

SAMPLE		FLAGS		ACQUISITION ARRAYS	
date	May 20 2015	hs	nn	array	phase
solvent	acetone	sspul	y	arraydim	1024
sample		PFGflg	y		
ACQUISITION		hsglvl	6120	i	phase
sw	4401.4	SPECIAL		1	1
at	0.150	temp	not used	2	2
np	1320	gain	38		
fb	4000	spin	0		
ss	32	GRADIENTS			
d1	1.500	gzlvl1	510		
nt	64	gt1	0.001000		
2D ACQUISITION		gzlvl3	1530		
sw1	35890.5	gt3	0.001000		
ni	512	gstab	0.000500		
phase	arrayed	F2 PROCESSING			
PRESATURATION		sb	-0.075		
satmode	n	sbs	not used		
wet	n	fn	4096		
TRANSMITTER		F1 PROCESSING			
tn	H1	gf1	0.005		
sfrq	599.901	gfs1	not used		
tof	-936.8	procl	lp		
tpwr	58	fn1	2048		
pw	9.600	DISPLAY			
DECOUPLER		sp	378.9		
dn	C13	wp	906.9		
dof	3199.7	sp1	1950.9		
dm	nnn	wp1	6589.3		
decwave	W40_OnePro-be	rfl	136.9		
		rfp	0		
dmf	35088	rfl1	2594.9		
dpwr	39	rfp1	2302.7		
pwxlvl	59	PLOT			
pwx	9.100	wc	250.0		
HMBC		sc	0		
j1xh	146.0	wc2	200.0		
jnxh	8.0	sc2	0		
ADIABATCIC		vs	3370		
pwx180ad	OneProbe_-ad300	th	2		
		ai	av		
pwxlvl180	54				
pwx180	400.0				

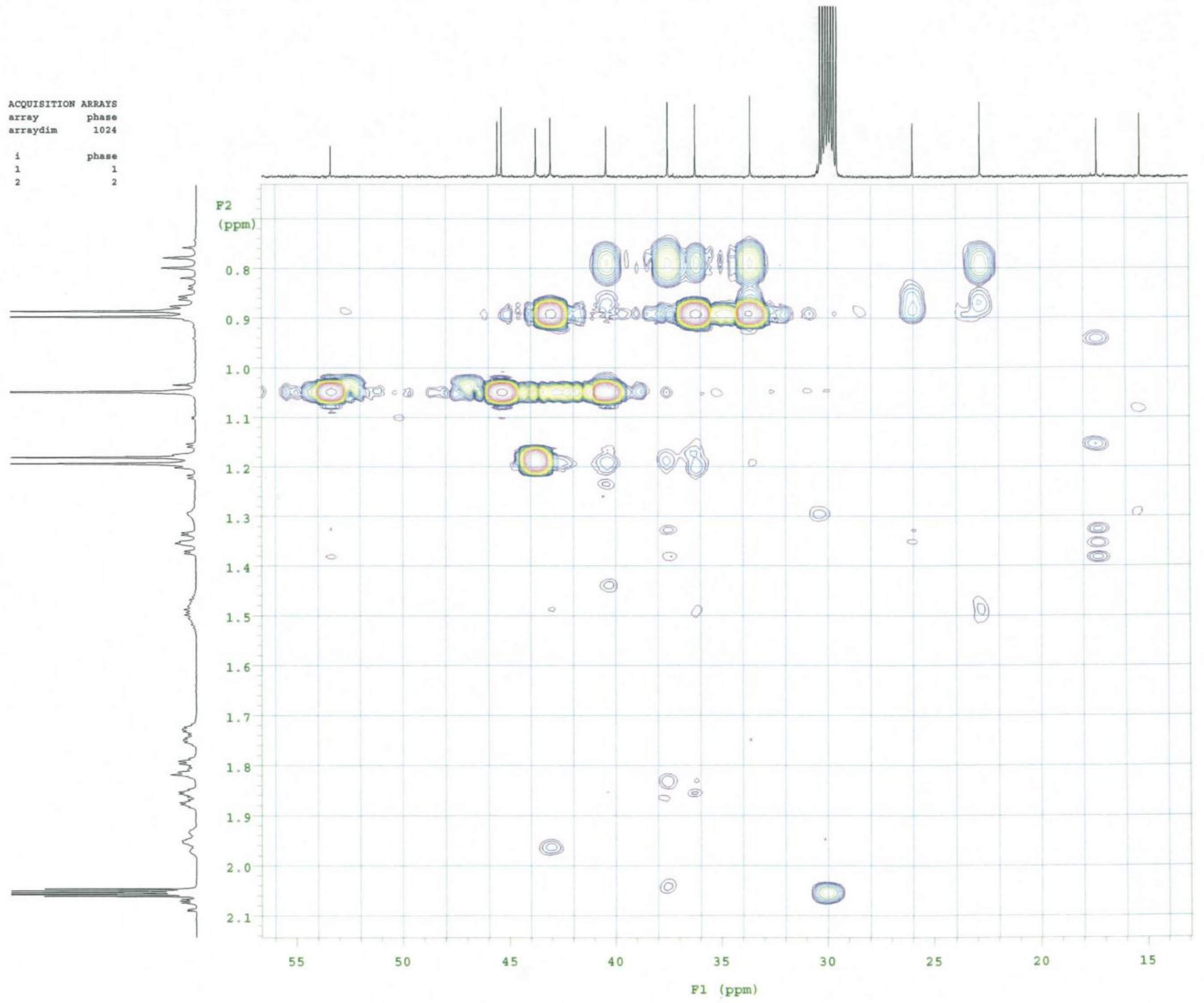

```
117D-1 C125 4-V

exp46  gHMBCAD

      SAMPLE              FLAGS            ACQUISITION ARRAYS
date   May 20 2015  hs              nn   array         phase
solvent    acetone  sspul            y   arraydim       1024
sample              PFGflg           y
   ACQUISITION      hsglvl        6120   i             phase
sw          4401.4        SPECIAL        1                 1
at           0.150  temp      not used   2                 2
np            1320  gain            38
fb            4000  spin             0
ss              32       GRADIENTS
d1           1.500  gzlvl1         510
nt              64  gt1       0.001000
  2D ACQUISITION    gzlvl3        1530
sw1        35890.5  gt3       0.001000
ni             512  gstab     0.000500
phase      arrayed     F2 PROCESSING
  PRESATURATION     sb          -0.075
satmode          n  sbs       not used
wet              n  fn            4096
   TRANSMITTER         F1 PROCESSING
tn              H1  gf1          0.005
sfrq       599.901  gfs1      not used
tof         -936.8  proc1           lp
tpwr            58  fn1           2048
pw           9.600       DISPLAY
   DECOUPLER        sp           314.4
dn             C13  wp          3896.4
dof         3199.7  sp1         2021.0
dm             nnn  wp1         6519.2
decwave W40_OnePro- rf1          136.9
                be  rfp              0
dmf          35088  rfl1        2594.9
dpwr            39  rfp1        2302.7
pwxlvl          59        PLOT
pwx          9.100  wc           250.0
      HMBC          sc               0
j1xh         146.0  wc2          200.0
jnxh           8.0  sc2              0
   ADIABATCIC       vs            3370
pwx180ad OneProbe_- th               2
             ad300  ai    av
pwxlvl180       54
pwx180       400.0
```

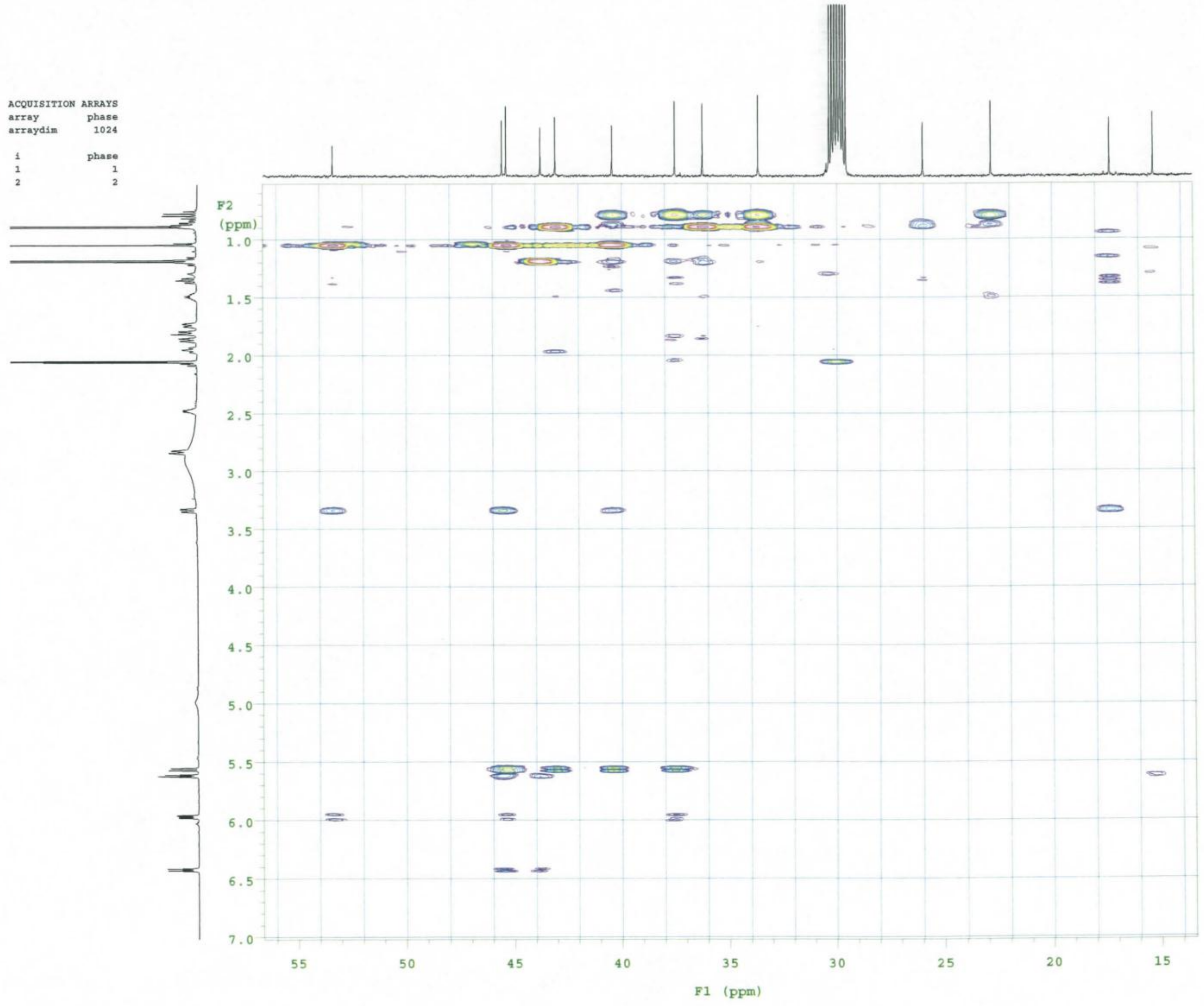

Figure S18 FABMS of 3

```
[ Elemental Composition ]  3                                  Page: 1
Data  : 1506059                  Date : 10-Jun-2015 15:54
Sample: 117D-1 C125-4-V
Note : Matrix; G
Inlet : Direct                   Ion Mode : FAB+
RT : 8.41 min                    Scan#: (27,32)
Elements : C 30/17, H 32/22, N 3/0, O 6/0, Na 1/0
Mass Tolerance      : 20ppm, 1mmu if m/z > 50
Unsaturation (U.S.) : -1.0 - 50.0

Observed m/z Int%   Err[ppm / mmu]    U.S.  Composition
  392.1839    37.4     +0.4 /  +0.2     9.5  C 22 H 27 N O 4 Na
```

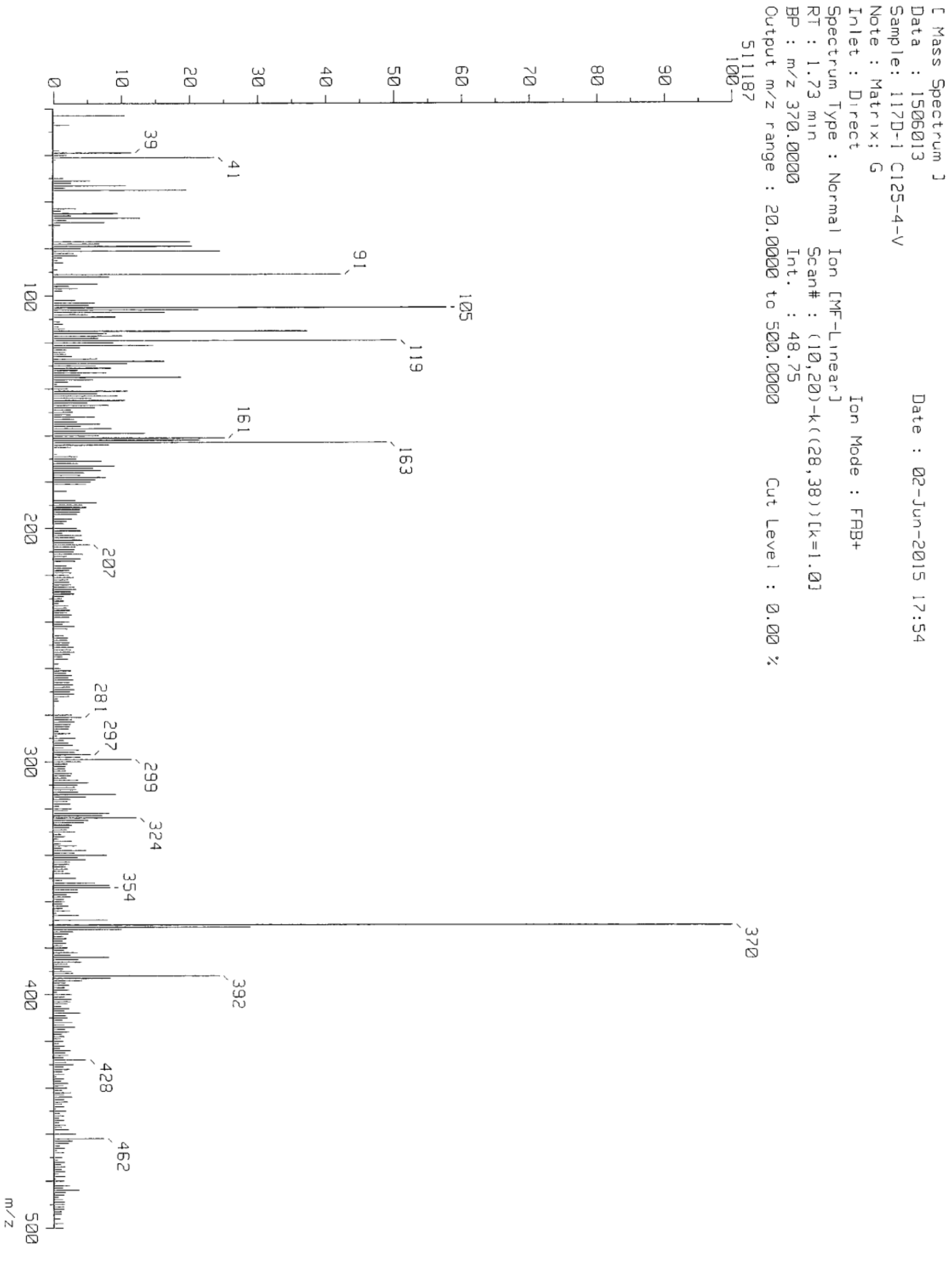
[Mass Spectrum]
Data : 1506013
Date : 02-Jun-2015 17:54
Sample: 117D-1 C125-4-V
Note : Matrix; G
Inlet : Direct
Ion Mode : FAB+
Spectrum Type : Normal Ion [MF-Linear]
RT : 1.73 min
Scan# : (10,20)-k((28,38))[k=1.0]
BP : m/z 370.0000
Int. : 48.75
Output m/z range : 20.0000 to 500.0000
Cut Level : 0.00 %
511187
m/z
