# Supplementary material for: Altercrasins A–E, Decalin Derivatives, from a Sea-Urchin-Derived Alternaria sp.: Isolation and Structural Analysis Including Stereochemistry
Source: Mar Drugs. 2019 Apr 11;17(4):218. doi: 10.3390/md17040218 (PMC6521173; doi:10.3390/md17040218)

Figure S19 ^{1}H **and** ^{13}C **NMR spectra in acetone-*d*$_6$ of 4**

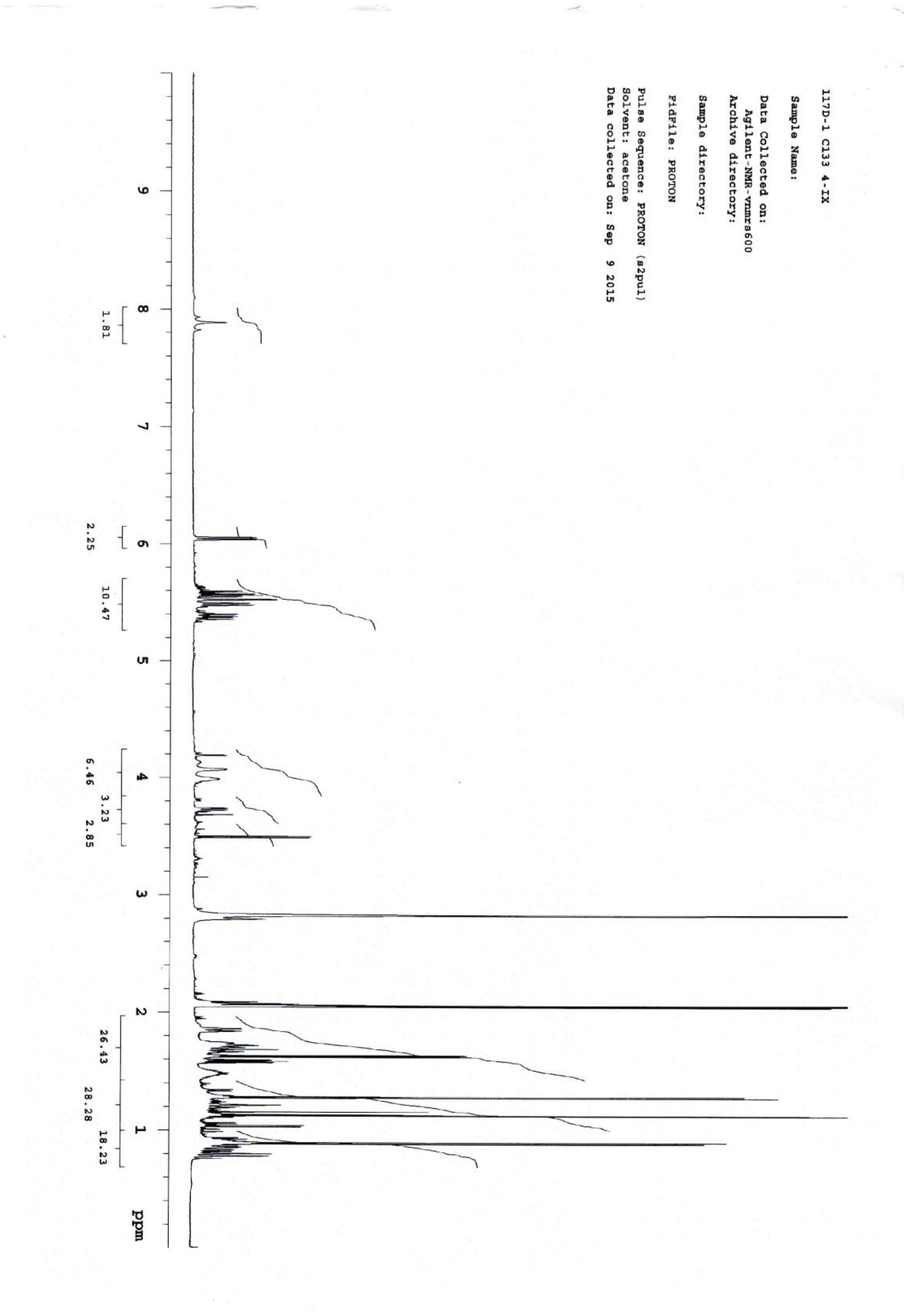

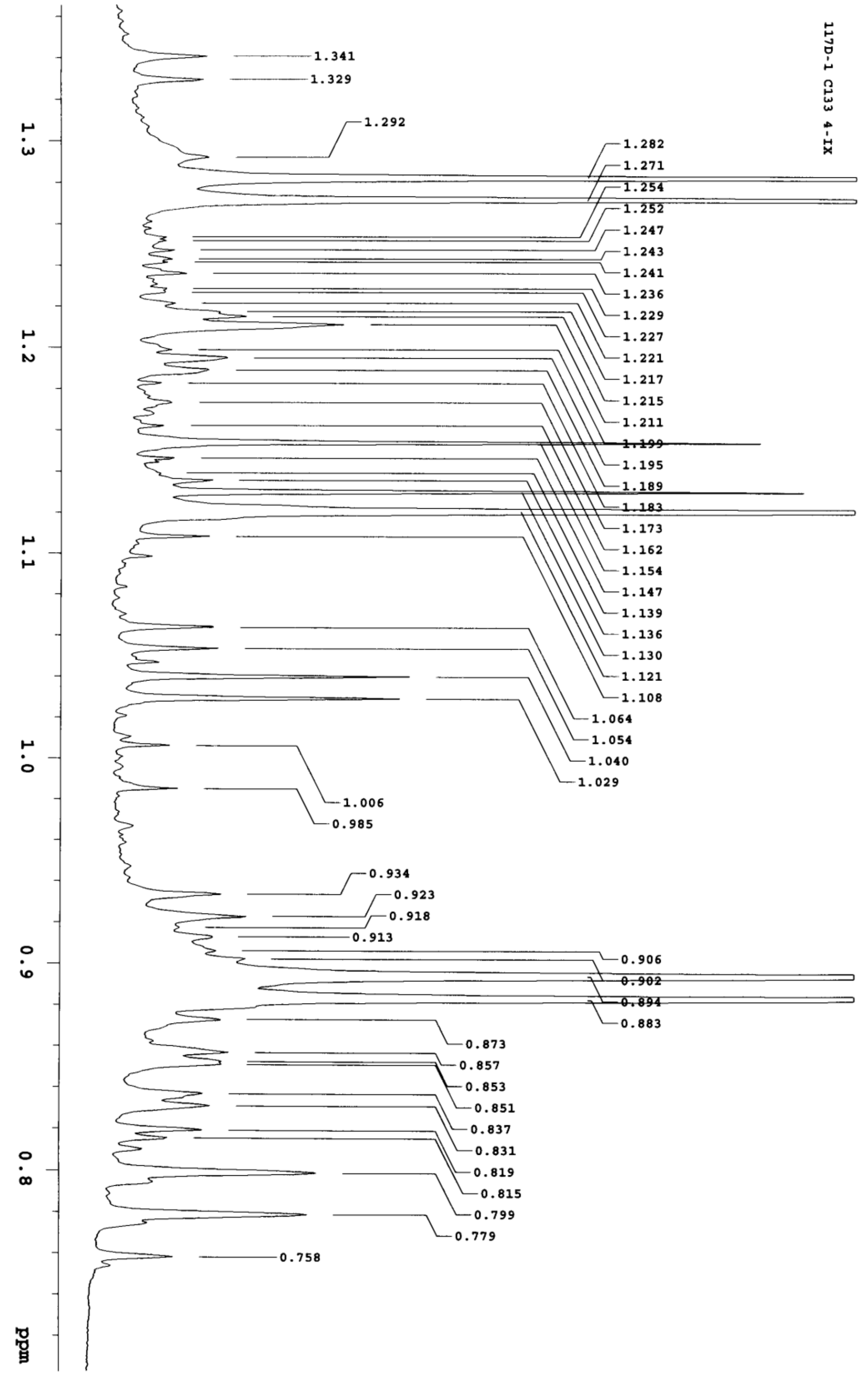
117D-1 C133 4-IX
1.341
1.329
1.292
1.282
1.271
1.254
1.252
1.247
1.243
1.241
1.236
1.229
1.227
1.221
1.217
1.215
1.211
1.199
1.195
1.189
1.183
1.173
1.162
1.154
1.147
1.139
1.136
1.130
1.121
1.108
1.064
1.054
1.040
1.029
1.006
0.985
0.934
0.923
0.918
0.913
0.906
0.902
0.894
0.883
0.873
0.857
0.853
0.851
0.837
0.831
0.819
0.815
0.799
0.779
0.758
1.3
1.2
1.1
1.0
0.9
0.8
ppm

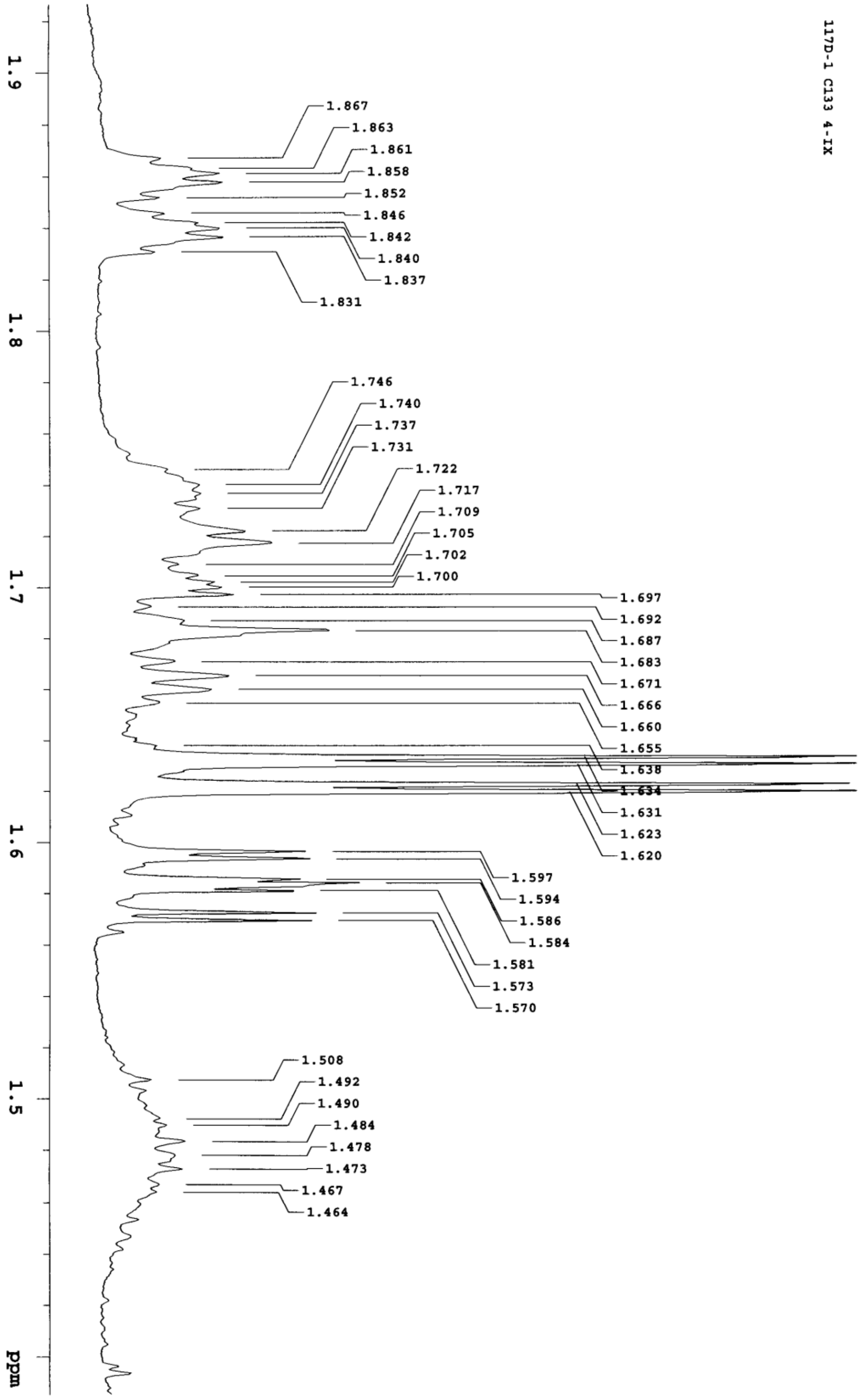
117D-1 C133 4-IX
1.867
1.863
1.861
1.858
1.852
1.846
1.842
1.840
1.837
1.831
1.746
1.740
1.737
1.731
1.722
1.717
1.709
1.705
1.702
1.700
1.697
1.692
1.687
1.683
1.671
1.666
1.660
1.655
1.638
1.634
1.631
1.623
1.620
1.597
1.594
1.586
1.584
1.581
1.573
1.570
1.508
1.492
1.490
1.484
1.478
1.473
1.467
1.464
1.9
1.8
1.7
1.6
1.5
ppm

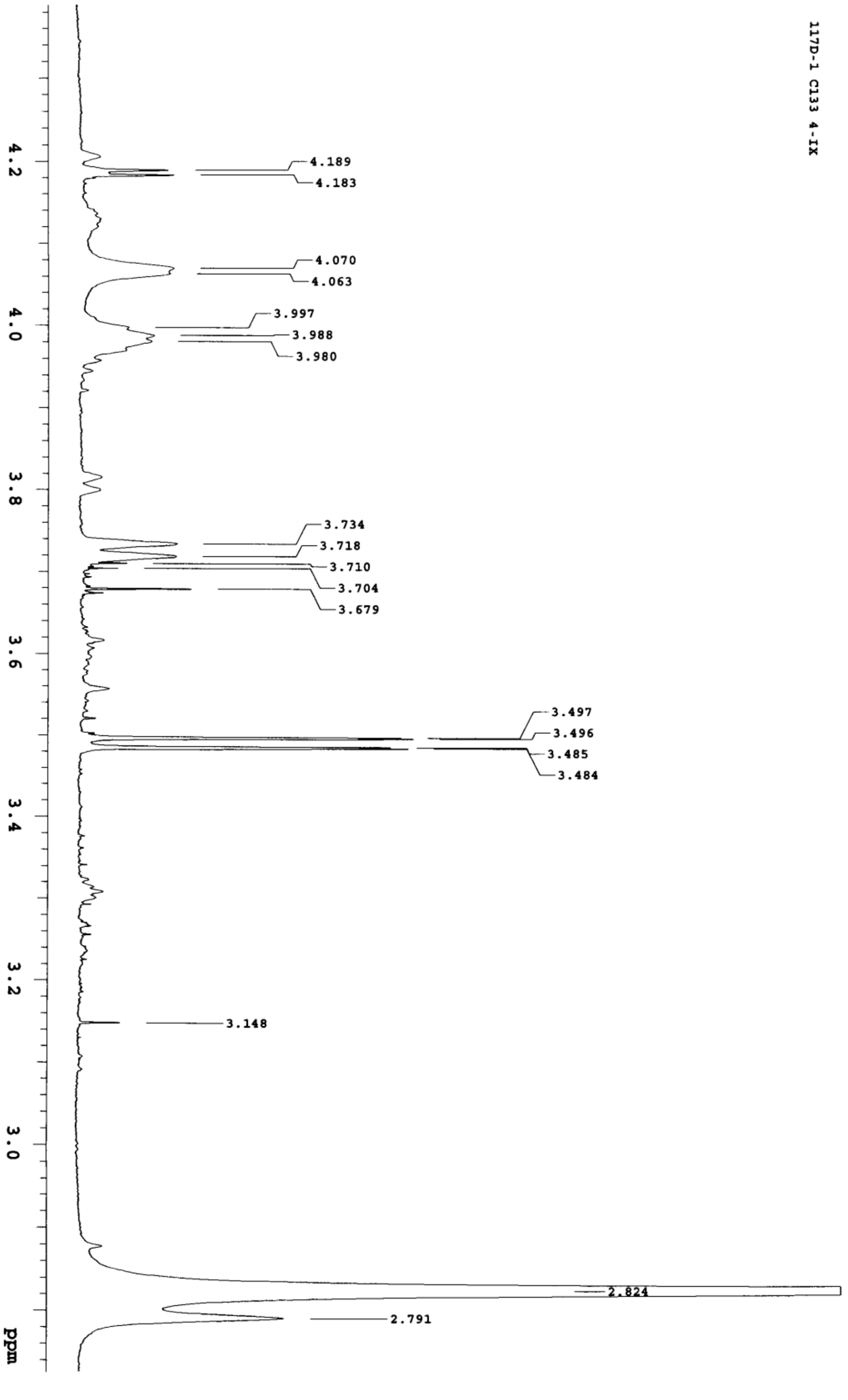
117D-1 C133 4-IX
4.189
4.183
4.070
4.063
3.997
3.988
3.980
3.734
3.718
3.710
3.704
3.679
3.497
3.496
3.485
3.484
3.148
2.824
2.791
4.2
4.0
3.8
3.6
3.4
3.2
3.0
ppm

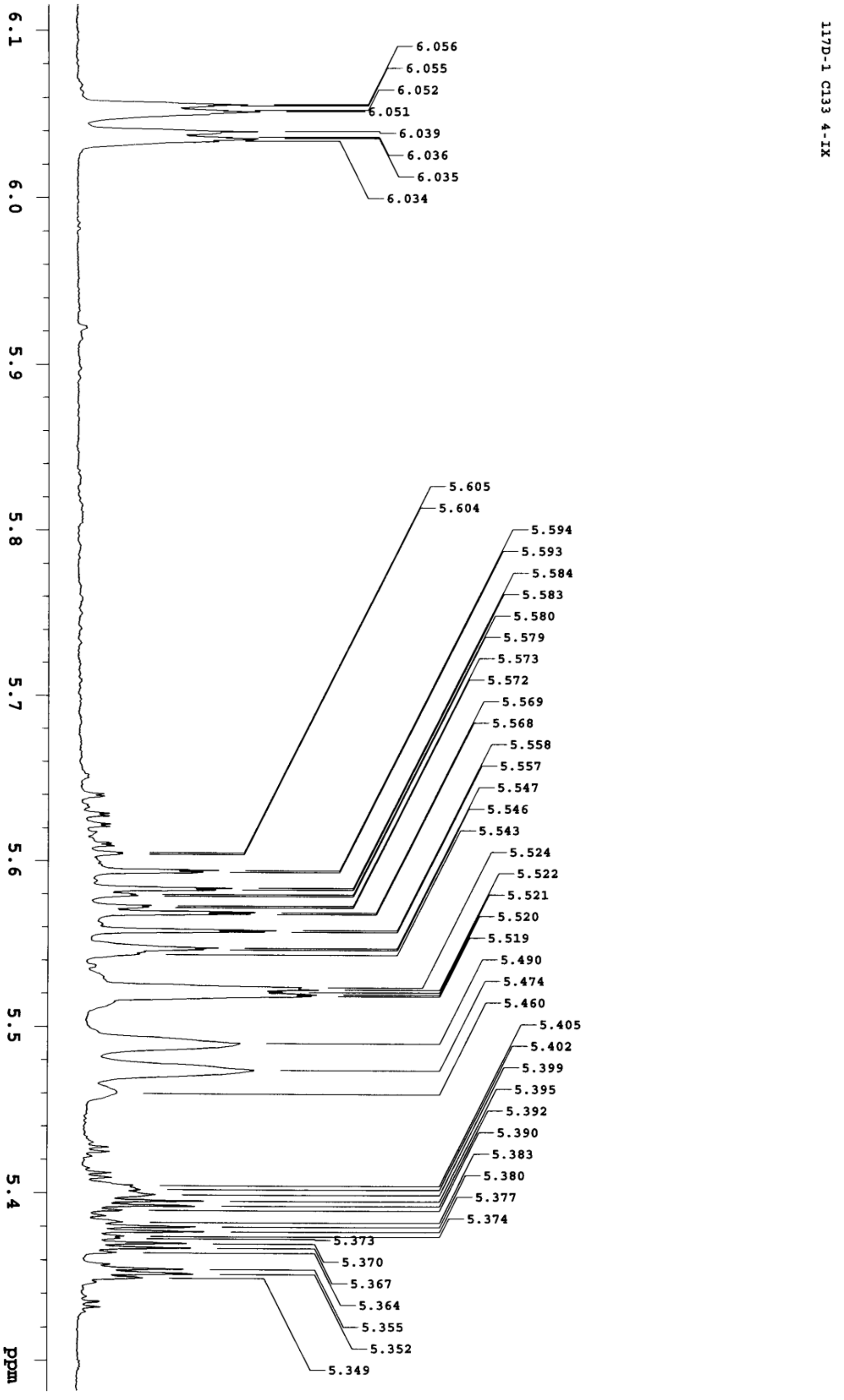
117D-1 C133 4-IX
6.1
6.0
5.9
5.8
5.7
5.6
5.5
5.4
ppm
6.056
6.055
6.052
6.051
6.039
6.036
6.035
6.034
5.605
5.604
5.594
5.593
5.584
5.583
5.580
5.579
5.573
5.572
5.569
5.568
5.558
5.557
5.547
5.546
5.543
5.524
5.522
5.521
5.520
5.519
5.490
5.474
5.460
5.405
5.402
5.399
5.395
5.392
5.390
5.383
5.380
5.377
5.374
5.373
5.370
5.367
5.364
5.355
5.352
5.349

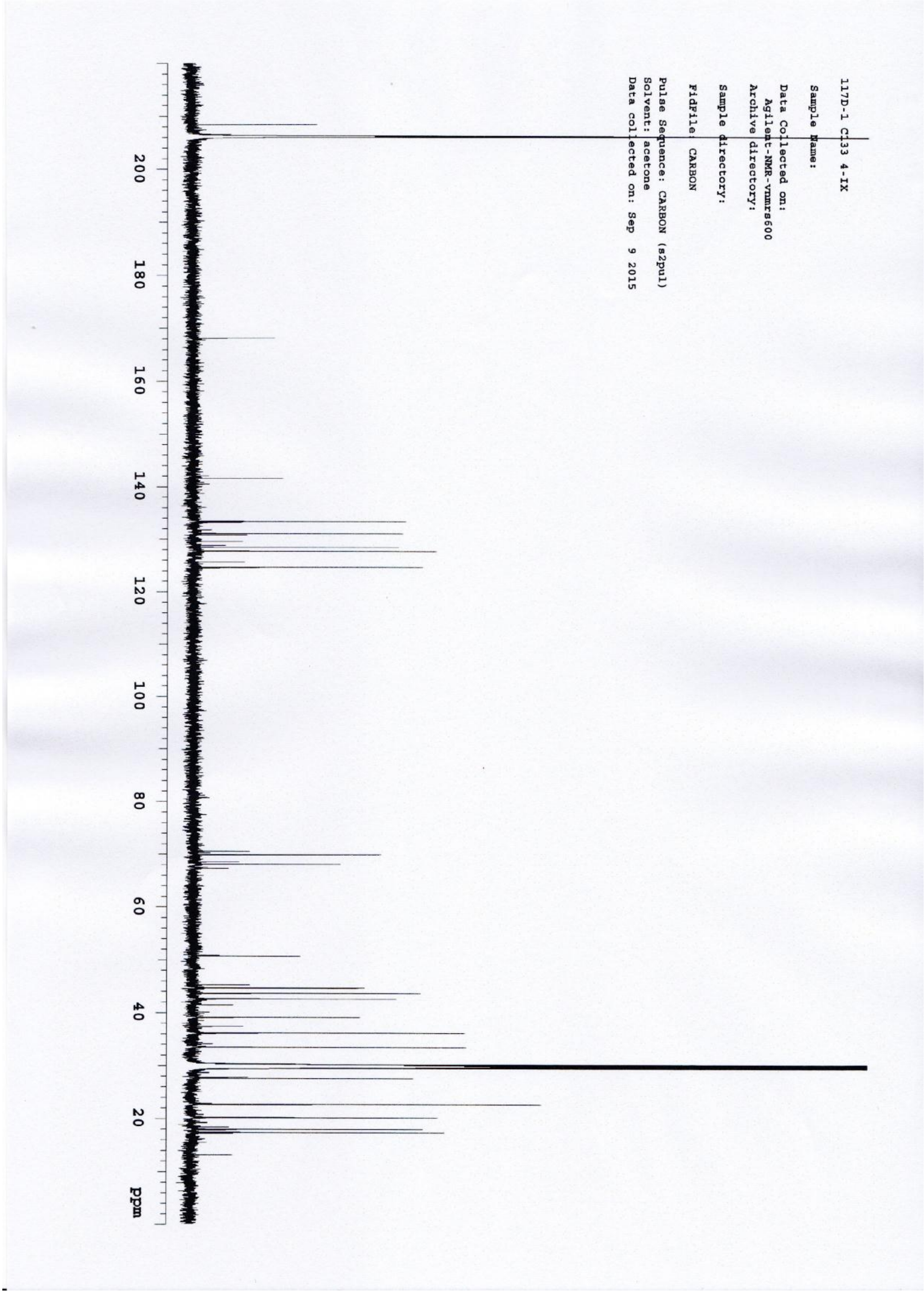

117D-1 C133 4-IX
Sample Name:
Data Collected on:
Agilent-NMR-vnmrs600
Archive directory:
Sample directory:
FidFile: CARBON
Pulse Sequence: CARBON (s2pul)
Solvent: acetone
Data collected on: Sep 9 2015
200
180
160
140
120
100
80
60
40
20
ppm

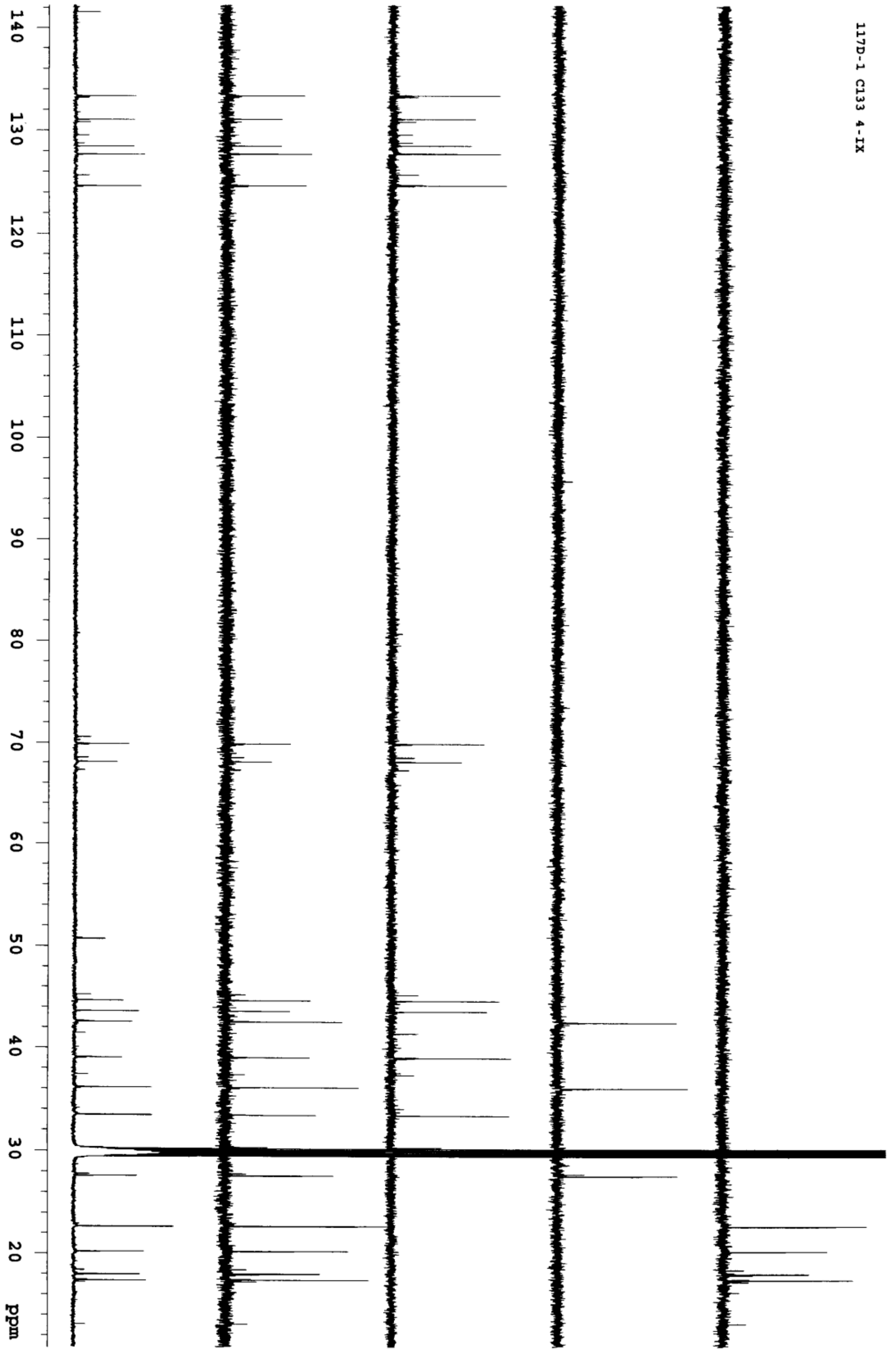
117D-1 C133 4-IX
140
130
120
110
100
90
80
70
60
50
40
30
20
ppm

Figure S20 ¹H-¹H COSY of 4

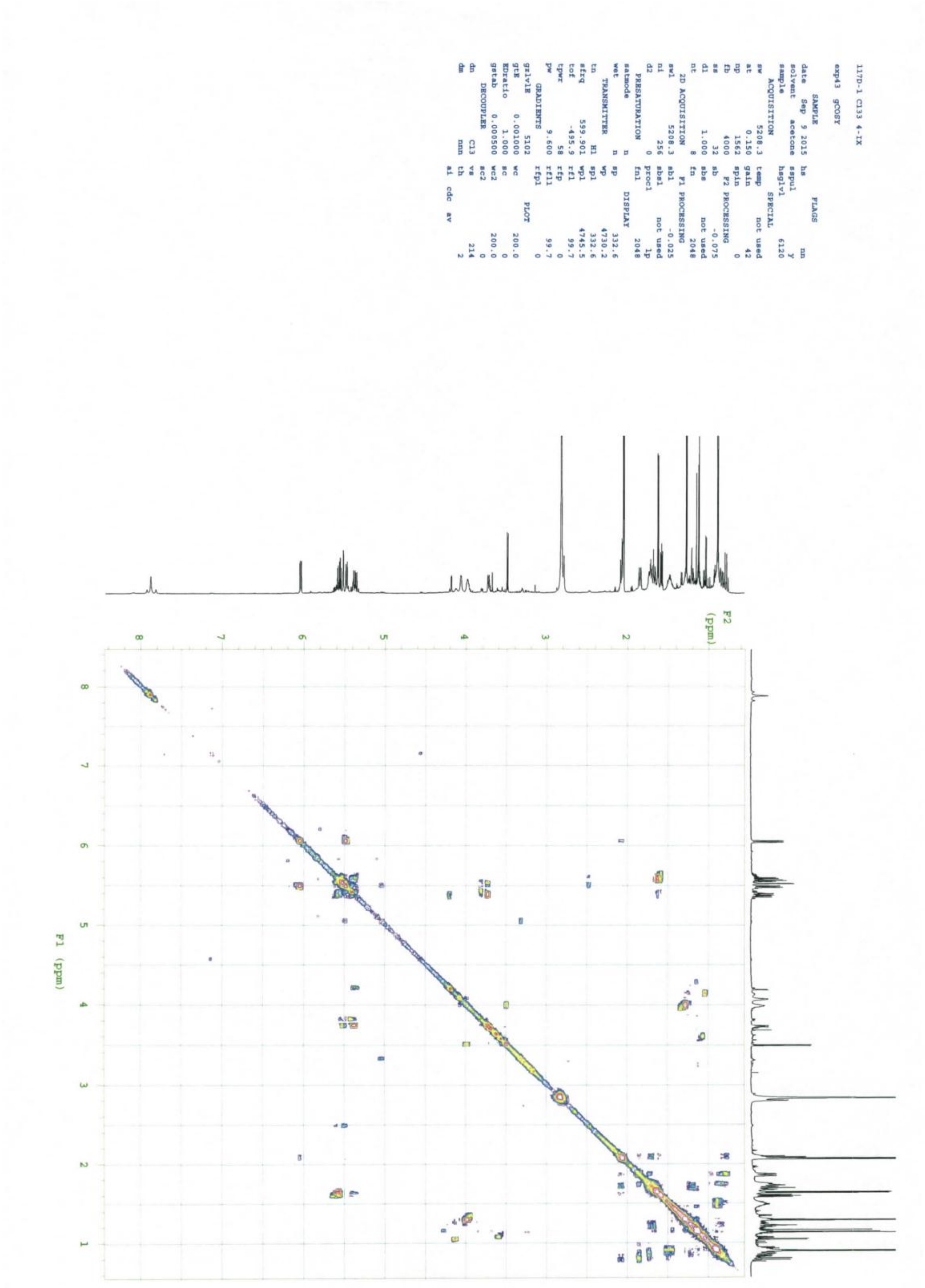

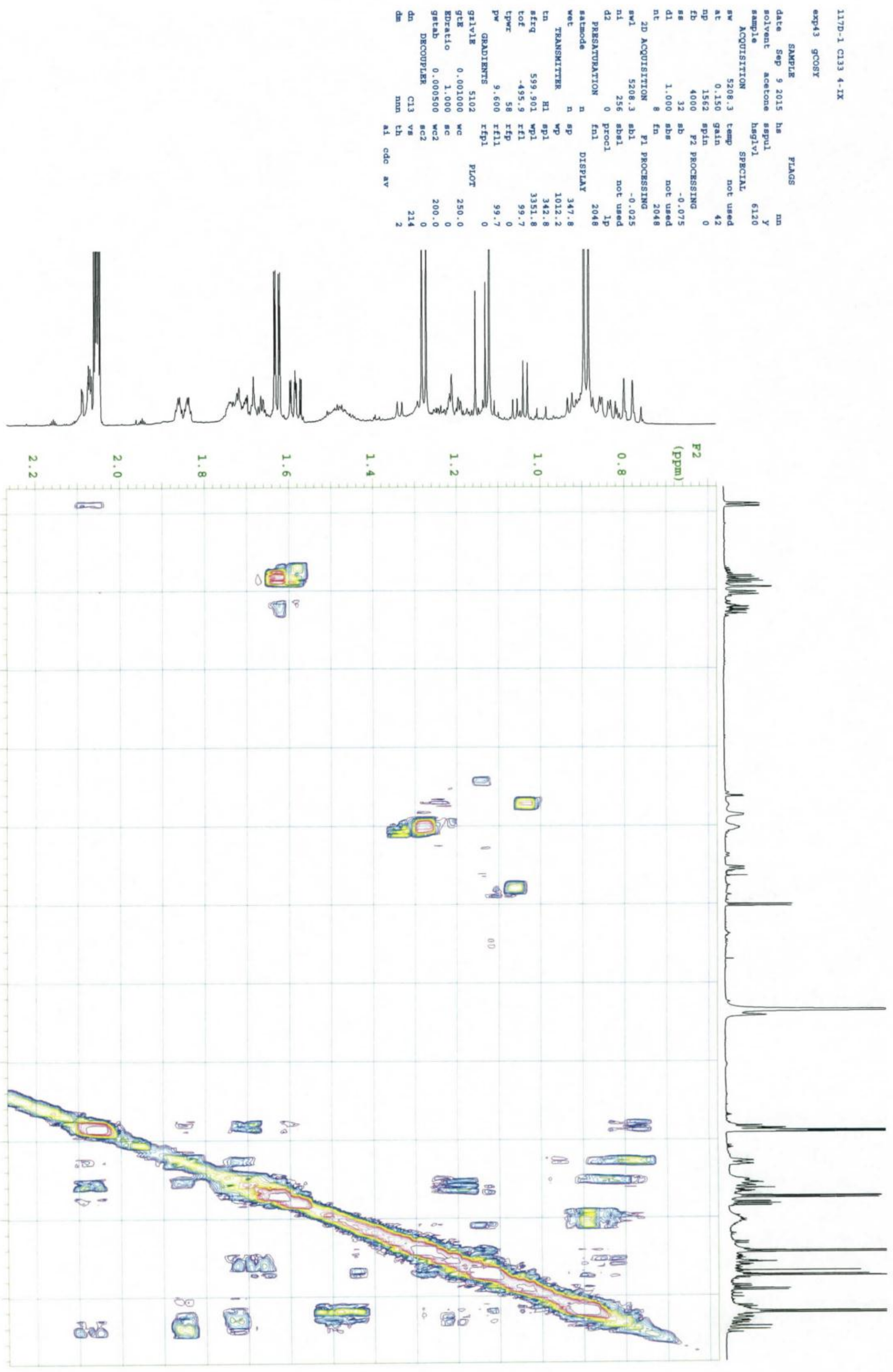
117D-1 C133 4-IX
exp43 gCOSY
SAMPLE FLAGS
date Sep 9 2015 hs nn
solvent acetone sspul y
sample hsglvl 6120
ACQUISITION SPECIAL
sw 5208.3 temp not used
at 0.150 gain 42
np 1562 spin 0
fb 4000 F2 PROCESSING
ss 32 sb -0.075
d1 1.000 sbs not used
nt 8 fn 2048
2D ACQUISITION F1 PROCESSING
sw1 5208.3 sb1 -0.025
ni 256 sbs1 not used
d2 0 procl lp
PRESATURATION fn1 2048
satmode n DISPLAY
wet n sp 347.8
TRANSMITTER wp 1012.2
tn H1 sp1 342.8
sfrq 599.901 wp1 3351.8
tof -495.9 rfl 99.7
tpwr 58 rfp 0
pw 9.600 rfl1 99.7
GRADIENTS rfp1 0
gzlvlE 5102 PLOT
gtE 0.001000 wc 250.0
EDratio 1.000 sc 0
gstab 0.000500 wc2 200.0
DECOUPLER sc2 0
dn C13 vs 214
dm nnn th 2
ai cdc av
F2 (ppm)
F1 (ppm)

Figure S21 NOESY of 4

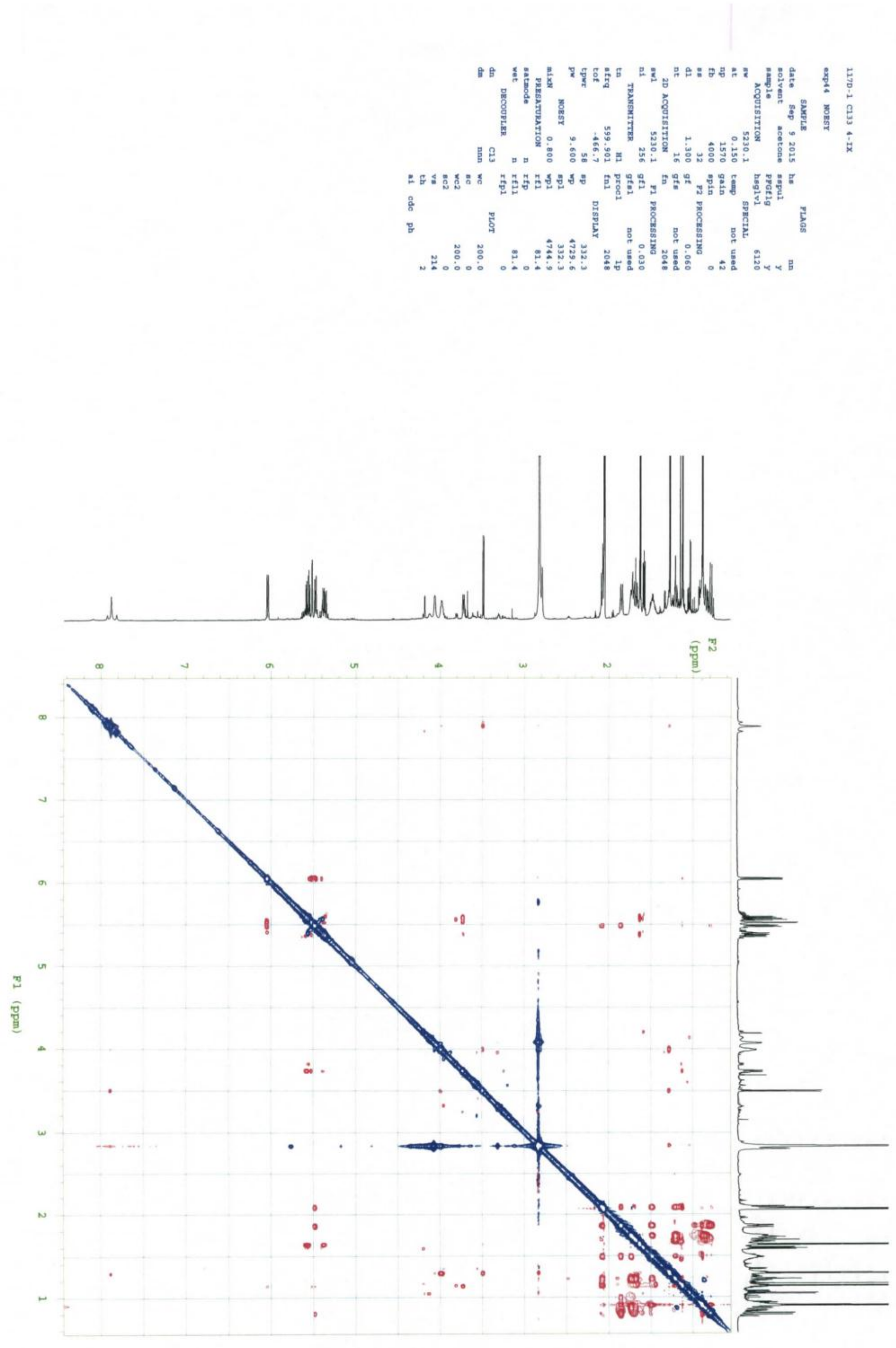

117D-1 C133 4-IX

exp44 NOESY

SAMPLE		FLAGS	
date	Sep 9 2015	hs	nn
solvent	acetone	sspul	y
sample		PFGflg	y
ACQUISITION		hsglvl	6120
sw	5230.1	SPECIAL	
at	0.150	temp	not used
np	1570	gain	42
fb	4000	spin	0
ss	32	F2 PROCESSING	
d1	1.300	gf	0.060
nt	16	gfs	not used
2D ACQUISITION		fn	2048
sw1	5230.1	F1 PROCESSING	
ni	256	gf1	0.030
TRANSMITTER		gfs1	not used
tn	H1	proc1	lp
sfrq	599.901	fn1	2048
tof	-466.7	DISPLAY	
tpwr	58	sp	388.5
pw	9.600	wp	934.7
NOESY		sp1	378.2
mixN	0.800	wp1	944.9
PRESATURATION		rfl	81.4
satmode	n	rfp	0
wet	n	rfl1	81.4
DECOUPLER		rfp1	0
dn	C13	PLOT	
dm	nnn	wc	200.0
		sc	0
		wc2	200.0
		sc2	0
		vs	214
		th	2
		ai cdc ph	

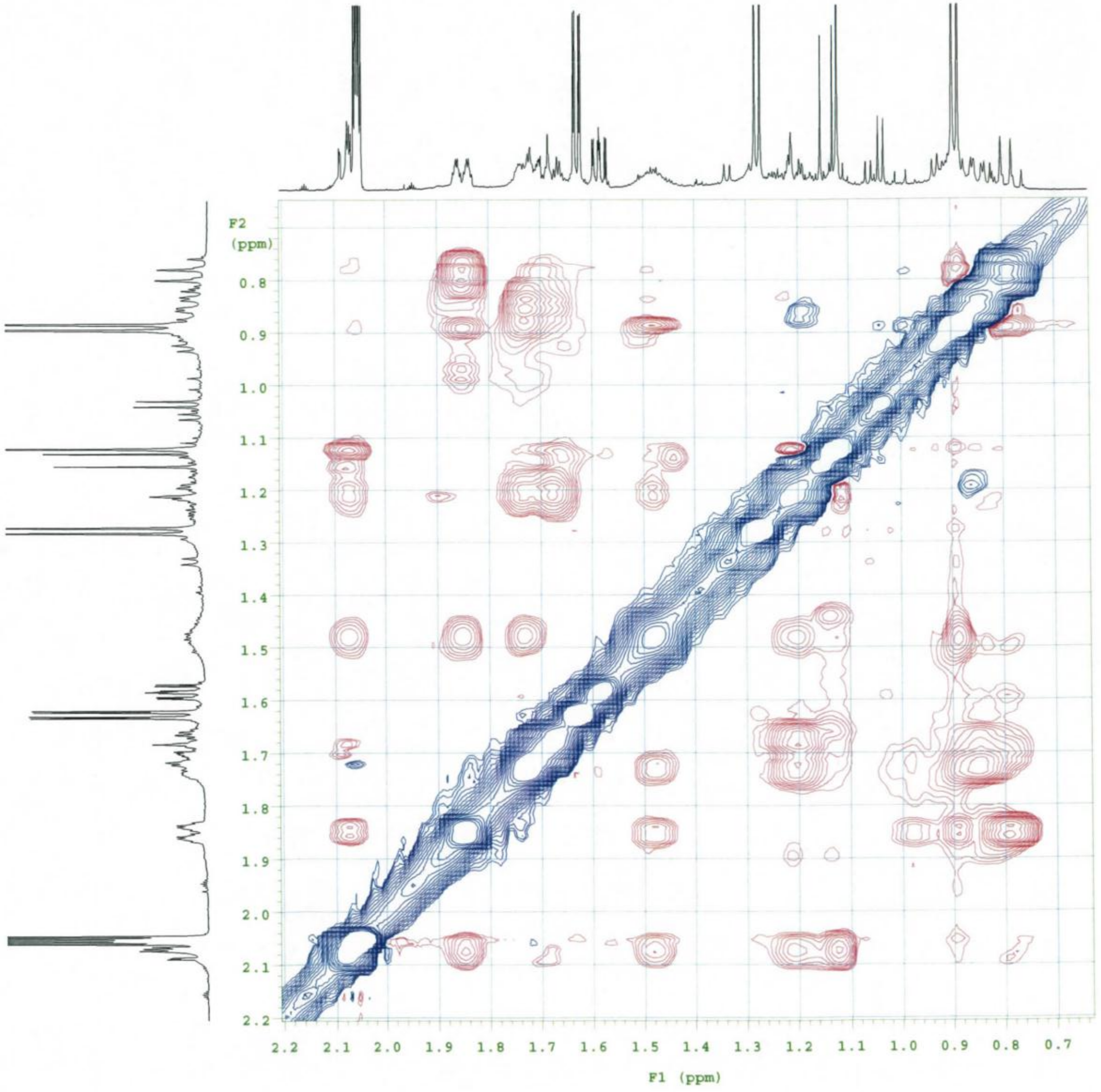

Figure S22 HMQC of 4

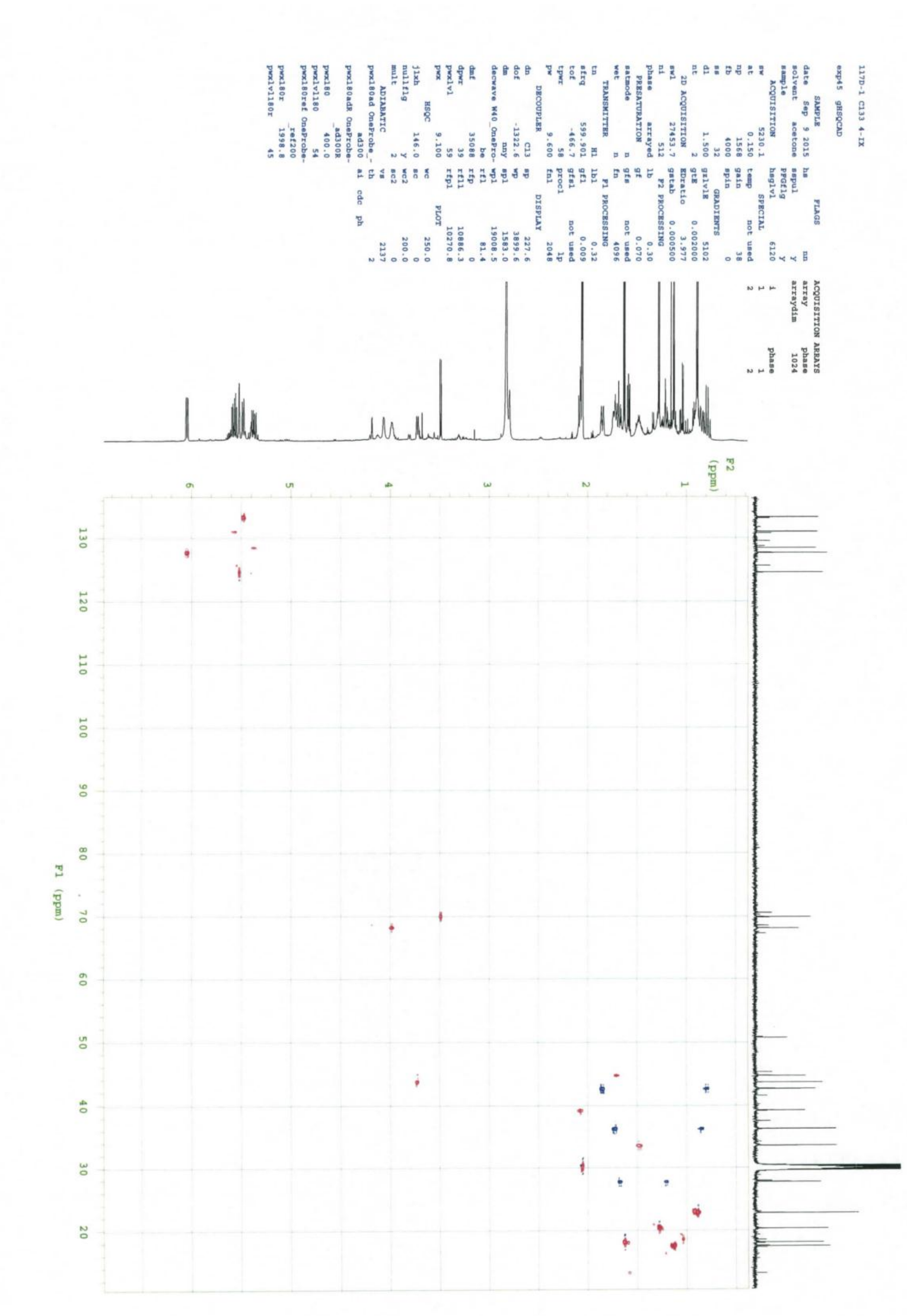

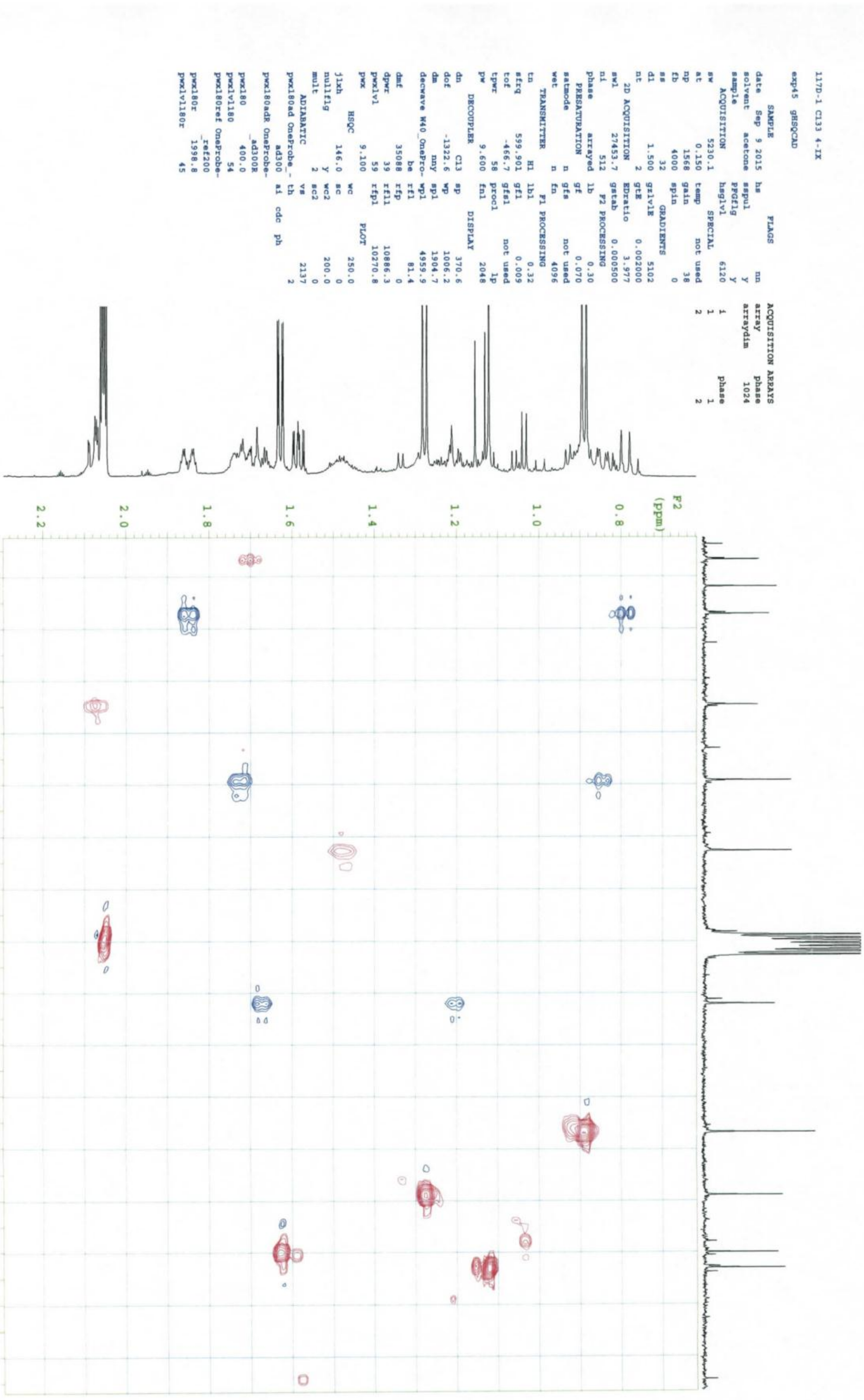
117D-1 C133 4-IX
exp45 gHSQCAD
F2 (ppm)
0.8
1.0
1.2
1.4
1.6
1.8
2.0
2.2
F1 (ppm)
45
40
35
30
25
20
15

Figure S23 HMBC of 4

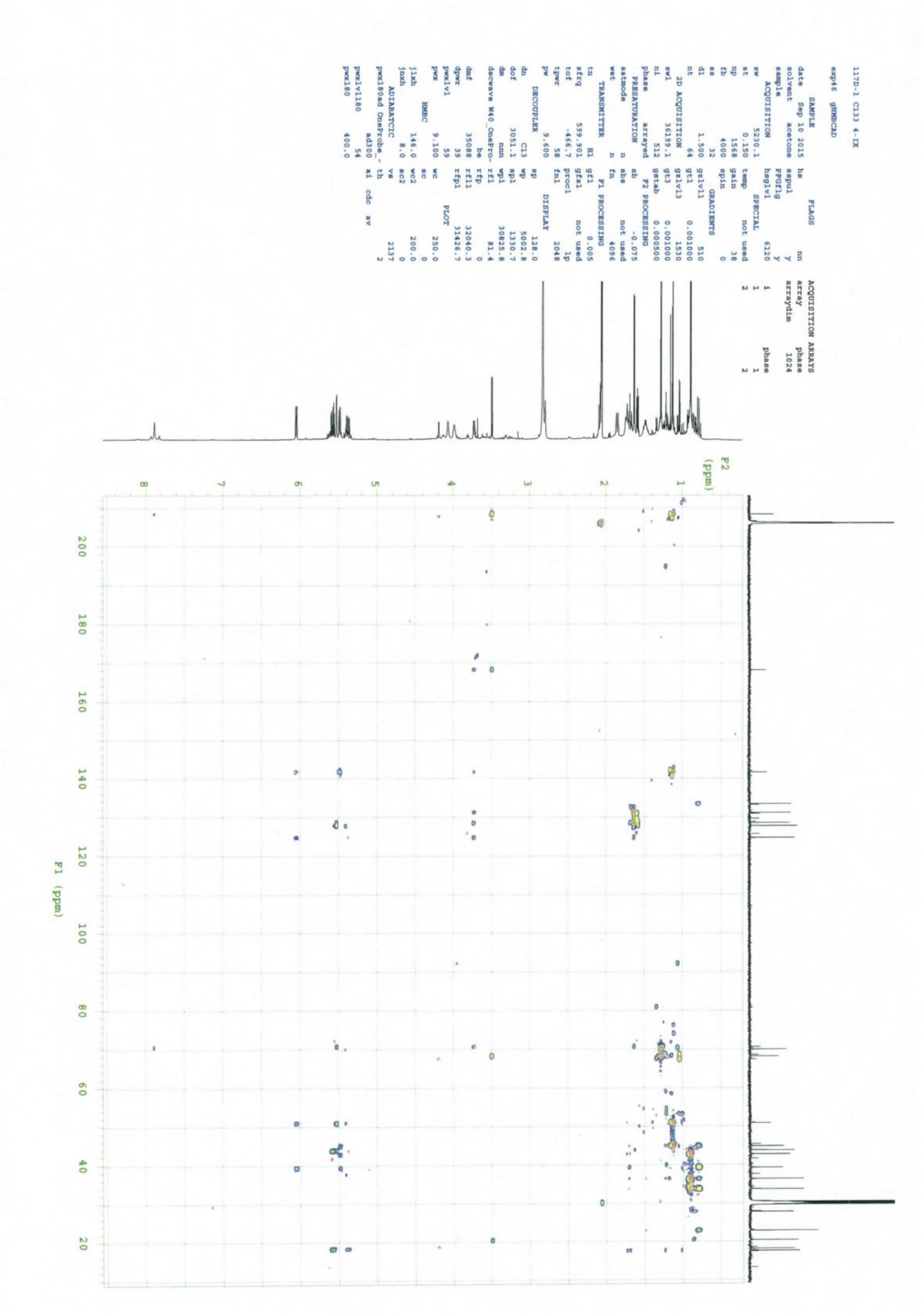

```
117D-1 C133 4-IX

exp46  gHMBCAD

      SAMPLE                FLAGS              ACQUISITION ARRAYS
date   Sep 10 2015  hs                 nn      array          phase
solvent     acetone  sspul              y      arraydim        1024
sample               PFGflg             y
   ACQUISITION       hsglvl          6120       i              phase
sw           5230.1       SPECIAL               1                  1
at            0.150  temp        not used       2                  2
np             1568  gain              38
fb             4000  spin               0
ss               32      GRADIENTS
d1            1.500  gzlvl1           510
nt               64  gt1         0.001000
  2D ACQUISITION     gzlvl3          1530
sw1         36199.1  gt3         0.001000
ni              512  gstab       0.000500
phase      arrayed     F2 PROCESSING
  PRESATURATION      sb            -0.075
satmode           n  sbs         not used
wet               n  fn              4096
   TRANSMITTER         F1 PROCESSING
tn               H1  gf1            0.005
sfrq        599.901  gfs1        not used
tof          -466.7  procl             lp
tpwr             58  fn1             2048
pw            9.600       DISPLAY
   DECOUPLER         sp             380.8
dn              C13  wp             978.1
dof          3051.1  sp1           2320.5
dm              nnn  wp1          10145.6
decwave W40_OnePro-  rfl             81.4
                 be  rfp                0
dmf           35088  rfl1         32040.3
dpwr             39  rfp1         31426.7
pwxlvl           59        PLOT
pwx           9.100  wc             250.0
      HMBC           sc                 0
j1xh          146.0  wc2            200.0
jnxh            8.0  sc2                0
    ADIABATCIC       vs              2137
pwx180ad OneProbe_-  th                 2
              ad300  ai   cdc   av
pwxlvl180        54
pwx180        400.0
```

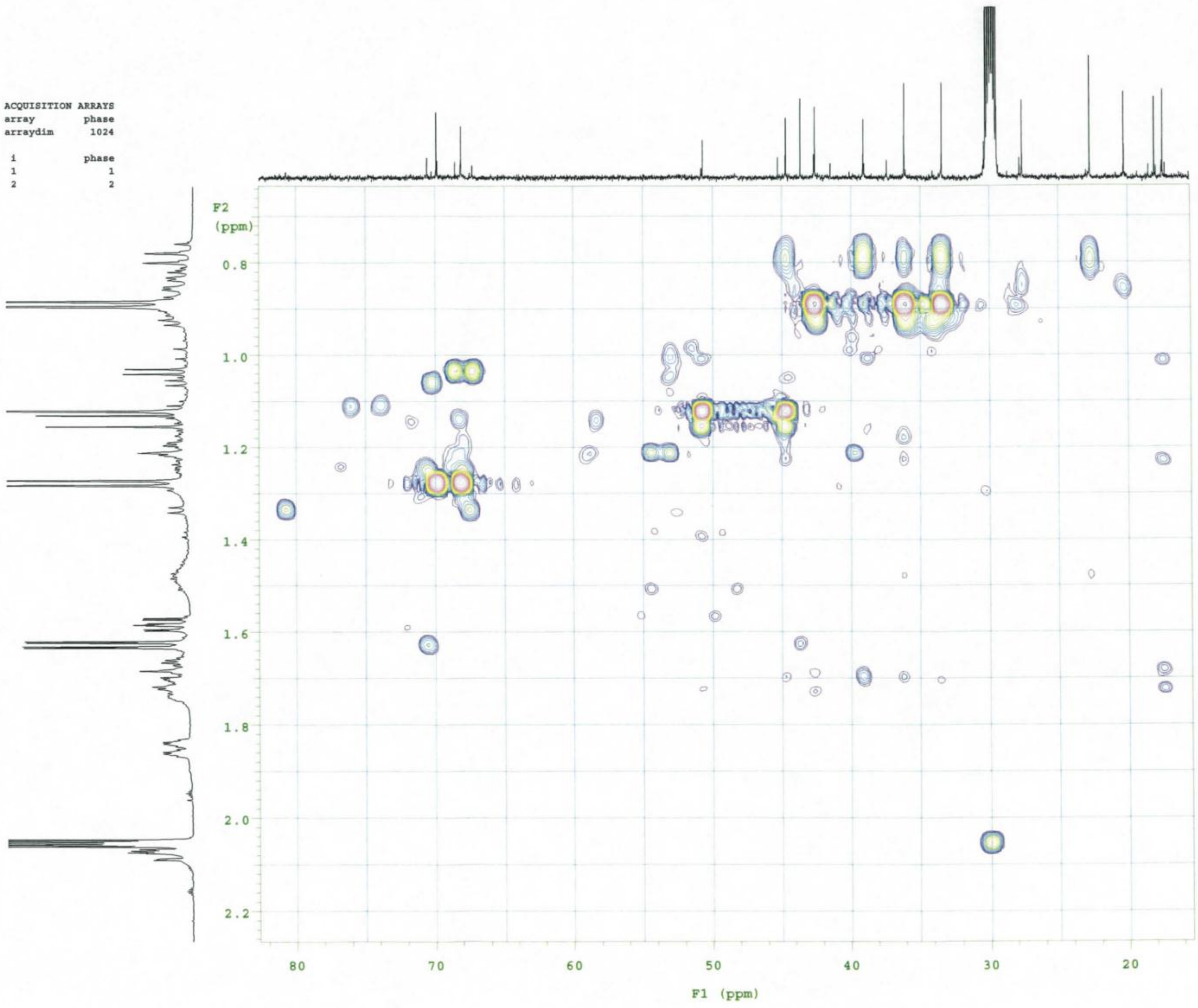

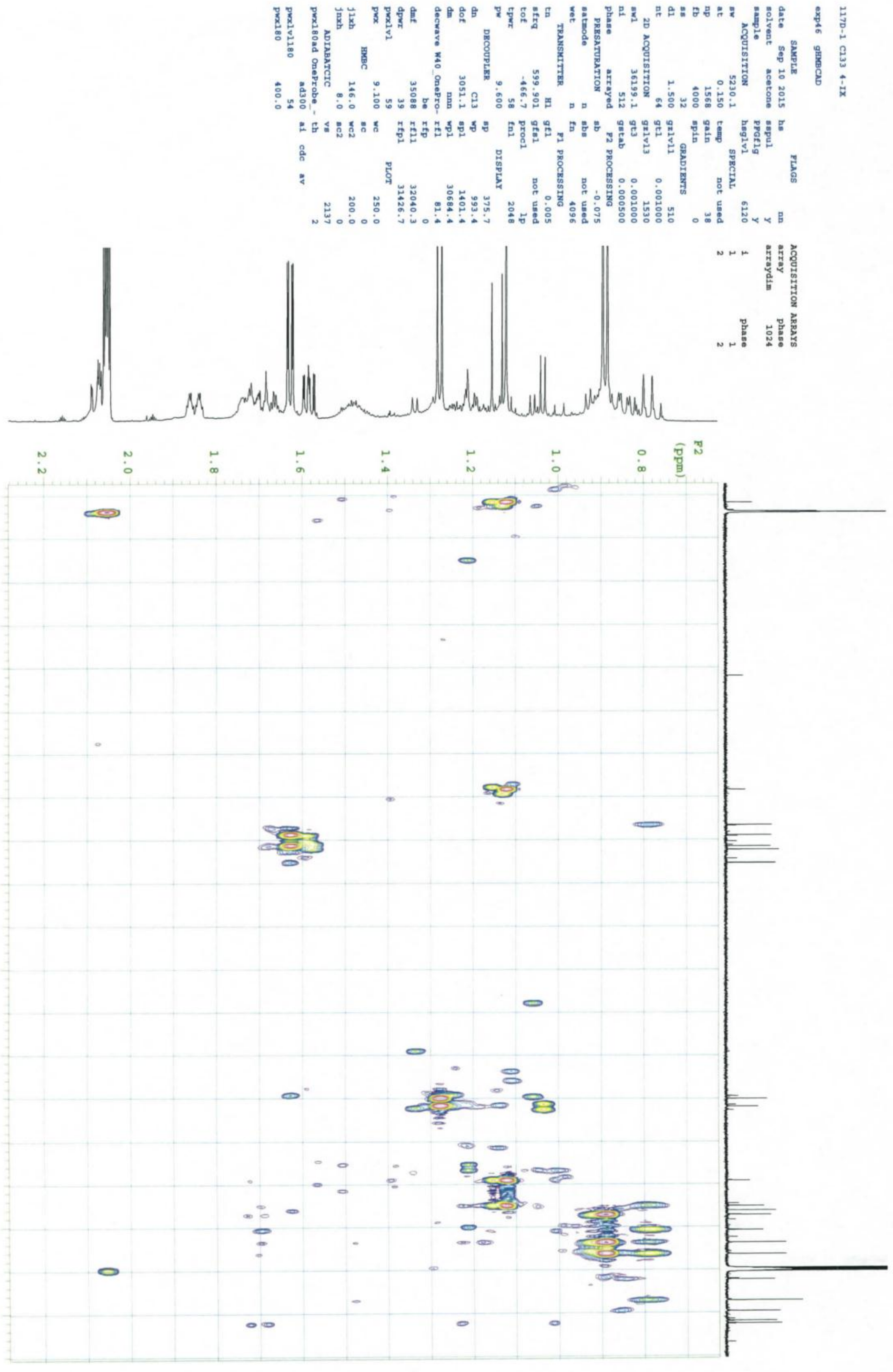

117D-1 C133 4-IX
exp46 gHMBCAD
SAMPLE
date Sep 10 2015
solvent acetone
sample
ACQUISITION
sw 5230.1
at 0.150
np 1568
fb 4000
ss 32
d1 1.500
nt 64
2D ACQUISITION
sw1 36199.1
ni 512
phase arrayed
PRESATURATION
satmode n
wet n
TRANSMITTER
tn H1
sfrq 599.901
tof -466.7
tpwr 58
pw 9.600
DECOUPLER
dn C13
dof 3051.1
dm nnn
decwave W40_OnePro-
be
dmf 35088
dpwr 39
pwxlvl 59
pwx 9.100
HMBC
j1xh 146.0
jnxh 8.0
ADIABATCIC
pwx180ad OneProbe_-
ad300
pwxlvl180 54
pwx180 400.0
FLAGS
hs nn
sspul y
PFGflg y
hsglvl 6120
SPECIAL
temp not used
gain 38
spin 0
GRADIENTS
gzlvl1 510
gt1 0.001000
gzlvl3 1530
gt3 0.001000
gstab 0.000500
F2 PROCESSING
sb -0.075
sbs not used
fn 4096
F1 PROCESSING
gf1 0.005
gfs1 not used
proc1 lp
fn1 2048
DISPLAY
sp 375.7
wp 993.4
sp1 1401.4
wp1 30684.4
rfl 81.4
rfp 0
rfl1 32040.3
rfp1 31426.7
PLOT
wc 250.0
sc 0
wc2 200.0
sc2 0
vs 2137
th 2
ai cdc av
ACQUISITION ARRAYS
array phase
arraydim 1024
i phase
1 1
2 2
F2 (ppm)
F1 (ppm)

Figure S24 FABMS of 4

[Elemental Composition] Page: 1
Data : 1601030 Date : 12-Jan-2016 16:47
Sample: 117D-1 C133-4-IX
Note : Matrix; NBA
Inlet : Direct Ion Mode : FAB+
RT : 7.66 min Scan#: (40,45)
Elements : C 30/20, H 35/25, N 3/0, O 6/0
Mass Tolerance : 20ppm, 1mmu if m/z > 50
Unsaturation (U.S.) : -1.0 - 30.0

Observed m/z	Int%	Err[ppm / mmu]	U.S.	Composition
398.2332	100.0	+0.1 / +0.0	9.5	C 24 H 32 N O 4

[Mass Spectrum]
Data : 1512135 Date : 22-Dec-2015 16:14
Sample: 117D-1 C133-4-IX
Note : Matrix; NBA
Inlet : Direct Ion Mode : FAB+
Spectrum Type : Normal Ion [MF-Linear]
RT : 1.98 min Scan# : (12,22)-k((32,42))[k=1.0]
BP : m/z 398.0000 Int. : 107.67
Output m/z range : 20.0000 to 1000.0000 Cut Level : 0.00 %

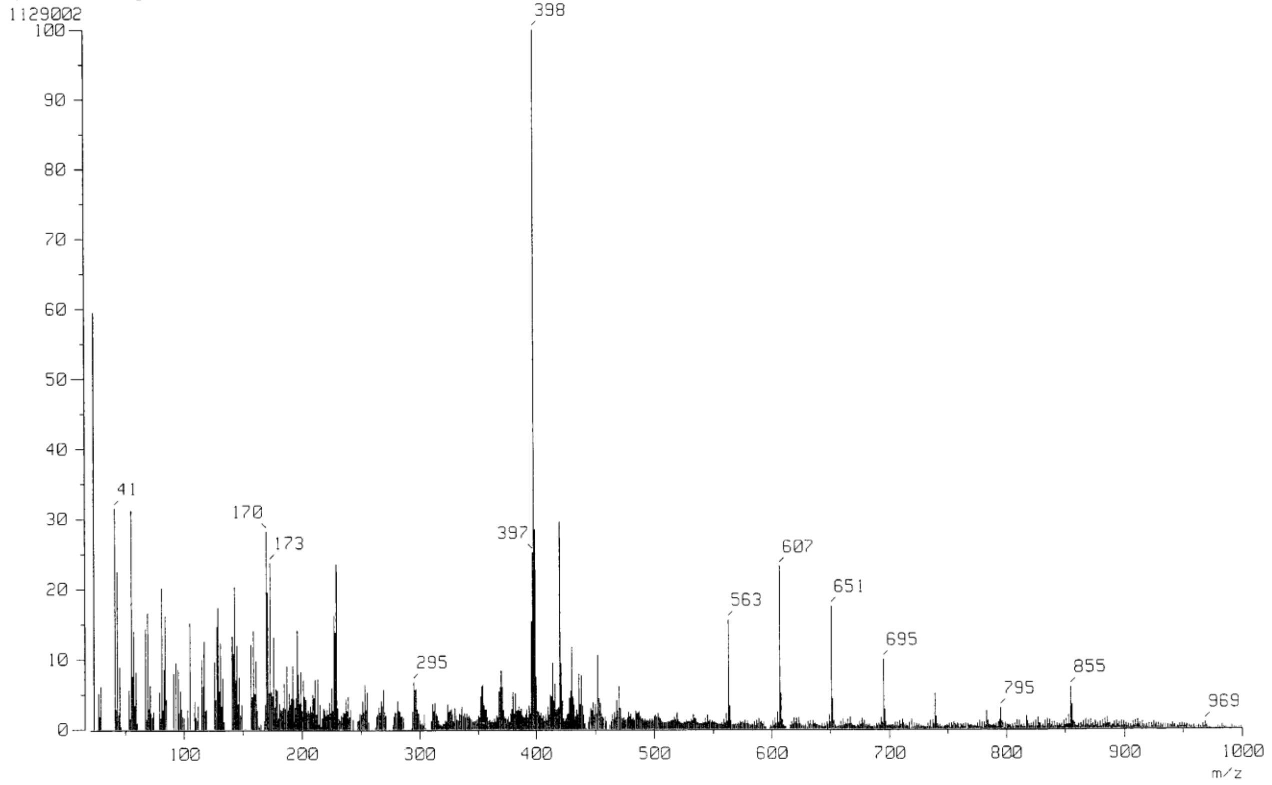

Supplement: Supplementary file 1 [file marinedrugs-17-00218-s001.zip › marinedrugs-480817 Suppl/S.M-R1/SM-vol4.pdf]
