# Supplementary material for: Altercrasins A–E, Decalin Derivatives, from a Sea-Urchin-Derived Alternaria sp.: Isolation and Structural Analysis Including Stereochemistry
Source: Mar Drugs. 2019 Apr 11;17(4):218. doi: 10.3390/md17040218 (PMC6521173; doi:10.3390/md17040218)
Supplement: Supplementary file 1 [file marinedrugs-17-00218-s001.zip › marinedrugs-480817 Suppl/S.M-R1/SM-vol5.pdf]

**Figure S25** **$^{1}H$ and $^{13}C$ NMR spectra in acetone-$d_6$ of 5**

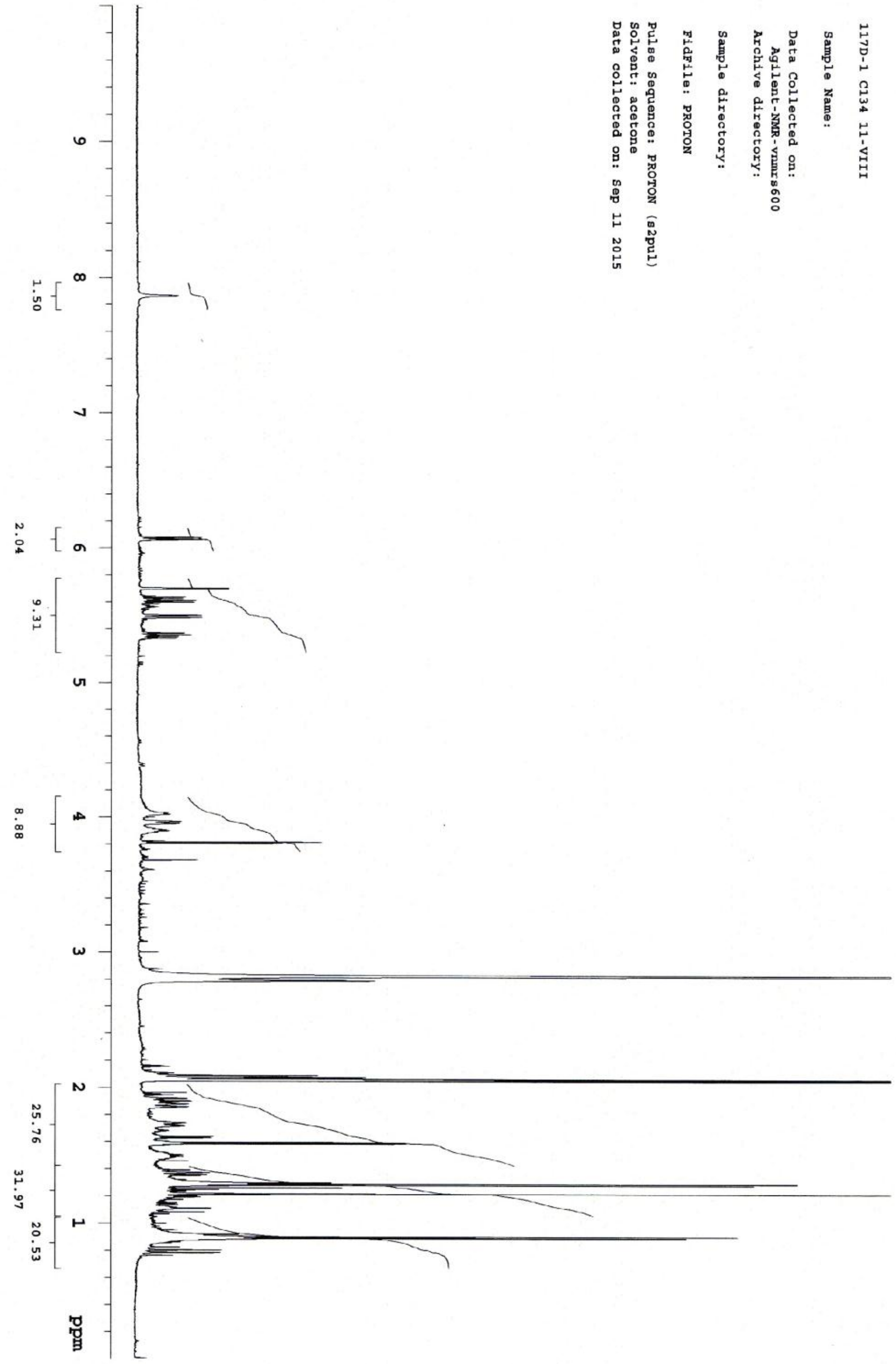

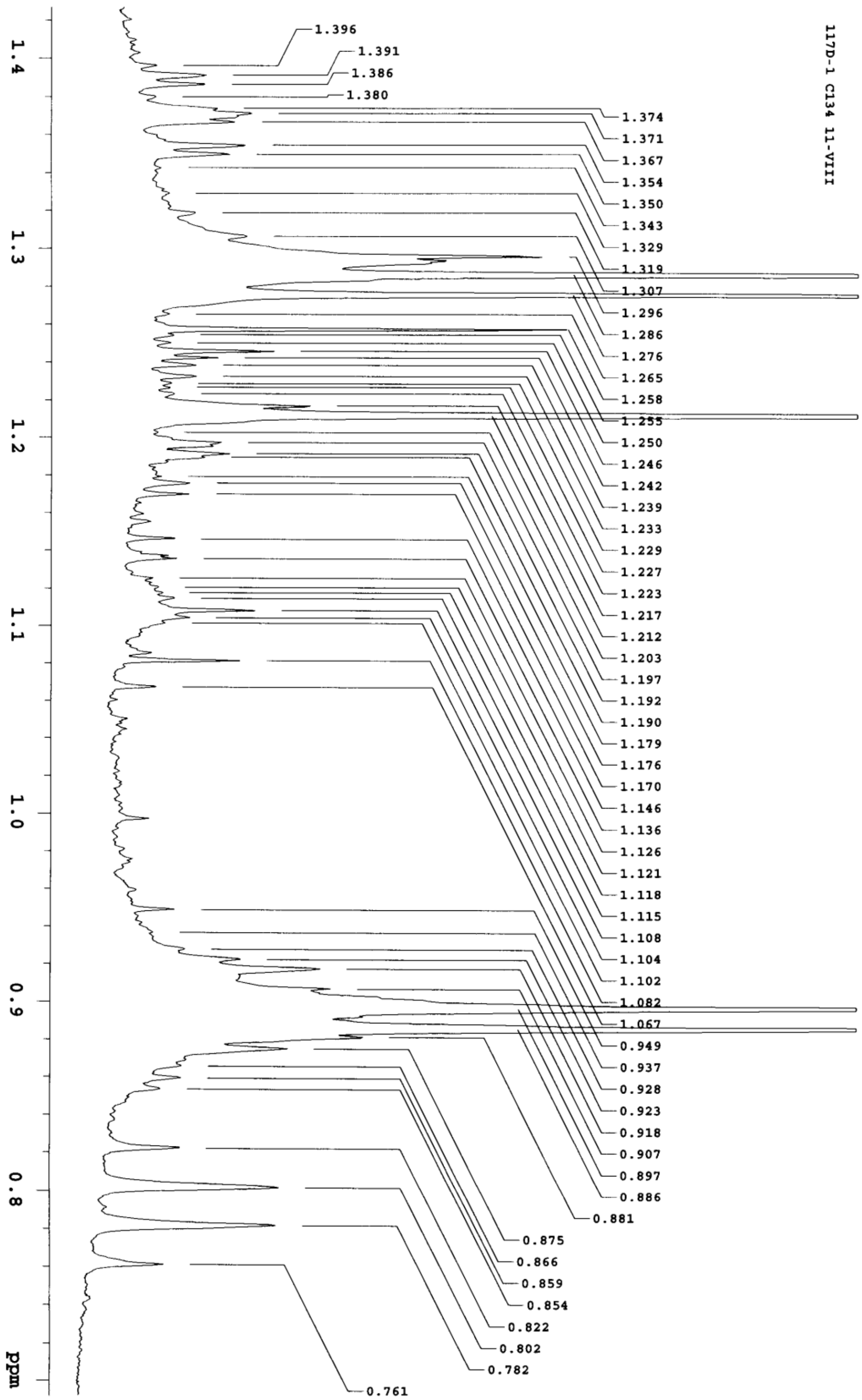
117D-1 C134 11-VIII
1.4
1.3
1.2
1.1
1.0
0.9
0.8
ppm
1.396
1.391
1.386
1.380
1.374
1.371
1.367
1.354
1.350
1.343
1.329
1.319
1.307
1.296
1.286
1.276
1.265
1.258
1.255
1.250
1.246
1.242
1.239
1.233
1.229
1.227
1.223
1.217
1.212
1.203
1.197
1.192
1.190
1.179
1.176
1.170
1.146
1.136
1.126
1.121
1.118
1.115
1.108
1.104
1.102
1.082
1.067
0.949
0.937
0.928
0.923
0.918
0.907
0.897
0.886
0.881
0.875
0.866
0.859
0.854
0.822
0.802
0.782
0.761

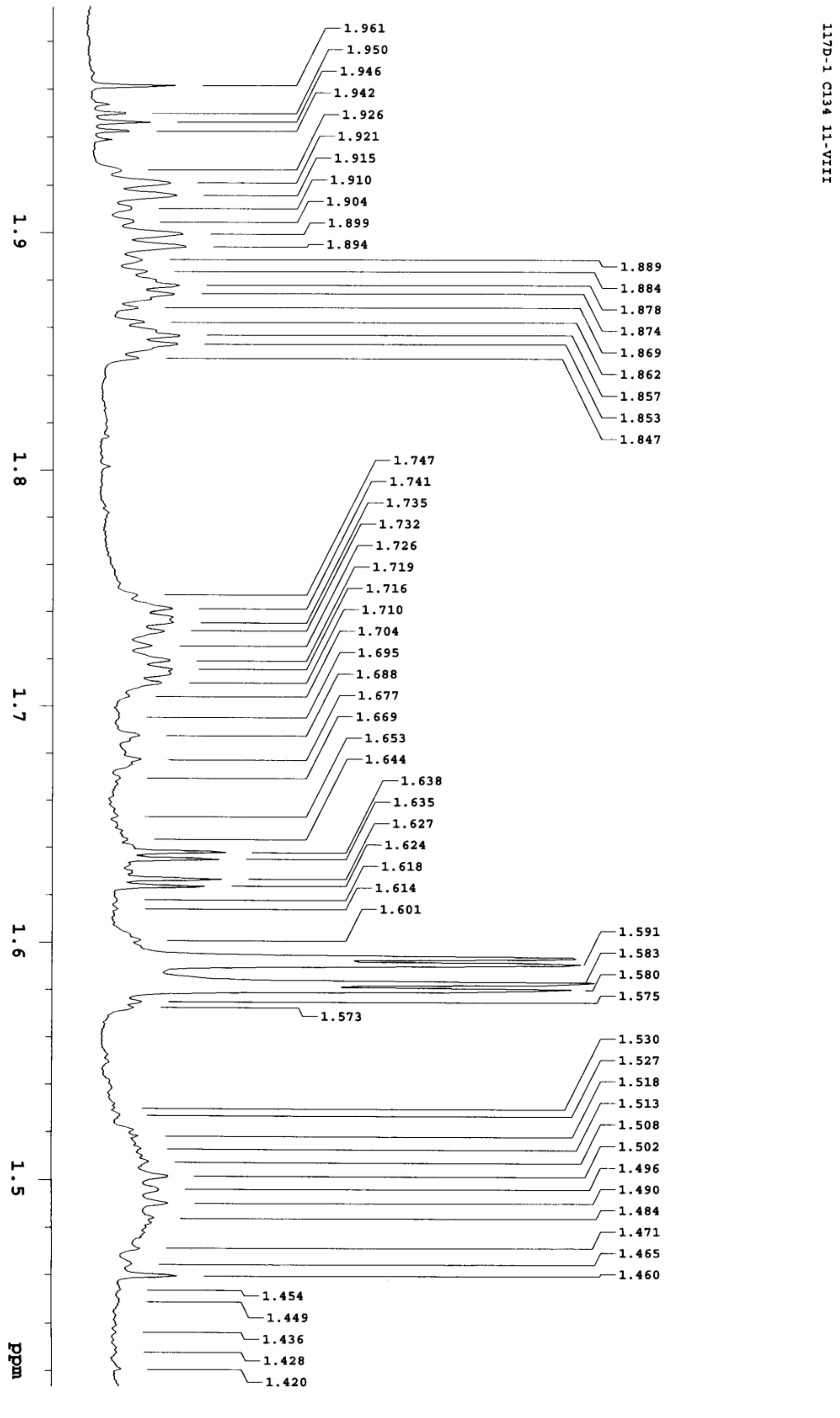
117D-1 C134 11-VIII
1.9
1.8
1.7
1.6
1.5
ppm
1.961
1.950
1.946
1.942
1.926
1.921
1.915
1.910
1.904
1.899
1.894
1.889
1.884
1.878
1.874
1.869
1.862
1.857
1.853
1.847
1.747
1.741
1.735
1.732
1.726
1.719
1.716
1.710
1.704
1.695
1.688
1.677
1.669
1.653
1.644
1.638
1.635
1.627
1.624
1.618
1.614
1.601
1.591
1.583
1.580
1.575
1.573
1.530
1.527
1.518
1.513
1.508
1.502
1.496
1.490
1.484
1.471
1.465
1.460
1.454
1.449
1.436
1.428
1.420

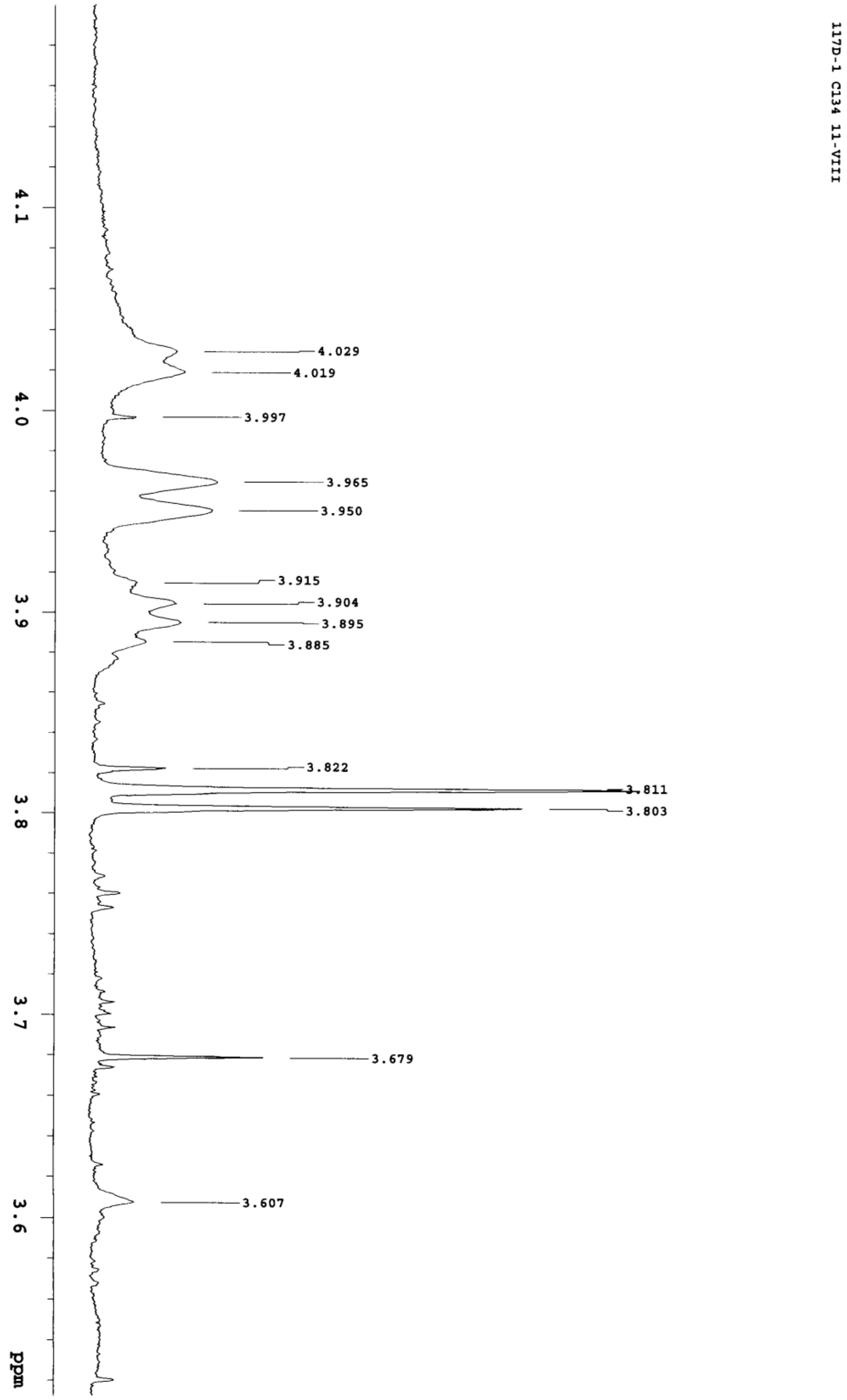
117D-1 C134 11-VIII
4.029
4.019
3.997
3.965
3.950
3.915
3.904
3.895
3.885
3.822
3.811
3.803
3.679
3.607
4.1
4.0
3.9
3.8
3.7
3.6
ppm

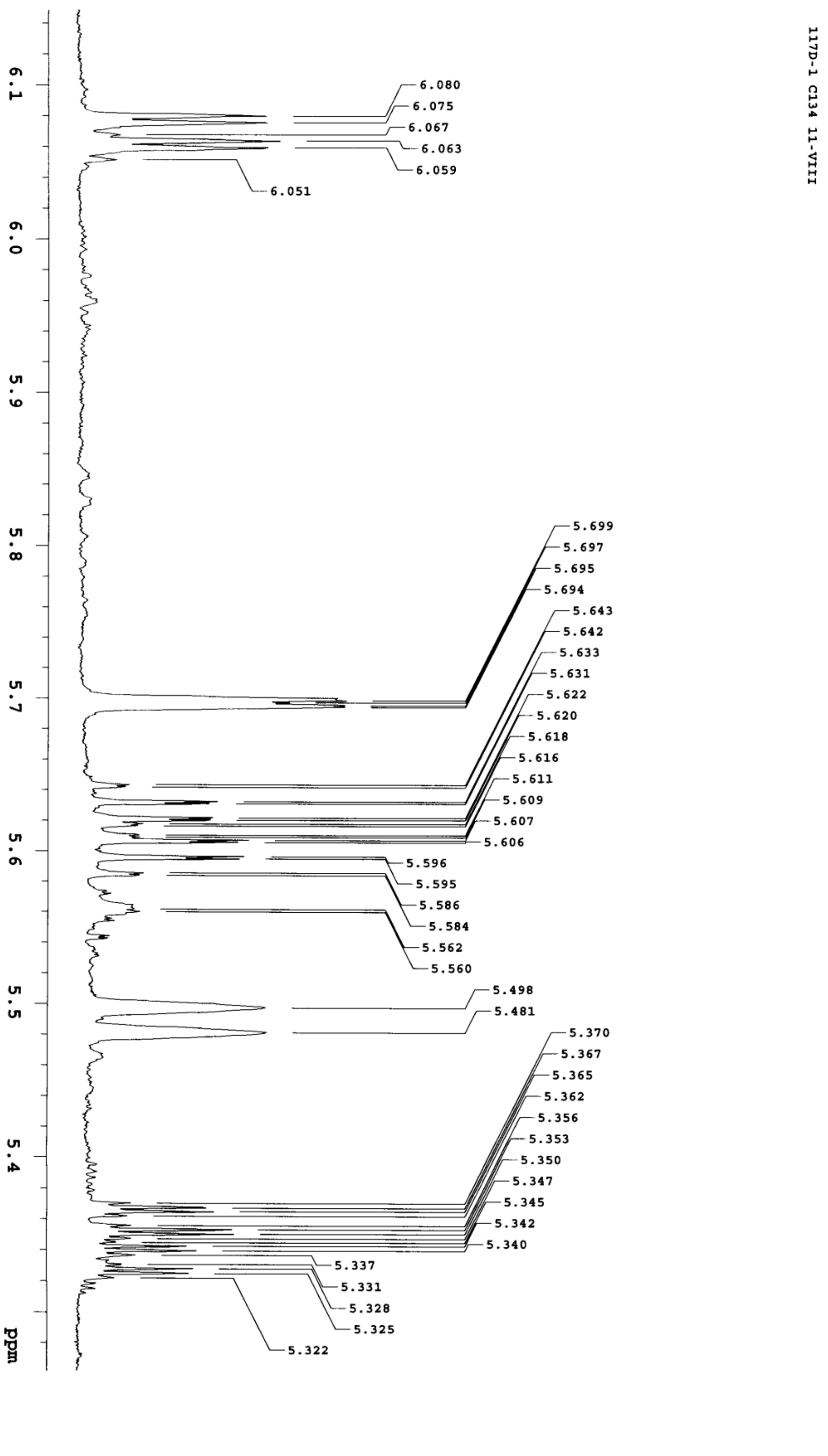
117D-1 C134 11-VIII
6.1
6.0
5.9
5.8
5.7
5.6
5.5
5.4
ppm
6.080
6.075
6.067
6.063
6.059
6.051
5.699
5.697
5.695
5.694
5.643
5.642
5.633
5.631
5.622
5.620
5.618
5.616
5.611
5.609
5.607
5.606
5.596
5.595
5.586
5.584
5.562
5.560
5.498
5.481
5.370
5.367
5.365
5.362
5.356
5.353
5.350
5.347
5.345
5.342
5.340
5.337
5.331
5.328
5.325
5.322

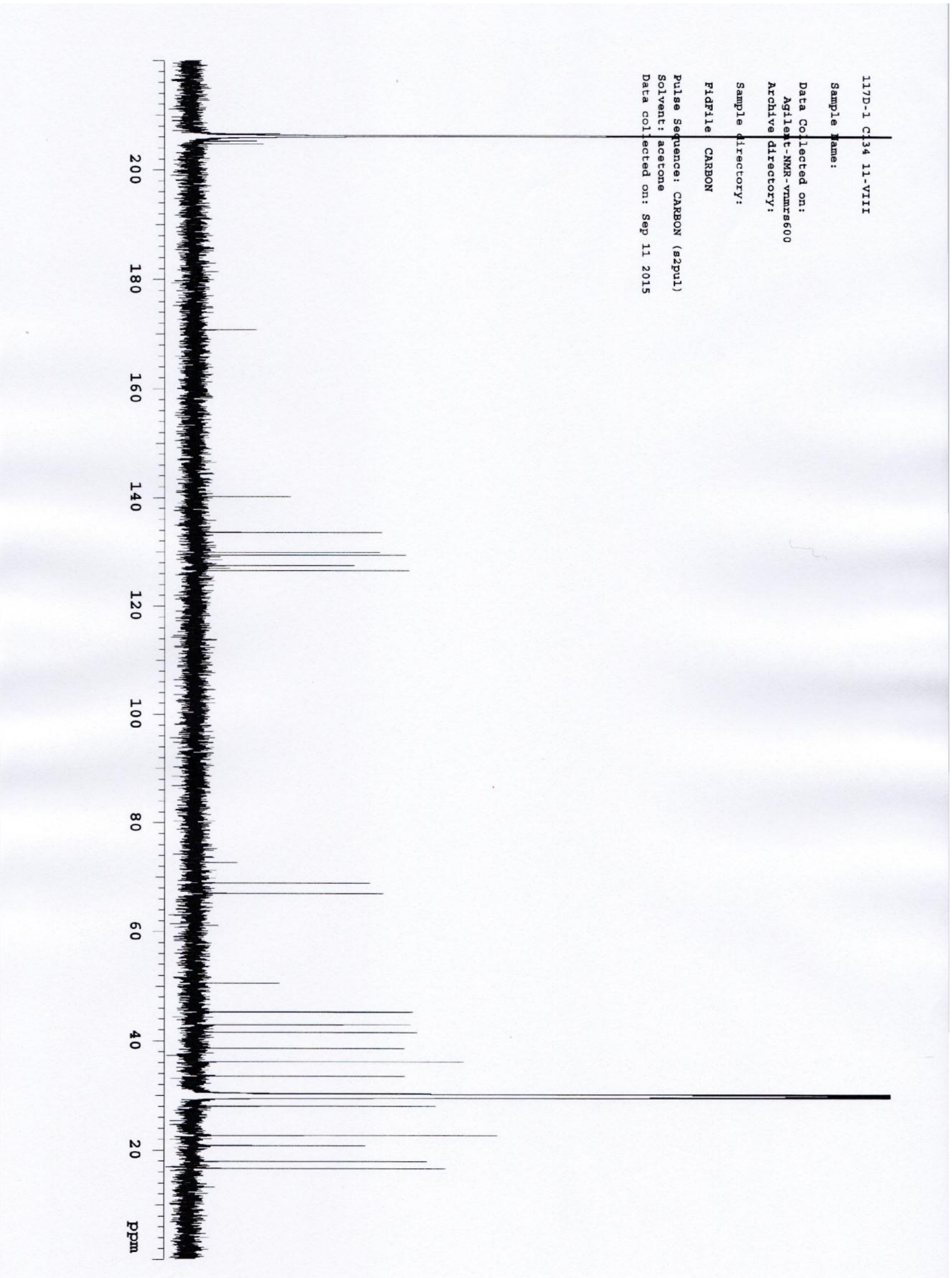
117D-1 C134 11-VIII
Sample Name:
Data Collected on:
Agilent-NMR-vnmrs600
Archive directory:
Sample directory:
FidFile CARBON
Pulse Sequence: CARBON (s2pul)
Solvent: acetone
Data collected on: Sep 11 2015
200
180
160
140
120
100
80
60
40
20
ppm

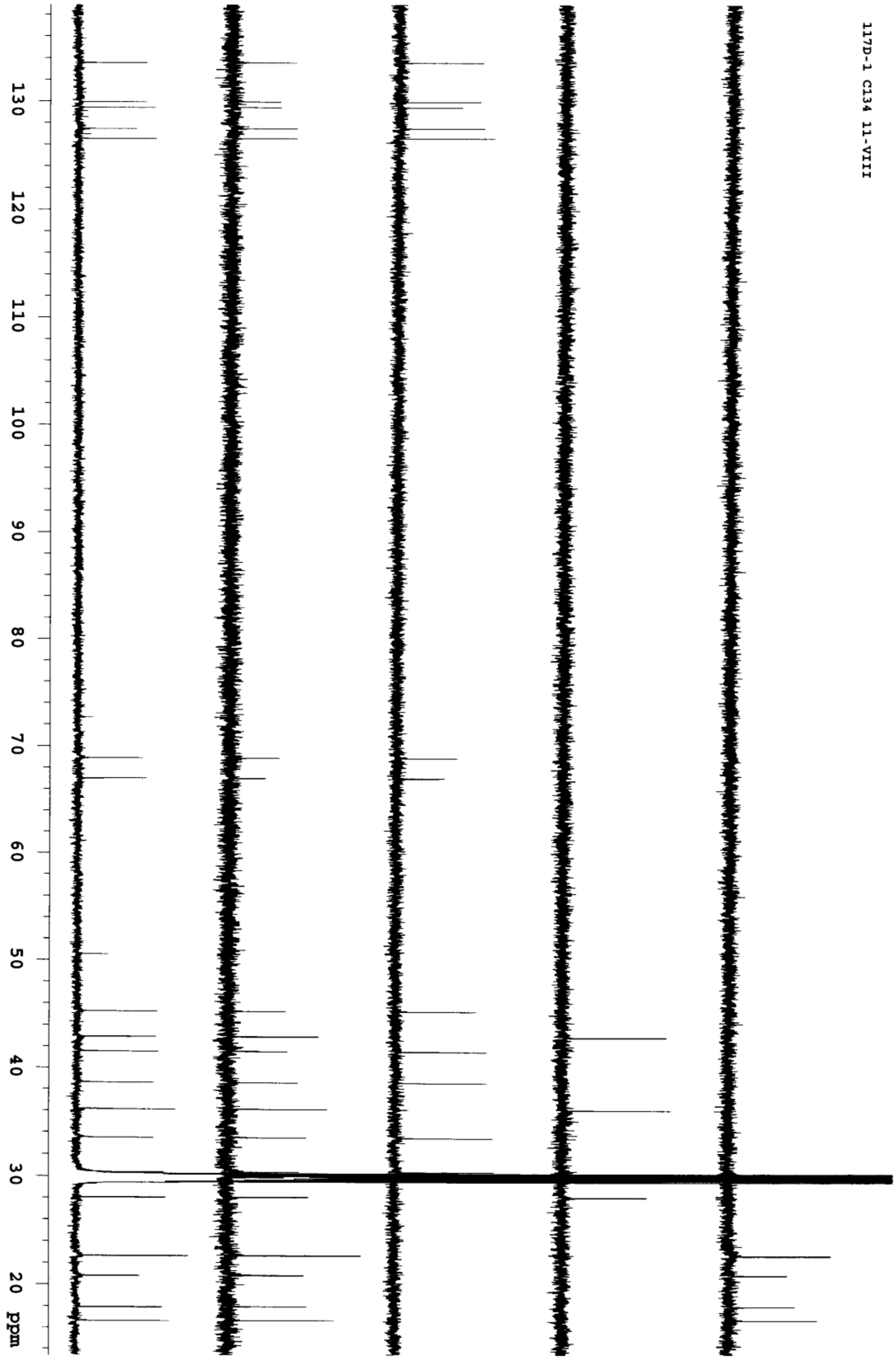
117D-1 C134 11-VIII
130
120
110
100
90
80
70
60
50
40
30
20
ppm

**Figure S26  $^{1}H$-$^{1}H$ COSY of 5**

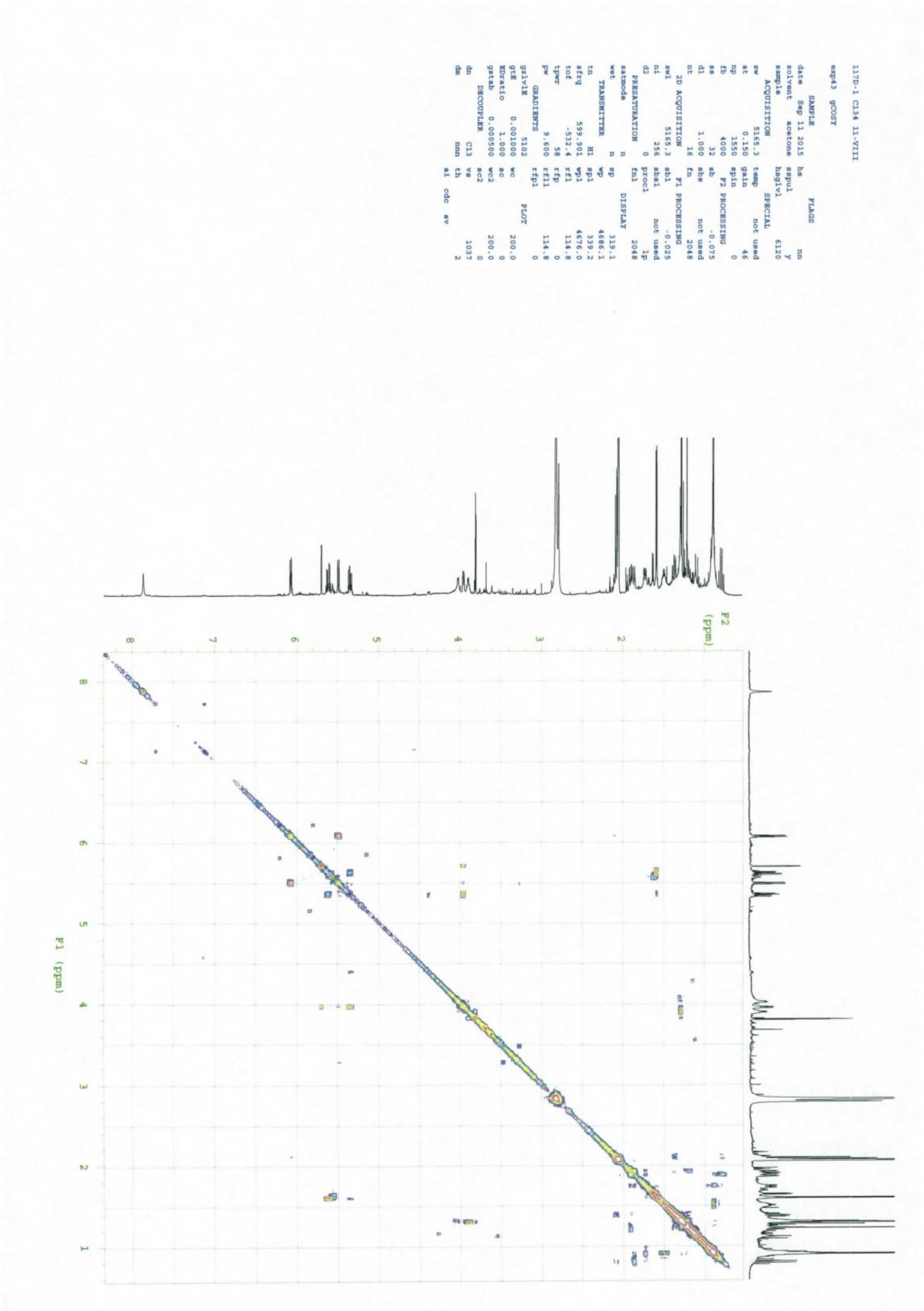

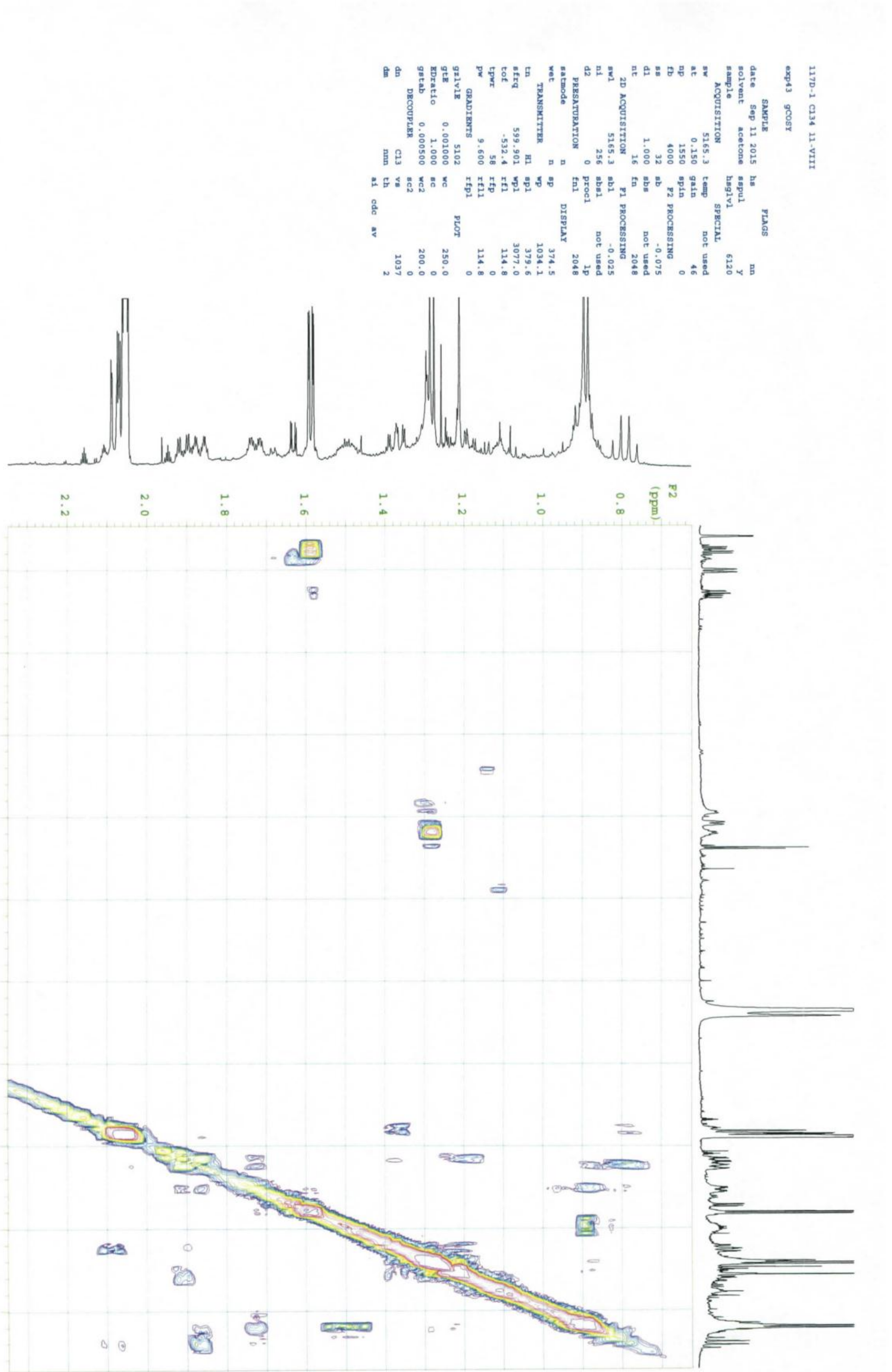

117D-1 C134 11-VIII
exp43 gCOSY
F2 (ppm)
F1 (ppm)

**Figure S27  NOESY of 5**

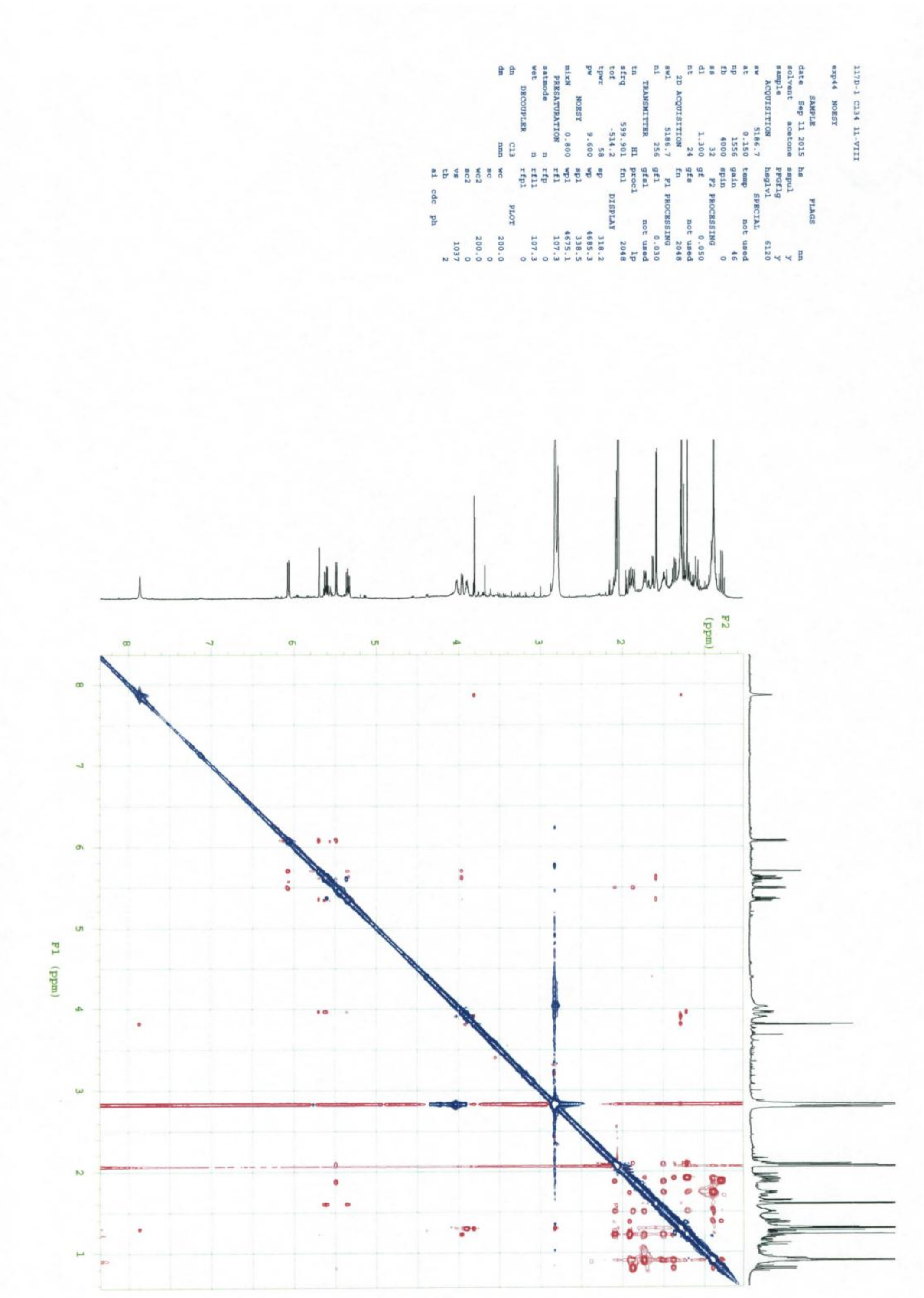

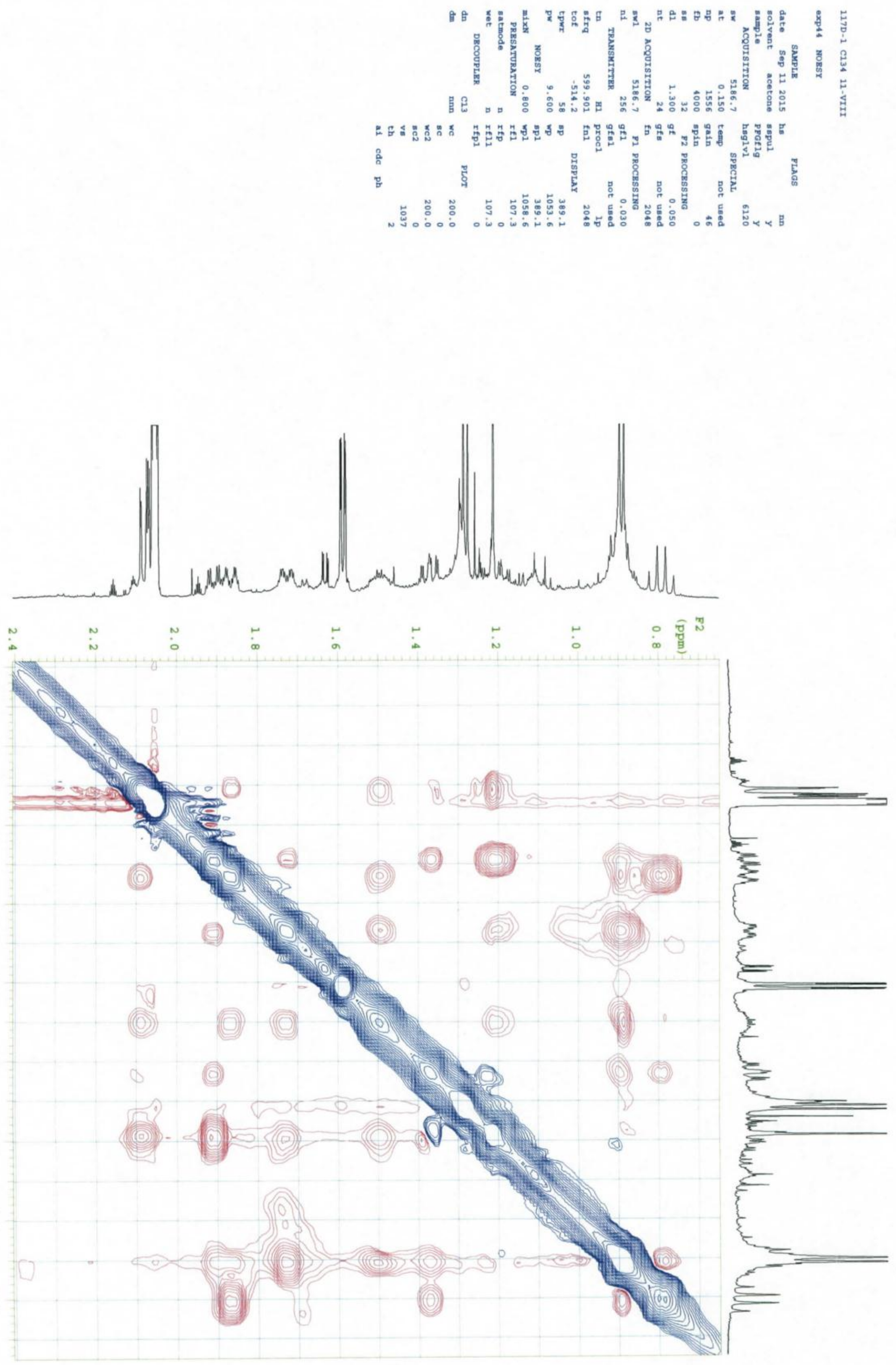

117D-1 C134 11-VIII
exp44 NOESY
SAMPLE
date Sep 11 2015
solvent acetone
sample
FLAGS
hs nn
sspul y
PFGflg y
hsglvl 6120
ACQUISITION
sw 5186.7
at 0.150
np 1556
fb 4000
ss 32
d1 1.300
nt 24
SPECIAL
temp not used
gain 46
spin 0
F2 PROCESSING
gf 0.050
gfs not used
fn 2048
2D ACQUISITION
sw1 5186.7
ni 256
F1 PROCESSING
gf1 0.030
gfs1 not used
proc1 lp
fn1 2048
TRANSMITTER
tn H1
sfrq 599.901
tof -514.2
tpwr 58
pw 9.600
DISPLAY
sp 389.1
wp 1053.6
sp1 389.1
wp1 1058.6
rfl 107.3
rfp 0
rfl1 107.3
rfp1 0
NOESY
mixN 0.800
PRESATURATION
satmode n
wet n
DECOUPLER
dn C13
dm nnn
PLOT
wc 200.0
sc 0
wc2 200.0
sc2 0
vs 1037
th 2
ai cdc ph
F2 (ppm)
F1 (ppm)

**Figure S28 HMQC of 5**

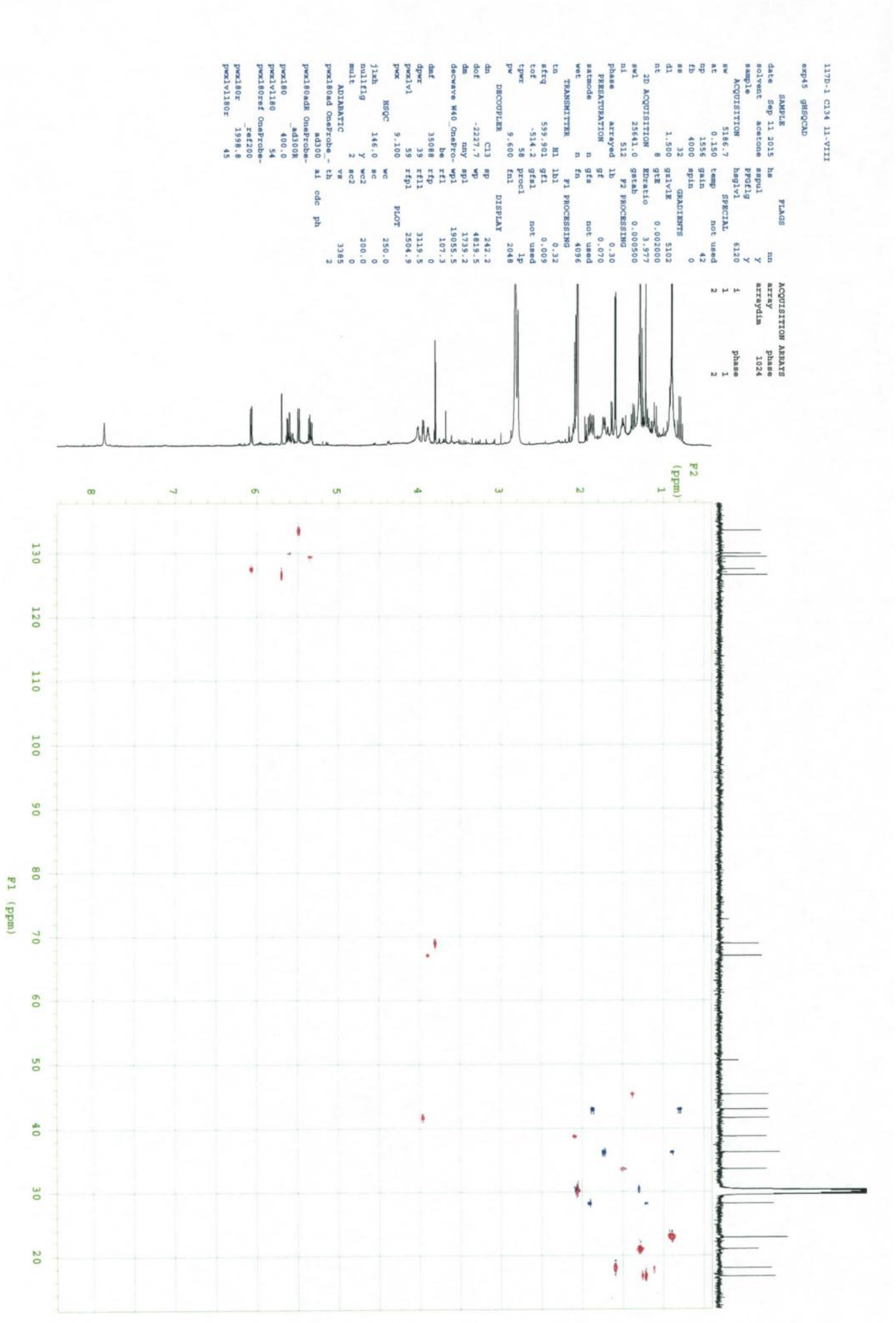

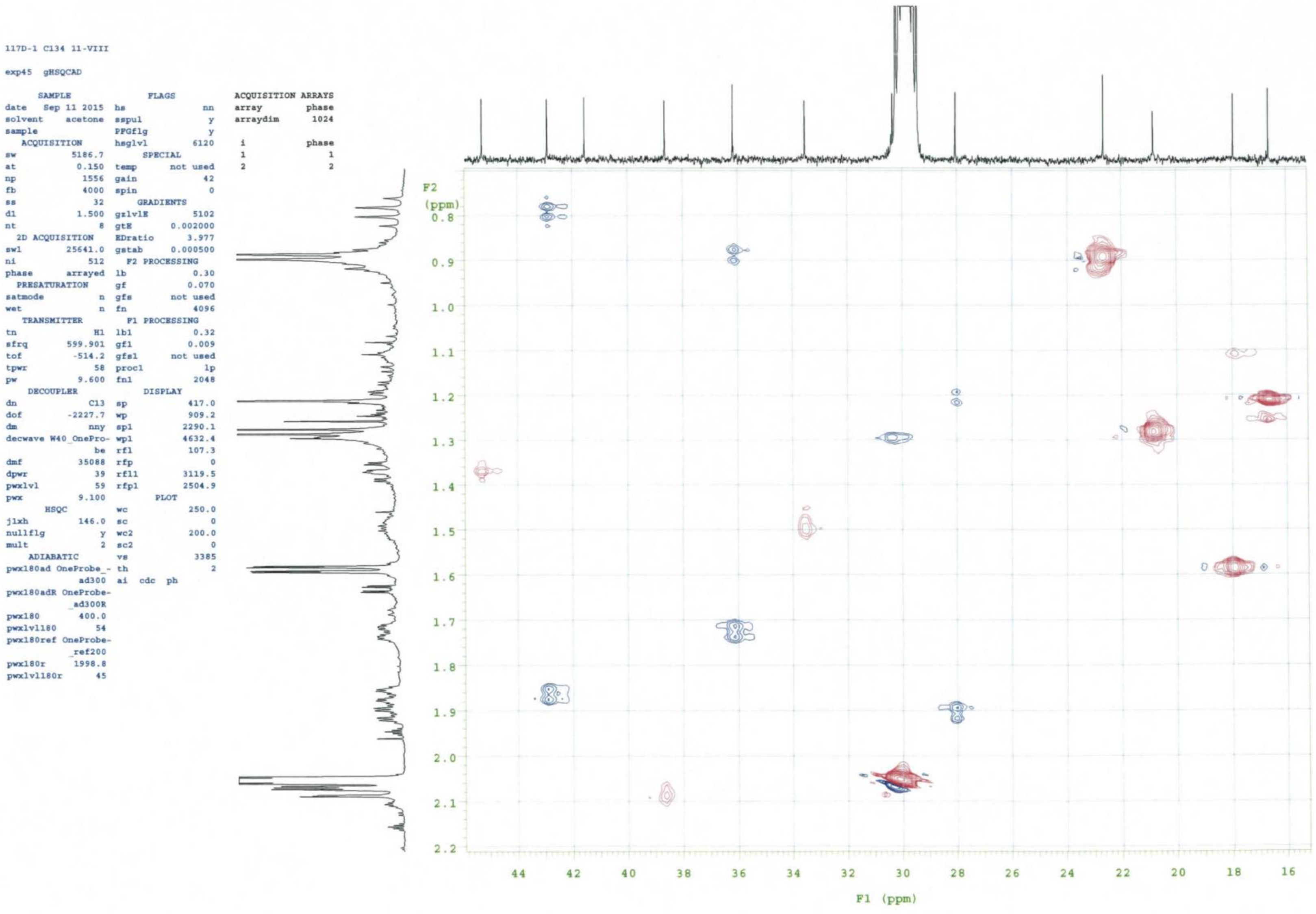
117D-1 C134 11-VIII
exp45 gHSQCAD
SAMPLE
date Sep 11 2015
solvent acetone
sample
ACQUISITION
sw 5186.7
at 0.150
np 1556
fb 4000
ss 32
d1 1.500
nt 8
2D ACQUISITION
sw1 25641.0
ni 512
phase arrayed
PRESATURATION
satmode n
wet n
TRANSMITTER
tn H1
sfrq 599.901
tof -514.2
tpwr 58
pw 9.600
DECOUPLER
dn C13
dof -2227.7
dm nny
decwave W40_OnePro-be
dmf 35088
dpwr 39
pwxlvl 59
pwx 9.100
HSQC
j1xh 146.0
nullflg y
mult 2
ADIABATIC
pwx180ad OneProbe_-ad300
pwx180adR OneProbe-_ad300R
pwx180 400.0
pwxlvl180 54
pwx180ref OneProbe-_ref200
pwx180r 1998.8
pwxlvl180r 45
FLAGS
hs nn
sspul y
PFGflg y
hsglvl 6120
SPECIAL
temp not used
gain 42
spin 0
GRADIENTS
gzlvlE 5102
gtE 0.002000
EDratio 3.977
gstab 0.000500
F2 PROCESSING
lb 0.30
gf 0.070
gfs not used
fn 4096
F1 PROCESSING
lb1 0.32
gf1 0.009
gfs1 not used
proc1 lp
fn1 2048
DISPLAY
sp 417.0
wp 909.2
sp1 2290.1
wp1 4632.4
rfl 107.3
rfp 0
rfl1 3119.5
rfp1 2504.9
PLOT
wc 250.0
sc 0
wc2 200.0
sc2 0
vs 3385
th 2
ai cdc ph
ACQUISITION ARRAYS
array phase
arraydim 1024
i phase
1 1
2 2
F2 (ppm)
F1 (ppm)

**Figure S29　HMBC of 5**

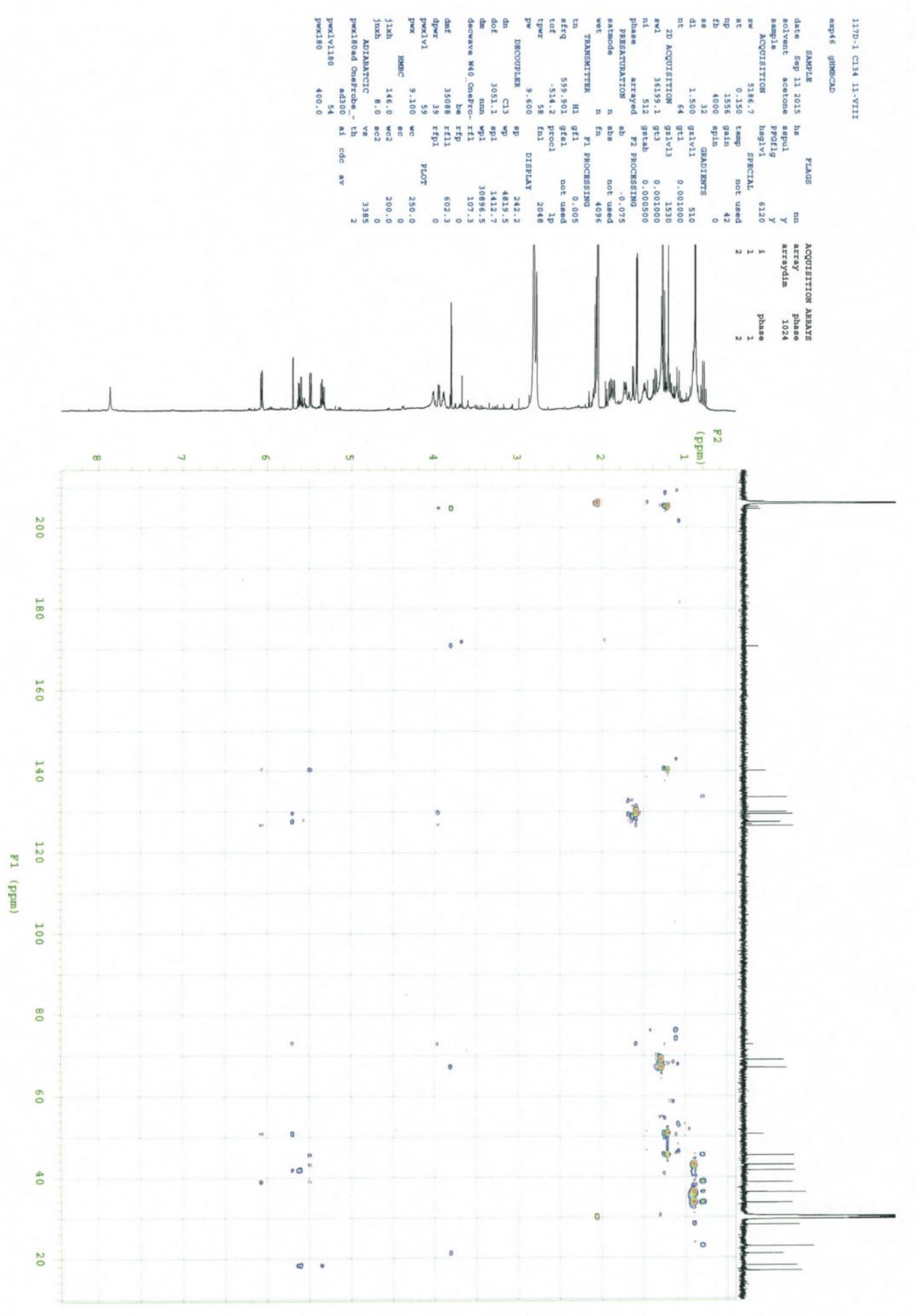

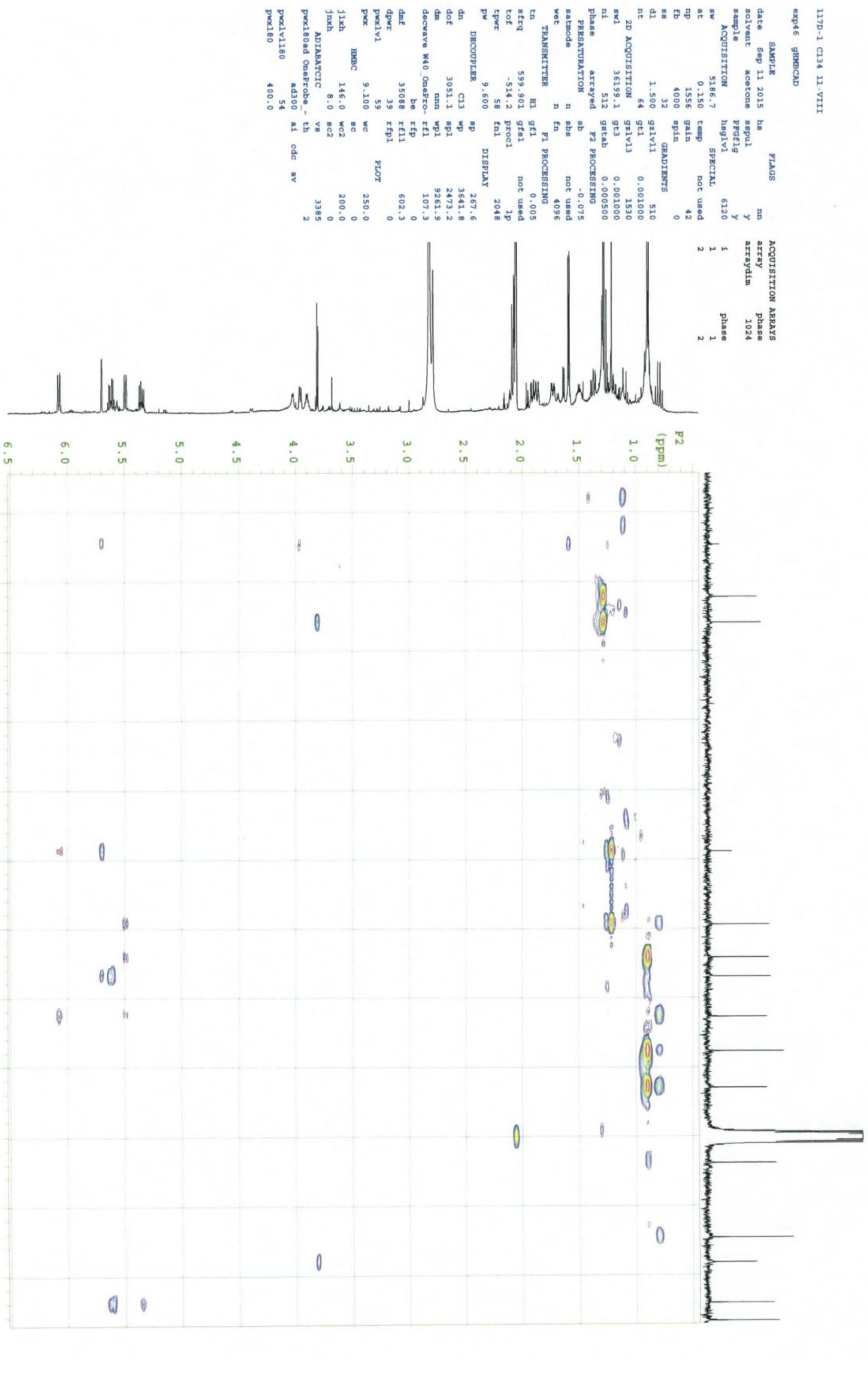
117D-1 C134 11-VIII
exp46 gHMBCAD
SAMPLE
date Sep 11 2015
solvent acetone
sample
ACQUISITION
sw 5186.7
at 0.150
np 1556
fb 4000
ss 32
d1 1.500
nt 64
2D ACQUISITION
sw1 36199.1
ni 512
phase arrayed
PRESATURATION
satmode n
wet n
TRANSMITTER
tn H1
sfrq 599.901
tof -514.2
tpwr 58
pw 9.600
DECOUPLER
dn C13
dof 3051.1
dm nnn
decwave W40_OnePro-be
dmf 35088
dpwr 39
pwxlvl 59
pwx 9.100
HMBC
j1xh 146.0
jnxh 8.0
ADIABATCIC
pwx180ad OneProbe_-ad300
pwxlvl180 54
pwx180 400.0
FLAGS
hs nn
sspul y
PFGflg y
hsglvl 6120
SPECIAL
temp not used
gain 42
spin 0
GRADIENTS
gzlvl1 510
gt1 0.001000
gzlvl3 1530
gt3 0.001000
gstab 0.000500
F2 PROCESSING
sb -0.075
sbs not used
fn 4096
F1 PROCESSING
gf1 0.005
gfs1 not used
proc1 lp
fn1 2048
DISPLAY
sp 267.6
wp 3641.8
sp1 2473.2
wp1 9261.9
rfl 107.3
rfp 0
rfl1 602.3
rfp1 0
PLOT
wc 250.0
sc 0
wc2 200.0
sc2 0
vs 3385
th 2
ai cdc av
ACQUISITION ARRAYS
array phase
arraydim 1024
i phase
1 1
2 2
F2 (ppm)
F1 (ppm)

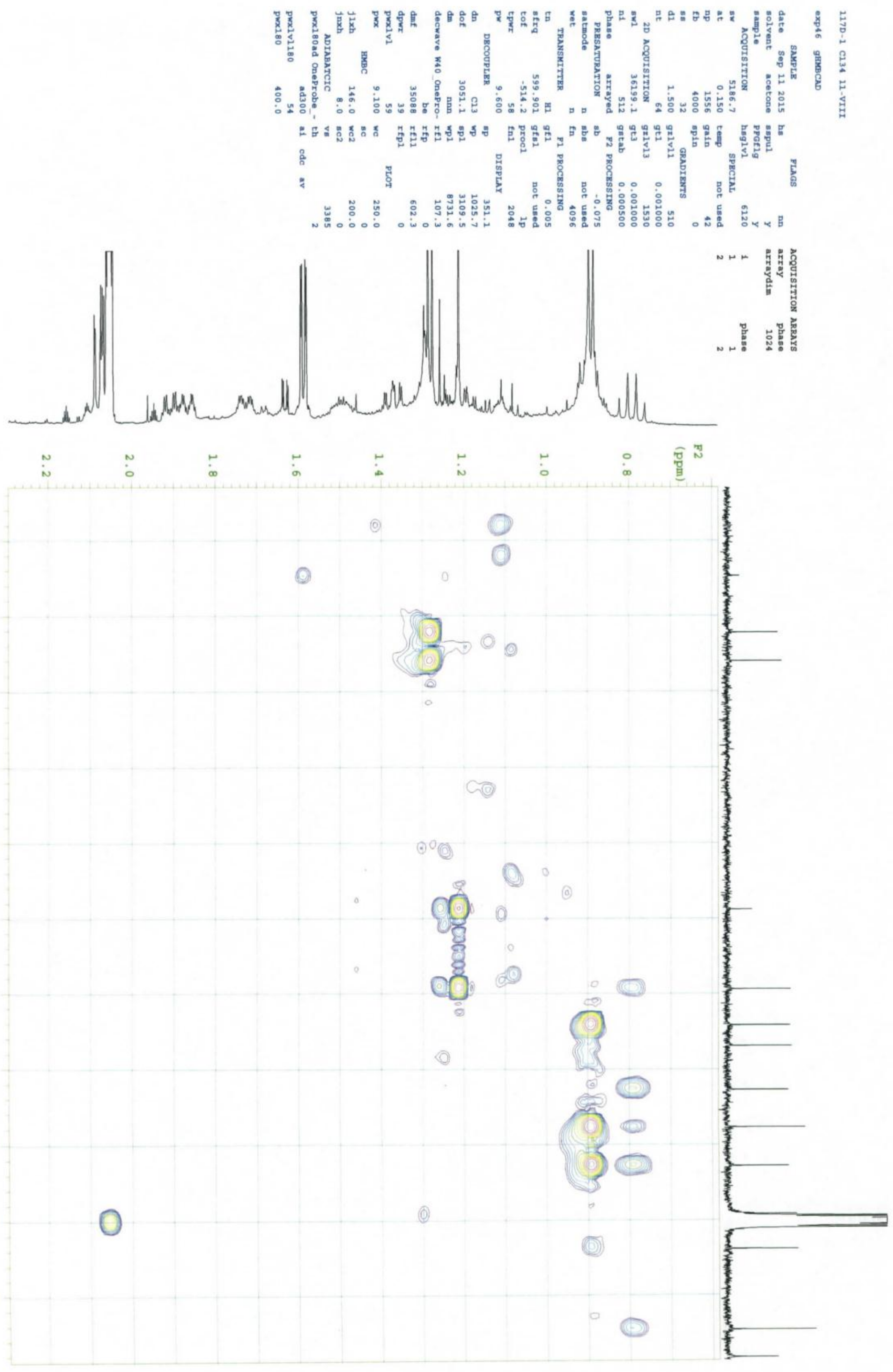
117D-1 C134 11-VIII
exp46 gHMBCAD
F2 (ppm)
F1 (ppm)

**Figure S30　FABMS of 5**

```
[ Elemental Composition ]                                           Page: 1
Data   : 1601029                    Date : 12-Jan-2016 16:38
Sample: 117D-1 C134-11-VIII
Note : Matrix; NBA
Inlet : Direct                      Ion Mode : FAB+
RT : 7.29 min                       Scan#: (38,43)
Elements : C 30/20, H 35/25, N 3/0, O 6/0
Mass Tolerance       : 20ppm, 1mmu if m/z > 50
Unsaturation (U.S.) : -1.0 - 30.0

Observed m/z  Int%   Err[ppm / mmu]   U.S.  Composition
  398.2332   100.0     +0.1 /  +0.0    9.5  C 24 H 32 N O 4
```

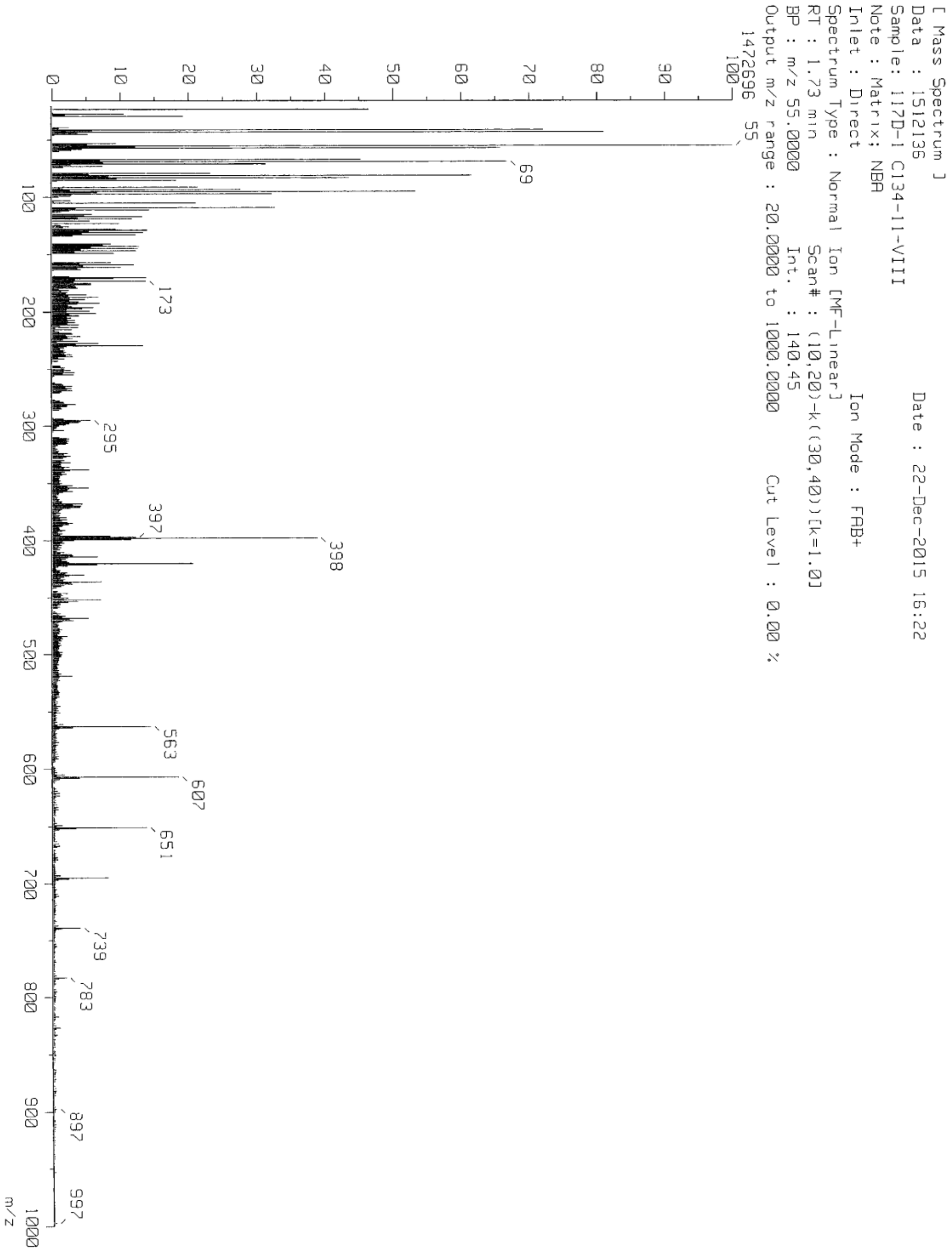
[ Mass Spectrum ]
Data : 1512136
Date : 22-Dec-2015 16:22
Sample: 117D-1 C134-11-VIII
Note : Matrix; NBA
Inlet : Direct
Ion Mode : FAB+
Spectrum Type : Normal Ion [MF-Linear]
RT : 1.73 min
Scan# : (10,20)-k((30,40))[k=1.0]
BP : m/z 55.0000
Int. : 140.45
Output m/z range : 20.0000 to 1000.0000
Cut Level : 0.00 %
1472696
55
69
173
295
397
398
563
607
651
739
783
897
997
m/z
